# PML-driven sumoylation of PML::RARA-bound repressors drives hematopoietic progenitor immortalization

**DOI:** 10.1084/jem.20252333

**Published:** 2026-08-28

**Authors:** Hsin Chieh Wu, Emmanuel Laplantine, Cécile Esnault, Michiko Niwa-Kawakita, Yi Zhang, Viviane De Almeida Bastos, Marie-Claude Geoffroy, Hugues de Thé

**Affiliations:** 1 Collège de France, Oncologie Cellulaire et Moléculaire, PSL University, CIRB, Institut National de la Santé et de la Recherche Médicale UMR 1050, Centre National de la Recherche Scientifique UMR 7241, Equipe Labellisée par la Ligue contre le Cancer, Paris, France; 2 Université Paris-Cité, Institut National de la Santé et de la Recherche Médicale U1342, Centre National de la Recherche Scientifique EMR8000, Institut de Recherche Saint-Louis, Hôpital Saint-Louis, Paris, France; 3 Sorbonne Université, UMS 37 PASS, P3S, Institut National de la Santé et de la Recherche Médicale, Paris, France; 4 Laboratoire d’hématologie Moléculaire, Assistance Publique–Hôpitaux de Paris, Hôpital St. Louis, Paris, France

## Abstract

While acute promyelocytic leukemia (APL) is always driven by fusions involving one of the three retinoic acid receptors, why PML and RARA are the preferred fusion partners has remained largely unsettled. Here, we demonstrate that corepressor (NCoR) binding onto the RARA moiety of PML::RARA is required for hematopoietic progenitor immortalization. We establish that PML-mediated tethering of the UBC9 SUMO-conjugating enzyme onto PML::RARA enforces SUMO2 conjugation of multiple RARA partner proteins, notably the NCoR complex, boosting its repressive power. PML mutants that fail to recruit UBC9 yield PML::RARA fusions that promote neither NCoR sumoylation nor transformation. Conversely, direct UBC9/RARA fusion drives both efficient corepressor complex sumoylation and immortalization. Sumoylation inhibitors reactivate retinoic acid target genes in PML::RARA-expressing, but not in RARA-expressing, progenitors and trigger APL differentiation. Thus, fusion of PML to RARA entails an unexpected gain of function that boosts RARA-mediated transcriptional repression through sumoylation of PML::RARA-bound proteins, explaining the recurrent implication of PML and RARA in APL pathogenesis.

## Teaser

This study reveals that PML::RARA drives acute promyelocytic leukemia by bridging the PML-bound SUMO machinery and RARA-bound corepressors, enhancing corepressor sumoylation, target gene repression, and the differentiation block. Sumoylation inhibition reactivates downstream targets, triggers myeloid maturation, and blunts progenitor self-renewal.

## Graphical Abstract

**Figure:**
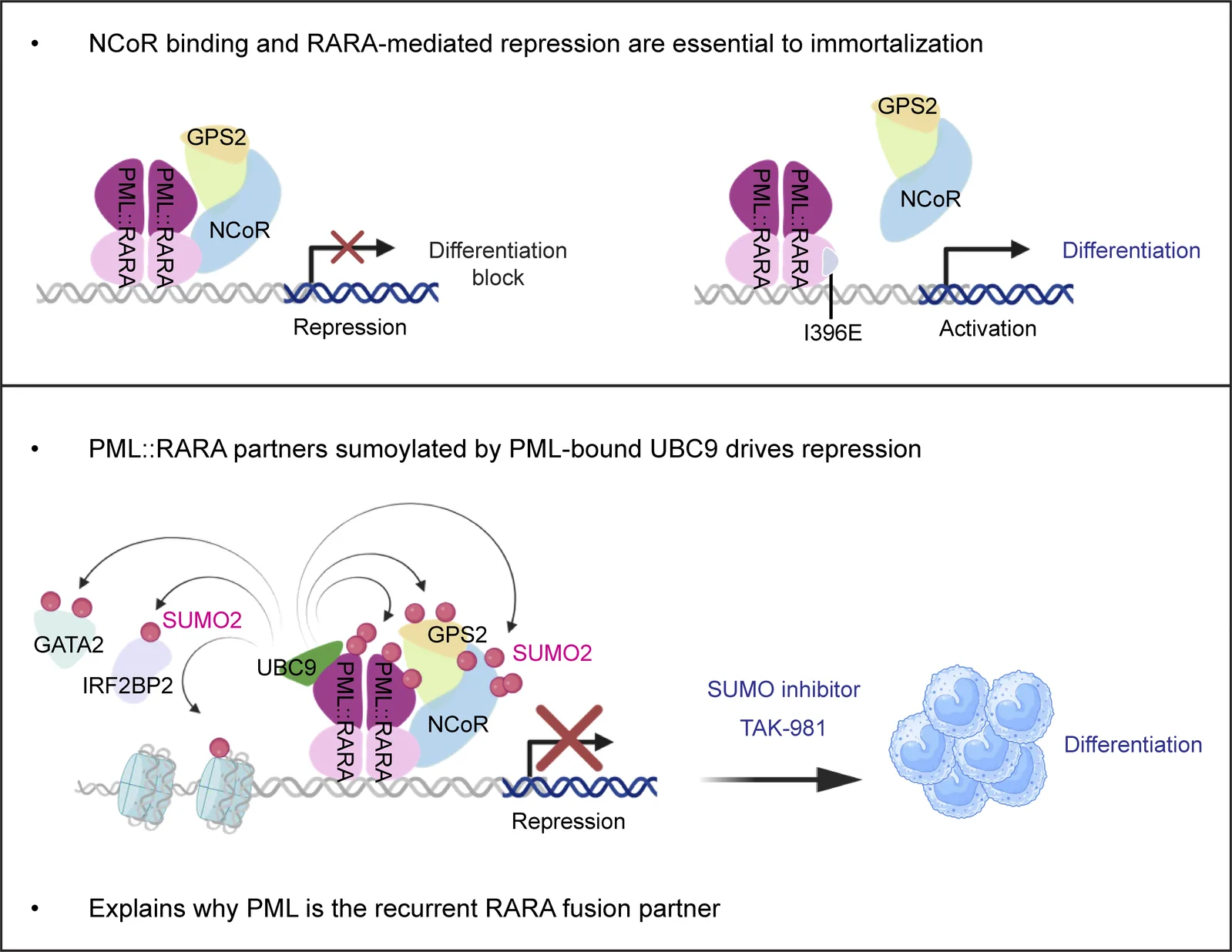

## Introduction

Acute promyelocytic leukemia (APL) is driven by fusion proteins always involving one of the three retinoic acid (RA) receptors (Geoffroy et al., 2021; Zhang and Qiu, 2025), pointing to the critical role of deregulated RA signaling in initiation of the disease. In most patients, these fusions involve RARA and PML (de Thé et al., 1990a). While the pathways involved in RA and arsenic trioxide (ATO) triggered PML::RARA degradation and APL cure have been largely deciphered (de Thé et al., 2017; Dos Santos et al., 2013; Rerolle et al., 2024), why RARA and PML are the preferred fusion partners driving APL initiation remains poorly understood. RARA binds corepressors more avidly than other RARs, and repression by RA receptors plays an important role in development (Weston et al., 2003). PML::RARA disrupts both PML nuclear body formation and nuclear receptor–mediated transcriptional control (Nasr et al., 2009). Dominant-negative RARA mutants exert transforming activities in a variety of biological systems, including some breast cancers (Saitou et al., 1995; Tan et al., 2015; Tsai et al., 1992). PML::RARA is an even more potent repressor than RARA (de Thé et al., 1991). Yet, the molecular bases for PML::RARA super-repressive activity remain imperfectly understood. PML-facilitated PML::RARA homodimer formation was proposed to enhance HDAC and corepressor binding (Lin and Evans, 2000), while tethering of PML-bound repressors such as DAXX (Zhu et al., 2005) may contribute to PML::RARA-driven repression. Artificial RARA homodimers inefficiently initiate APL, even in *Pml* null cells, which implies that PML fusion to RARA entails an unidentified gain of function central to leukemogenesis (Licht, 2006; Sternsdorf et al., 2006; Voisset et al., 2018).

Sumoylation is a reversible posttranslational modification implicated in stress responses. Sumoylation is mediated by UBC9, the universal SUMO E2 ligase, in an enzymatic cascade similar to ubiquitination (Celen and Sahin, 2020). In the context of chromatin, sumoylation is associated with transcriptional repression (Tharuka et al., 2025). PML is a massively sumoylated protein, which serves as a scaffold to drive SUMO2 conjugation of a variety of nuclear body–associated partner proteins, particularly upon stress (Sahin et al., 2014; Tessier et al., 2022). SUMO2 conjugation was also linked to stress-induced target catabolism, as discovered by studying ATO-induced PML::RARA degradation (Jaffray et al., 2023; Jaffray et al., 2025; Lallemand-Breitenbach et al., 2001; Lallemand-Breitenbach, 2008). Within regulatory complexes, sumoylation often occurs on multiple members favoring complex stability and regulating their functions (Psakhye and Jentsch, 2012). For example, the nuclear corepressor complex, assembled by NCoR1/2, may undergo SUMO-facilitated nuclear receptor association and/or transcriptional repression (Tiefenbach et al., 2006). Here, we demonstrate that fusion of PML to RARA drives sumoylation of RARA-associated corepressors and other key partners to promote transcriptional repression and progenitor immortalization.

## Results and discussion

### A key role of repression in hematologic progenitor immortalization by PML::RARA

**Figure 1.**
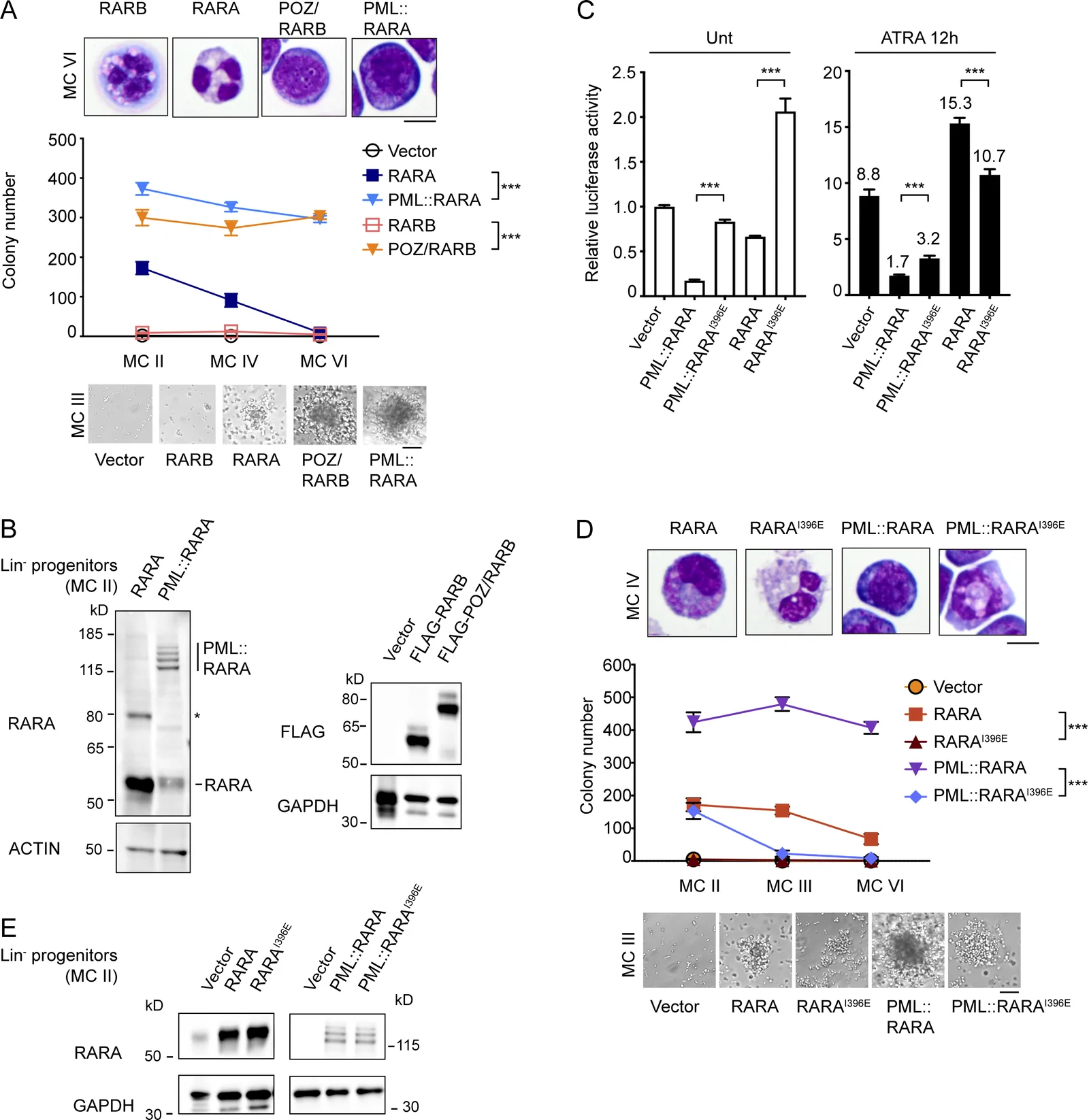
**Transcriptional repression is critical for RARA and PML::RARA-driven progenitor immortalization. (A)** Primary murine Lin^−^ hematopoietic progenitors were retrovirally transduced with RARA, RARB, or their fusions and cultured in methylcellulose with cytokines. MGG staining at the sixth passage (MC VI, upper panel) illustrates the morphology of transformed cells; colony numbers were quantified at the indicated replating (middle panel), and representative colony morphology at the third plating (MC III, lower panel) is shown. Scale bars, 10 μm (upper panel) and 100 μm (lower panel). *N* = 3. **(B)** Western blot analysis of RARA, PML::RARA (left), RARB, and POZ-RARB (right) expression in Lin^−^ progenitors at the second passage (MC II). The asterisk indicates an unidentified posttranslational modification of RARA. *N* = 2. **(C)** RARE/DR5 luciferase reporter assay in 293T cells transfected with RARA, PML::RARA, their I396E mutants that are deficient for NCoR binding. Cells were treated for 12 h with ATRA (1 μM) or left untreated (Unt). Change fold of ATRA treatment over vector without treatment was indicated on the top of each bar in the ATRA panel. *N* = 2. **(D)** MGG staining at the fourth passage (upper panel), colony counts (middle panel) at the indicated replating, and representative morphology at the third plating (lower panel) of primary mouse Lin^−^ hematopoietic progenitors transformed with RARA fusions or their I398E mutants. *N* = 3. Scale bar, 10 mm (upper panel) and Scale bar, 100 mm (lower panel). **(E)** Western blot of RARA, PML::RARA, and their I396E mutants from Lin^−^ progenitors at the second passage. *N* = 2. **(A and D)** Cells were replated every 5–7 days; MC II, III, IV, and VI denote the second, third, fourth, and sixth methylcellulose replating. **(A, C, and D)** Data are presented as the mean ± SD of three technical triplicates from one representative experiment out of at least two biologically independent experiments. Statistical significance was assessed by two-way ANOVA (A and D) or two-tailed unpaired *t* test (C), ***P < 0.001. Source data are available for this figure: SourceData F1.

In primary murine hematopoietic progenitors, PML::RARA expression results in their enhanced self-renewal and differentiation block, as does overexpression of RARA, but not RARB or RARG (Fig. 1 A) (Du et al., 1999; Zhu et al., 2005). Yet, only PML::RARA ensures long-term progenitor immortalization after the fifth replating, contrasting with RARA (Fig. 1 A). Interestingly, the levels of RARA protein expression were consistently much higher than those of PML::RARA at the second passage (Fig. 1 B), suggesting that the higher RARA expression levels in immortalized progenitors compensate for its lower intrinsic repressive activity. Importantly, fusion of the POZ repression domains of PLZF onto RARB allowed efficient progenitor immortalization *ex vivo* (Fig. 1 A), highlighting the need of a strong basal transcriptional repression of RAR target genes in this process.

In RA absence, PML::RARA or RARA potently binds the NCoR1/2 complexes, and RARA I396 residue is required for this binding (le Maire et al., 2010). Importantly, PML::RARA^I396E^ or RARA^I396E^ failed to efficiently repress basal expression of RA-sensitive reporters and no longer immortalized primary progenitors (Fig. 1, C–E). Collectively, repression of RA receptor target genes by NCoR-bound PML::RARA or RARA is required for primary hematopoietic progenitor immortalization, but can be mimicked by fusion of a strong repressive domain onto another RA receptor.

### Sumoylation of PML::RARA partners tightly correlates with transformation

**Figure S1.**
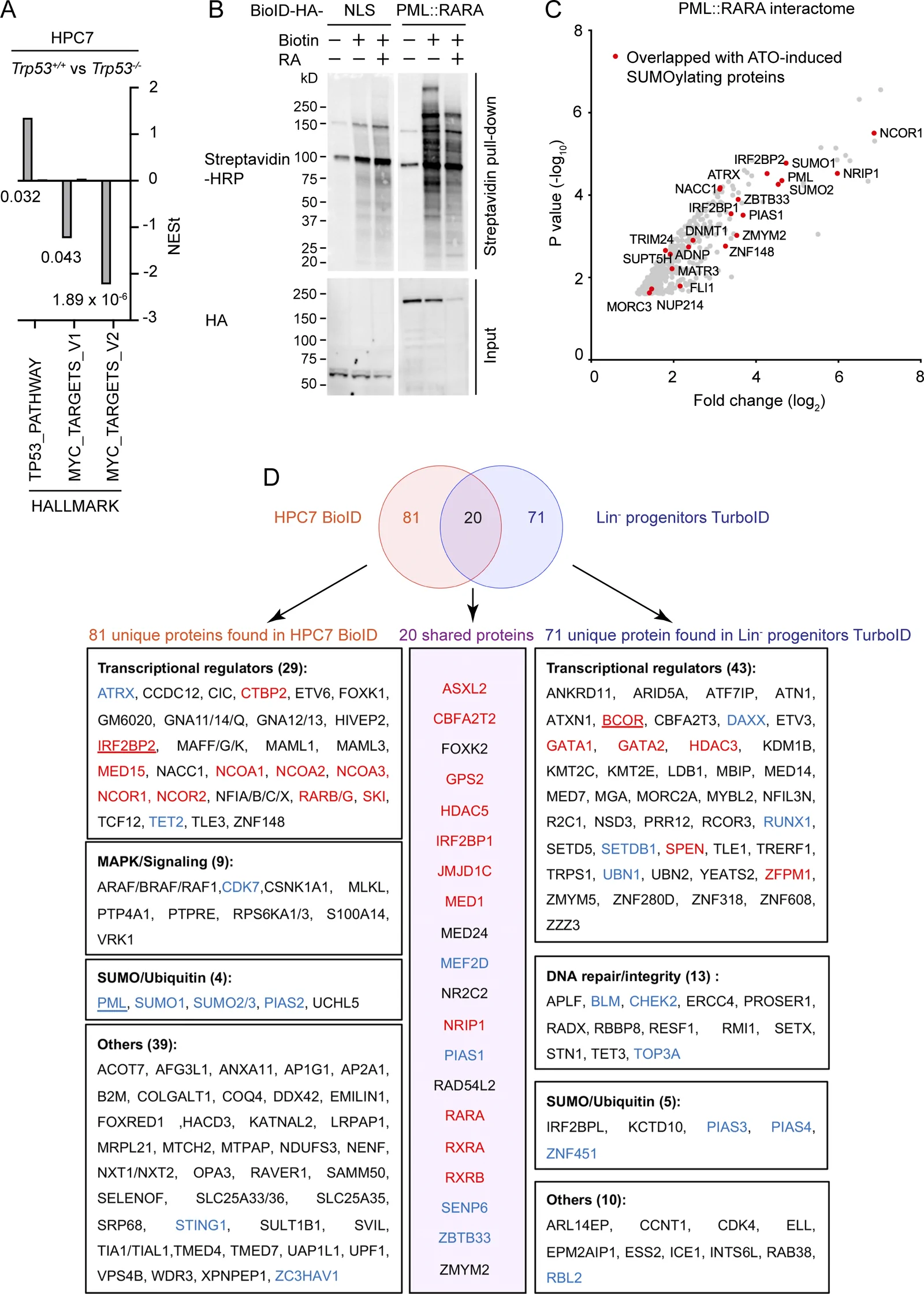
**PML::RARA-interacting proteins. (A)** GSEA comparing parental and *Trp53*^*−/−*^ HPC7 cells; P value was indicated on the bottom of each bar. **(B)** Western blot analysis of lysate- and streptavidin-purified fractions from HPC cell lines expressing NLS-BioID or PML::RARA-BioID with or without 24-h ATRA treatment. *N* = 2. **(C)** Fold change and statistical significance of PML::RARA interactors in HPC7 cells (this study). Overlap with ATO (1 h)-induced sumoylated proteins previously identified in primary mouse APL blasts is indicated by red dots. **(D)** Comparison of PML::RARA interactants identified in HPC7 BioID and Lin^−^ progenitor TurboID (Katerndahl et al., 2024). The protein list here was generated by the cutoff at log_2_ fold change >2.5 and FDR <0.05 in Lin^−^ progenitor TurboID or FDR <0.01 in HPC7 BioID. The proteins labeled in blue and red are known direct/indirect regulators of PML and RARA. RARA fusion partners are underlined. Source data are available for this figure: SourceData FS1.

**Figure 2.**
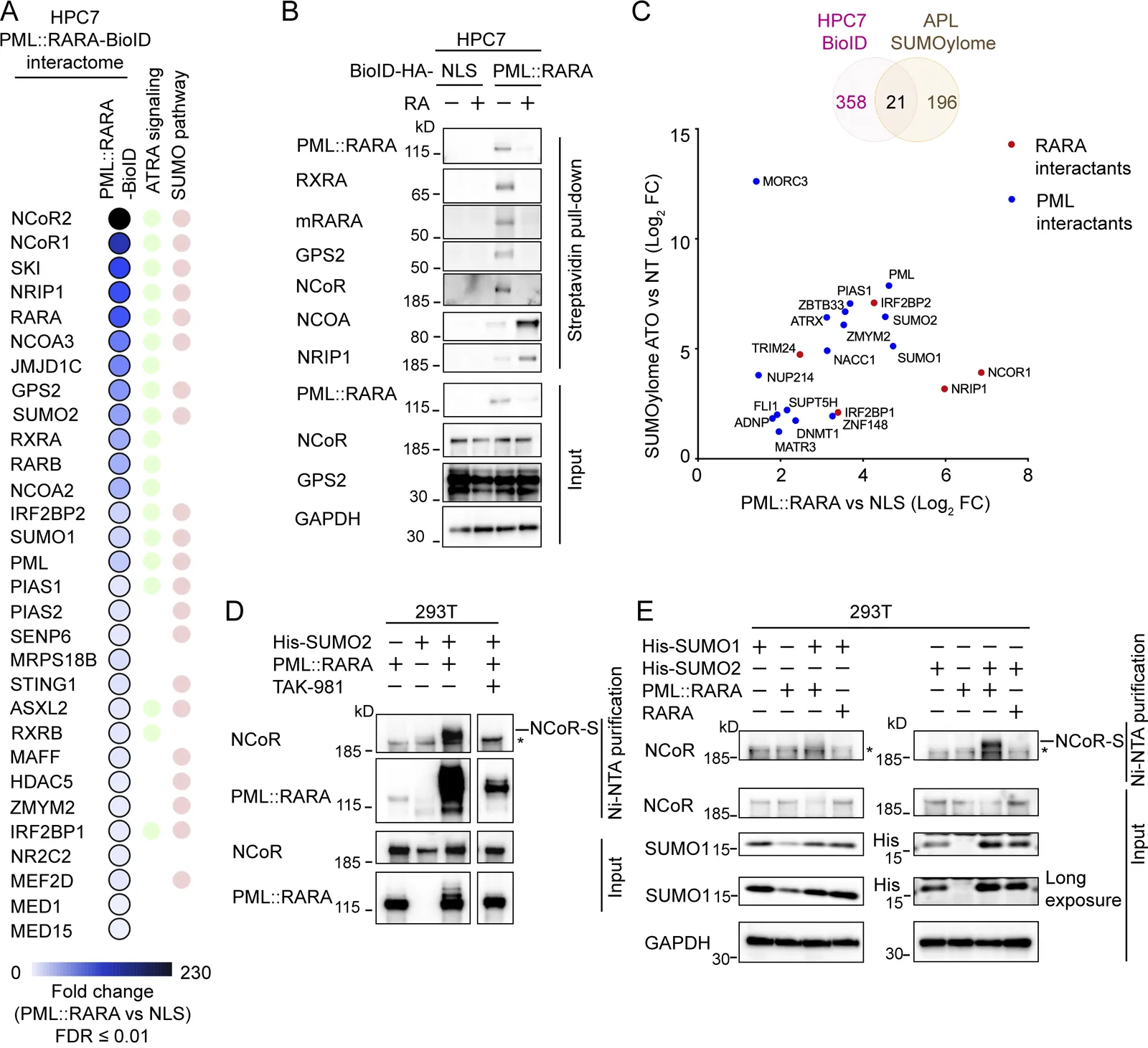
**PML::RARA partners undergo SUMO2 conjugation. (A)** Dot plot of the top 30 proteins that showed an increase in interaction with PML::RARA, ranked by fold enrichment over NLS-BioID control. *N* = 2 for NLS-BioID, *N* = 3 for PML::RARA-BioID. Proteins annotated in green are classified as ATRA signaling (reported to participate in RA/ATRA signaling based on published literature), and those annotated in pink are “SUMO pathway” proteins (previously implicated in the SUMO pathway or identified as SUMO conjugates). **(B)** Western blot validation of selected PML::RARA interactors after biotin labeling followed by streptavidin pulldown. Cells were pretreated with or without RA (1 μM) for 2 h, followed by biotin (50 μM) addition, with RA treatment continued for a total of 18 h. *N* = 3. **(C)** Top: Venn diagram depicting 21 PML::RARA partner proteins from the HPC7 BioID that were previously found in ATO-responsive SUMOylome from primary mouse APL blasts (Tessier et al., 2022). Bottom: Diagram showing the comparative enrichment of PML::RARA interactants (versus NLS) and ATO-enhanced sumoylation for these 21 proteins. RARA- and PML-binding proteins are highlighted in red and blue, respectively. **(D and E)** Western blot analysis of lysates and Ni-NTA–purified fractions from 293T cells transfected with the indicated constructs. *N* = 2. In D, 293 T cells were treated with SUMO inhibitor TAK-981 (100 nM) for 4 h on day 2 after transfection before analysis. NcoR-S: SUMO2-conjugated NCoR. Asterisk denotes remaining unsumoylated NCoR. Source data are available for this figure: SourceData F2.

To identify partners involved in enhanced repression by PML::RARA, we expressed a DHFR-PML::RARA-BioID (thereafter indicated PML::RARA-BioID) fusion in mouse *Trp53*^*−/−*^ HPC-7 stem/progenitor cells, which exhibit very similar features to their parental cell (except for downregulation of basal Trp53 signaling and activation of Myc targets, as expected, Fig. S1 A), but can tolerate stable PML::RARA expression (Ferrucci et al., 1997). Comparison between DHFR-NLS-BioID and PML::RARA-BioID interactants identified 379 specific interactors (Fig. 2 A, Fig. S1 B, and Table S1). 20 of them were recently identified at high confidence (log_2_ fold change >2.5) using a similar approach in different cellular systems (Katerndahl et al., 2024). A detailed comparison between PML::RARA interactors in these two systems is presented in Fig. S1 D. The greatest enrichments were observed for RARA interactors with repressive abilities, including NCoR1/2, NRIP, GPS2, HDACs, SUMO2 (Fig. 2 A), although some transcriptional activators (NCOA2/3) were also identified (Fig. 2 A). Unexpectedly, we identified mRARA/B/G, which are not considered to be direct RARA interactors. PML binders (SUMO1/2, PIAS, TET2) were also efficiently purified. In biochemical validation experiments, RA led to the expected dissociation of NCoR or GPS2, but greatly enhanced interactions with NCOA and NRIP1 (Fig. 2 B).

A significant number of these PML::RARA-interacting proteins were recently identified by SUMO2 proteomics in ATO-treated APL cells *in vivo*, notably NCoR1/2 and NRIP1 (Fig. 2 C and Fig. S1 C) (Tessier et al., 2022). Importantly, in transfected 293T cells, PML::RARA, but not RARA, allowed NCoR conjugation by SUMO2 (reversed by the SUMO inhibitor, TAK-981 [Lightcap et al., 2021]) (Fig. 2 D), even in ATO absence, while interestingly, this was not the case for SUMO1 (Fig. 2 E). Thus, similar to PML partners (Sahin et al., 2014), RARA-interacting proteins may also undergo SUMO2 conjugation in the presence of PML::RARA, most likely through PML-mediated tethering of UBC9.

**Figure 3.**
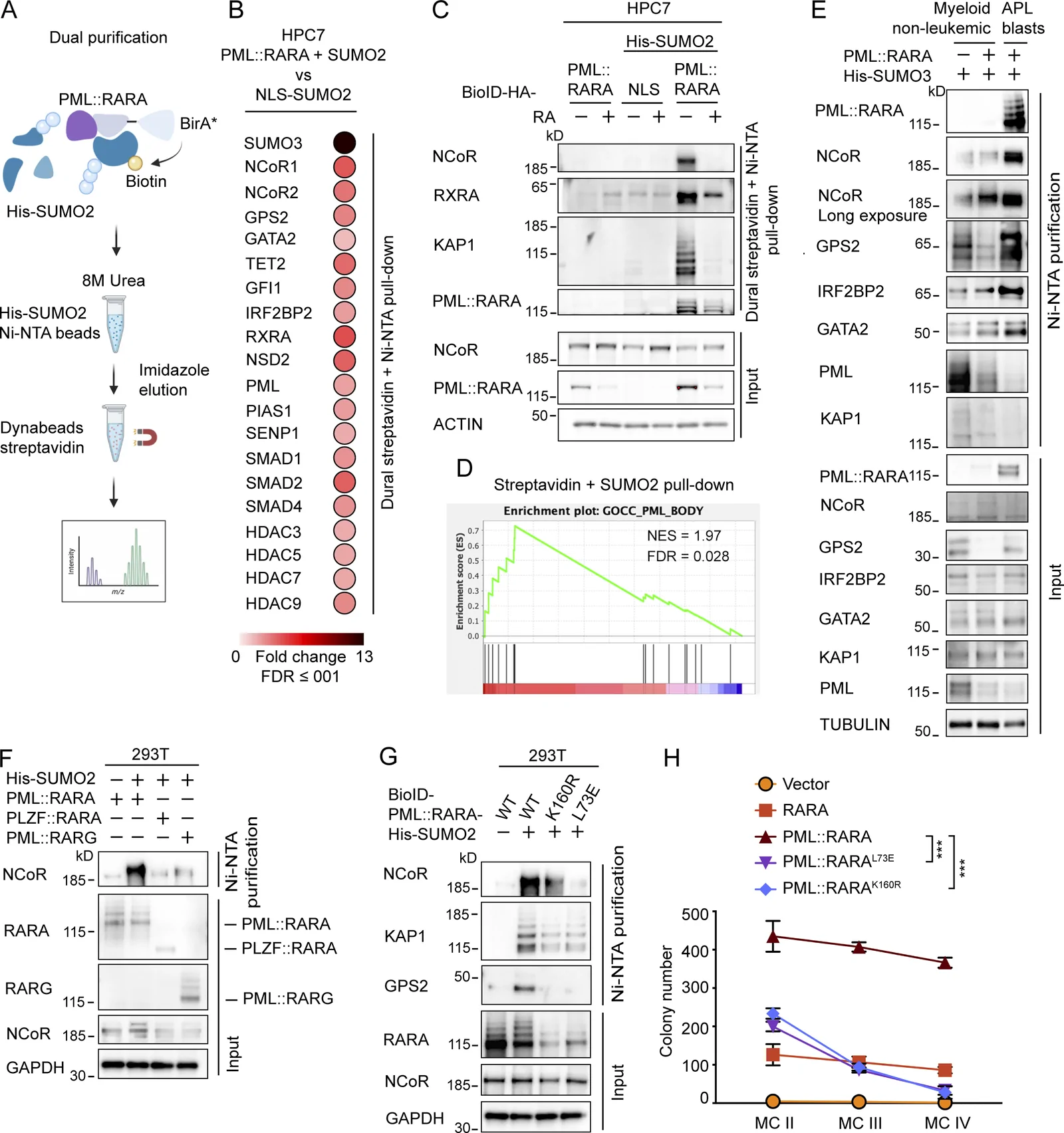
**Fusion of PML to RARA promotes SUMO2 conjugation of RARA-associated partners. (A)** Experimental scheme of Ni-NTA plus streptavidin double-purification strategy used for mass spectrometry analysis of HPC7 cells expressing His_10_-SUMO2 together with NLS-BioID or PML::RARA-BioID. **(B)** Dot plot of the top 20 PML::RARA-BioID (*N* = 4) interactors compared to NLS-BioID (*N* = 4), with significant SUMO2 conjugation in His_10_-SUMO_2_–expressing HPC7 cells. **(C)** Western blot validation of lysates and Ni-NTA– plus streptavidin-purified fractions from HPC7-expressing PML::RARA-BioID alone, or co-expressing His_10_-SUMO2 with NLS-BioID or PML::RARA-BioID. Cells were pretreated with or without RA (1 μM) for 2 h, followed by biotin (50 μM) addition, with RA treatment continued for a total of 18 h. *N* = 3. **(D)** GSEA of proteomics data from His_10_-SUMO2 HPC7 cells expressing PML::RARA-BioID versus NLS-BioID reveals PML_BODY as the sole significantly enriched pathway. **(E)** Western blot analysis of His_10_-SUMO_3_ knock-in mice expressing PML::RARA at nonleukemic stage (*N* = 4) or full-blown APL blasts (*N* = 6) following Ni-NTA purification. **(F)** Western blot analysis (NCoR antibody) of lysates and Ni-NTA–purified fractions from 293T cells transfected with His_10_-SUMO2 and indicated RAR fusion constructs. *N* = 2. **(G)** Western blot analysis of lysates and Ni-NTA– plus streptavidin double-purified fractions from 293T cells transfected with His_10_-SUMO2 and the indicated PML::RARA mutants. *N* = 2. **(H)** Colony counts of Lin^−^ progenitors transformed with RARA and PML::RARA mutants at the indicated replating. Cells were replated every 5–7 days; MC II, III, and IV denote the second, third, and fourth methylcellulose replating. *N* = 2. Data are presented as the mean ± SD of three technical triplicates from one representative experiment out of at least two biologically independent experiments. Statistical significance was assessed by two-way ANOVA, ***P < 0.001. Source data are available for this figure: SourceData F3.

To validate that other PML::RARA interactants may undergo basal PML::RARA-driven SUMO2 conjugation, we then expressed PML::RARA-BioID and His_10_-SUMO2 in HPC7 cells and performed a stringent dual streptavidin plus nickel–nitrilotriacetic acid (Ni-NTA) purification to identify SUMO2-conjugated PML::RARA interactants by mass spectrometry (Fig. 3 A). NCoR1/2 were again the top hits, together with other RARA-associated master regulators including RXRA, GPS2, GATA2, IRF2BP2, NSD2, and HDAC3/5/7/9 (Fig. 3, B and C) (Andrade et al., 2016; Barysch et al., 2021; Chun et al., 2003), while other sumoylated interactors were associated with PML nuclear bodies (TET2, PIAS1, KAP1) (Fig. 3 D). To demonstrate that PML::RARA may favor basal sumoylation of RARA-associated partners in a physiological setting, we used progenitors derived from His_10_-SUMO_3_ knock-in mice expressing, or not, PML::RARA at the preleukemic stage (where PML::RARA expression is barely detectable) or after evolution to full-blown APL (where PML::RARA becomes abundantly expressed [Westervelt et al., 2003]). Only primary leukemic cells showed highly enhanced basal SUMO2 conjugation of PML::RARA-associated proteins, particularly NCoR and its associated proteins such as GPS2, as well as GATA2 or IRF2BP2 (Fig. 3 E). Basal levels and/or sumoylation of PML or PML-associated proteins were decreased, possibly reflecting the distinct differentiation status of those cells.

Among APL-associated fusion proteins, NCoR sumoylation was only observed upon PML::RARA, but not PLZF::RARA or PML::RARG expression (Fig. 3 F), in keeping with the fact that PLZF does not bind UBC9, while RARG does not efficiently recruit NCoR (Farboud et al., 2003). Moreover, PML::RARA point mutants impaired in their UBC9 recruitment ability (L73E or K160R) (Bregnard et al., 2022; Wang et al., 2018; Zhu et al., 2005) yielded a significant decrease in SUMO2 conjugation of RARA-bound NCoR or GPS2 (Fig. 3 G). These mutants are also defective for PML::RARA-driven *ex vivo* immortalization (Fig. 3 H) and *in vivo* transformation (Wang et al., 2018; Zhu et al., 2005), tightly correlating efficient NCoR1/2 sumoylation to *PML::RARA*-driven transformation.

Collectively, the fusion of PML to RARA allows efficient SUMO2 conjugation of multiple RARA partners, the NCoR complex, but also key other transcriptional regulators such as GATA2 and IRF2BP2, potentially modulating their transcriptional output (Chen et al., 2018; Tiefenbach et al., 2006; Wang et al., 2020) and contributing to repression-mediated immortalization.

### Fusion of UBC9 to RARA allows long-term progenitor immortalization

**Figure 4.**
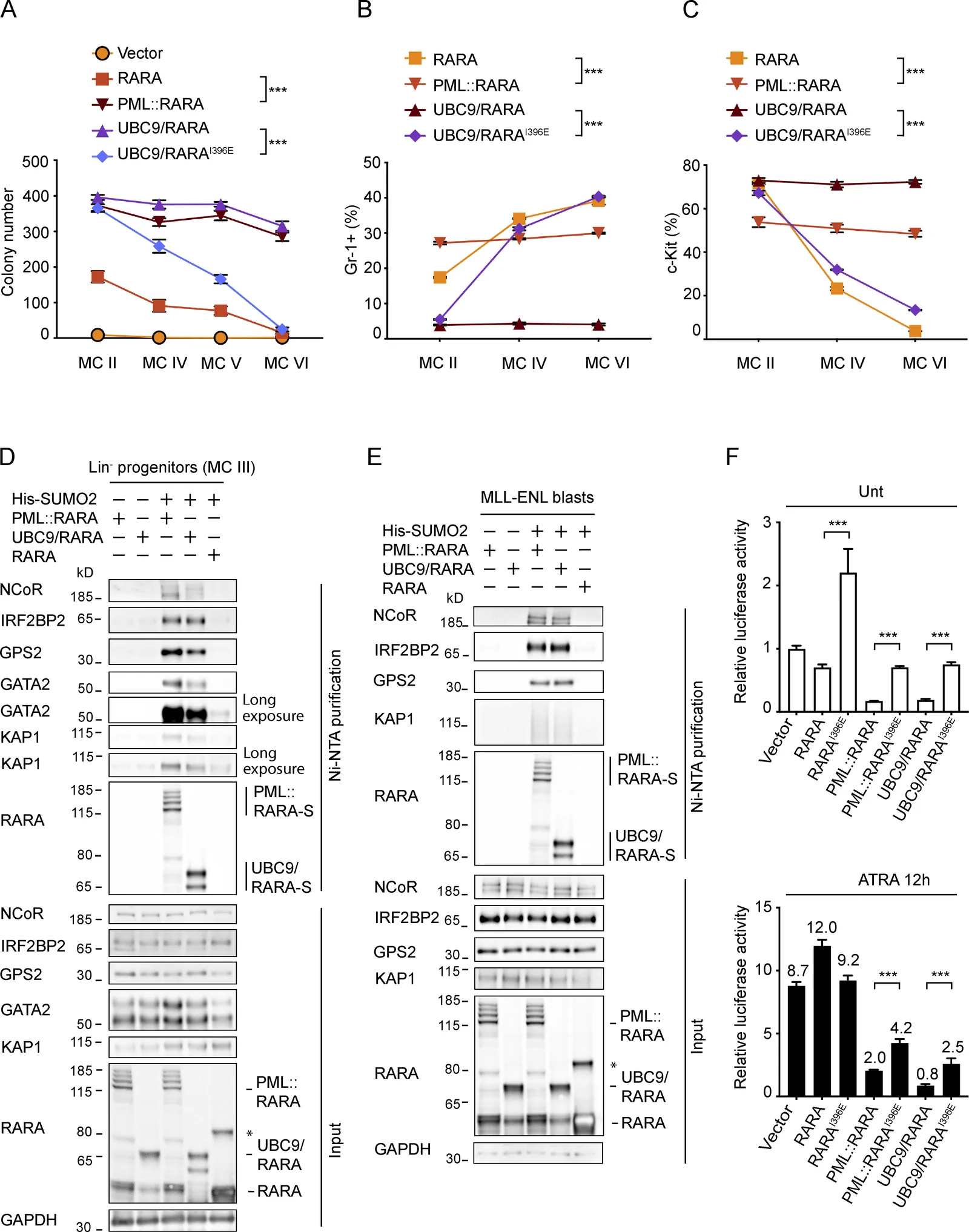
**Fusion of UBC9 to RARA enforces sumoylation of RARA-bound partners and allows long-term progenitor immortalization. (A)** Colony counts from serial replating assays of primary mouse Lin^−^ hematopoietic progenitors transformed with UBC9/RARA, RARA fusions, or their I396E mutants defective for NCoR binding. *N* = 3. **(B and C)** FACS analysis of differentiation marker Gr-1 (B) or stemness marker c-Kit (C) expression in Lin^−^ progenitors transformed with indicated fusions at the specified replating. *N* = 2. **(D and E)** Western blot analysis of lysates and Ni-NTA–purified His-tagged SUMO conjugates from Lin^−^ progenitors (D, *N* = 3) or primary MLL-ENL blasts transduced with RARA fusions (E, *N* = 2). PML::RARA-S or UBC9-RARA-S denotes SUMO-conjugated PML::RARA or UBC9/RARA. The asterisk indicates an unidentified posttranslational modification of RARA. **(F)** RARE/DR5 luciferase reporter assay showing transcriptional activity of RARA, PML::RARA, and their mutants after 12-h ATRA (1 μM) or vehicle (untreated, “Unt”) treatment (*N* = 2). **(A–C)** Cells were replated every 5–7 days; MC II, IV, V, and VI denote the second, fourth, fifth, and sixth methylcellulose replating. **(A–C and F)** Data are presented as the mean ± SD of three technical triplicates from one representative experiment out of at least two biologically independent experiments. Statistical significance was assessed by two-way ANOVA (A–C) or two-tailed unpaired *t* test (F), ***P < 0.001. Source data are available for this figure: SourceData F4.

**Figure S2.**
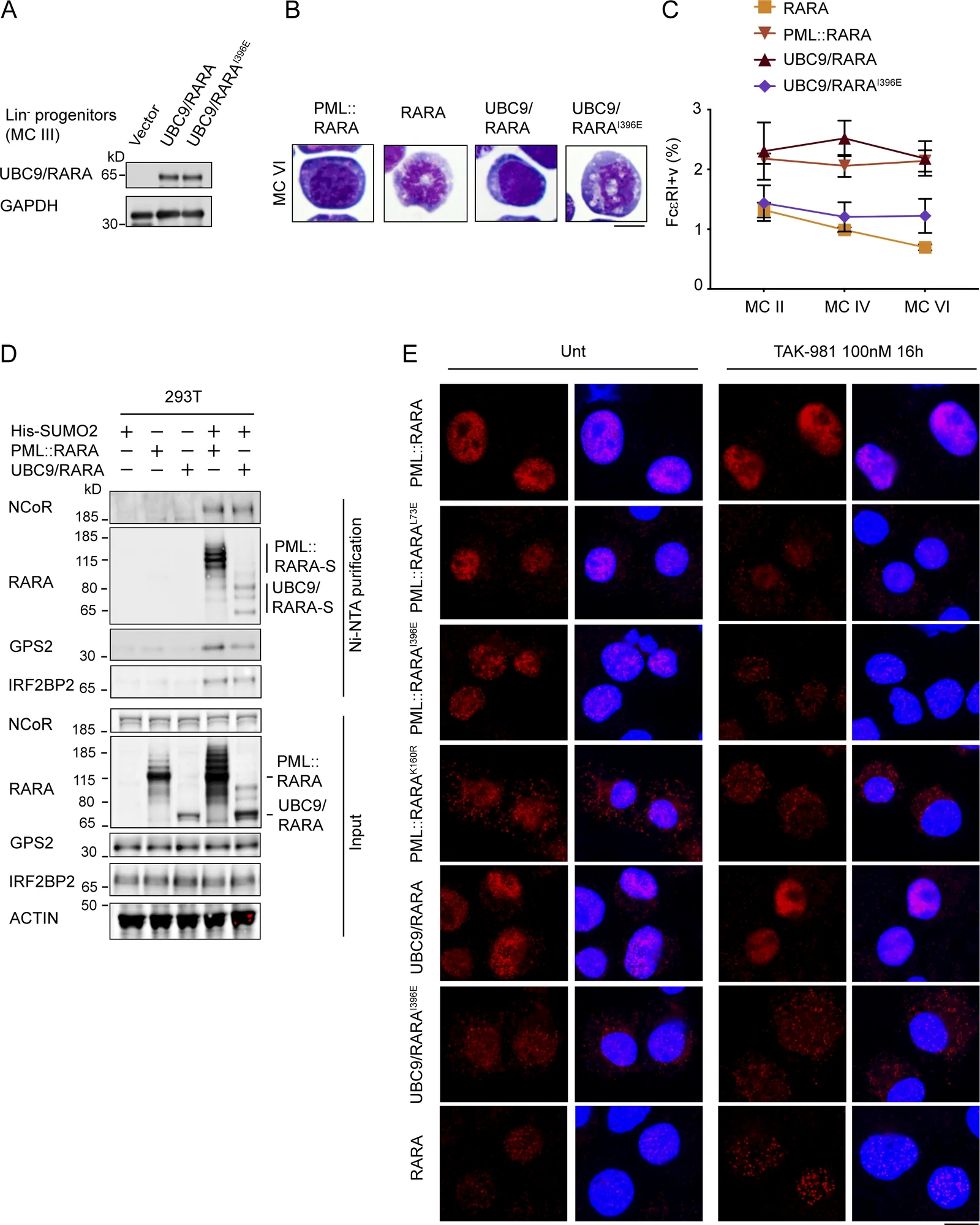
**Exploration of UBC9/RARA and its I396E mutant. (A)** Western blot analysis of UBC9/RARA and UBC9-RARA^I396E^ expression in Lin^−^ progenitors at the third methylcellulose passage (MC III). Representative cells for *N* = 3. **(B)** MGG staining of primary mouse Lin^−^ hematopoietic progenitors transformed with UBC9/RARA, RARA fusions at the sixth passage (MC VI). Scale bar, 10 μm. *N* = 3. **(C)** FACS analysis of mast cell marker FcεRI expression in Lin^−^ progenitors transformed with indicated fusions, at the specified replating. *N* = 2. **(D)** Western blot analysis of lysates and Ni-NTA–purified His-tagged SUMO conjugates from Lin^−^ progenitors transduced with indicated constructs. *N* = 2. PML::RARA-S or UBC9-RARA-S denotes SUMO-conjugated PML::RARA or UBC9/RARA. **(E)** TAK-981 does not alter the cellular localization of RARA fusions. Confocal analysis of subcellular localization of RARA, PML::RARA, UBC9/RARA, and their mutants in Lin^−^ progenitors after 16 h of TAK-981 (100 nM) treatment in methylcellulose, detected with an anti-hRARA antibody. Representative cells for *N* = 2. Scale bar, 10 mm. Source data are available for this figure: SourceData FS2.

If a major role of PML is to recruit UBC9 and enforce sumoylation of RARA-associated repressors, UBC9/RARA should efficiently immortalize progenitors. Indeed, UBC9/RARA fusions, but not overexpressed RARA or UBC9/RARA^I396E^, allowed long-term immortalization (Fig. 4, A–C; and Fig. S2, A–C). Remarkably, UBC9/RARA fusion boosts self, NCoR, GATA2, IRF2BP2, GPS2, and RARA partner sumoylation in both 293T transfectants, transduced MLL-driven AMLs, and Lin^−^ progenitors stably expressing His_10_-SUMO2 (Fig. 4, D and E; and Fig. S2 D), and drives potent transcriptional repression (Fig. 4 F). Similar to PML::RARA, only very low levels of UBC9/RARA suffice to confer long-term replating ability (Fig. 4 E). Actually, UBC9/RARA was a more potent repressor than PML::RARA and abolished basal progenitor differentiation (Fig. 4, B, C, and F). UBC9/RARA^I396E^ still exerted some repressive activities and promoted self-renewal, sharply contrasting with RARA^I396E^, implying that other chromatin-associated SUMO2 targets than NCoR contribute to repression (Theurillat et al., 2020). Collectively, anchoring of UBC9 onto RAR target genes promotes their repression and drives progenitor immortalization.

### Sumoylation inhibitors drive target gene activation, differentiation, and loss of self-renewal

**Figure 5.**
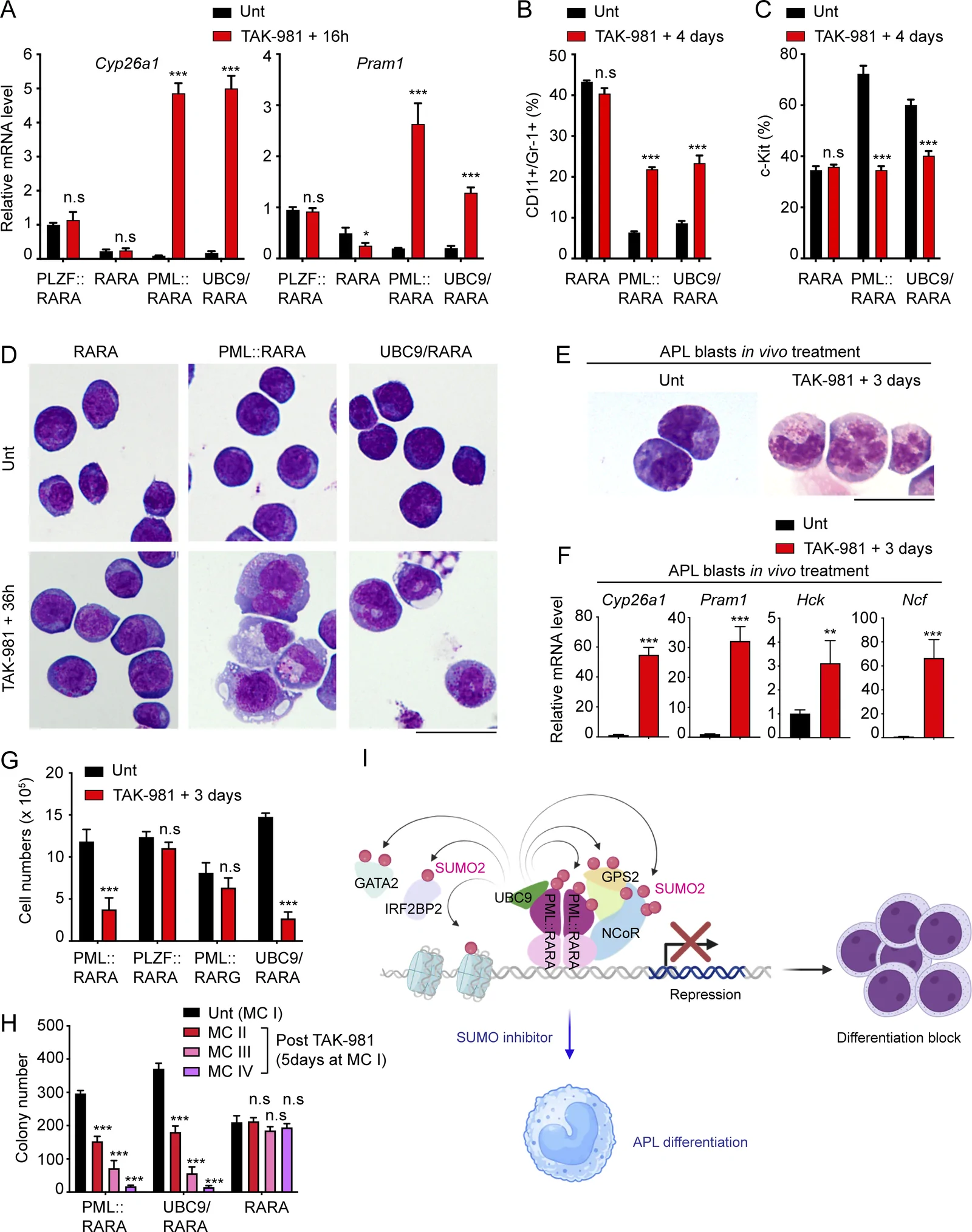
**Inhibition of SUMO conjugation reactivates PML::RARA target genes and induces differentiation. (A)** Real-time PCR for RARA target, *Cyp26a1* and *Pram1*, gene expression in primary progenitors transformed with indicated constructs after TAK-981 (100 nM) treatment for 18 h in methylcellulose. *N* = 3. **(B and C)** FACS analysis of differentiation marker Gr-1 and CD11 (B) or stemness marker c-Kit (C) expression in progenitors transformed with indicated fusions after 4 days of TAK-981 (50 nM) treatment in methylcellulose. *N* = 3. **(D)** MGG staining of progenitors after 36 h of TAK-981 treatment (50 nM) in methylcellulose. **(E)** MGG staining of primary mouse APL blasts from bone marrow after *in vivo* intravenous treatment with TAK-981 (15 mg/kg) for 3 days. *N* = 4. Scale bar, 20 mm. **(F)** Real-time PCR for RARA target *Cyp26a1*, *Pram1*, *Ncf*, and *Hck* gene expression in primary mouse APL blasts isolated from bone marrow after *in vivo* treatment with TAK-981 as in E. **(G)** Abundance of Lin^−^ progenitors expressing the indicated fusions after 5 days of TAK-981 treatment (50 nM) in methylcellulose. *N* = 3. **(H)** Colony counts of primary progenitors transformed with indicated fusions. Cells were first treated with TAK-981 (50 nM) for 5 days in methylcellulose, followed by the subsequent replating without SUMO inhibitor. *N* = 2. Cells were replated every 5–7 days; MC I, II, III, and IV denote the first, second, third, and fourth methylcellulose replatings. **(I)** Schematic model illustrating PML-driven sumoylation of PML::RARA-bound interactors enforcing the differentiation block in APL. **(A–C and F–H)** Data represent the mean ± SD of three technical repeats from one representative experiment out of at least two biologically independent experiments. Statistical significance was assessed by an unpaired *t* test: *P < 0.05, **P < 0.005, and ***P < 0.001. n.s = not significant.

**Figure S3.**
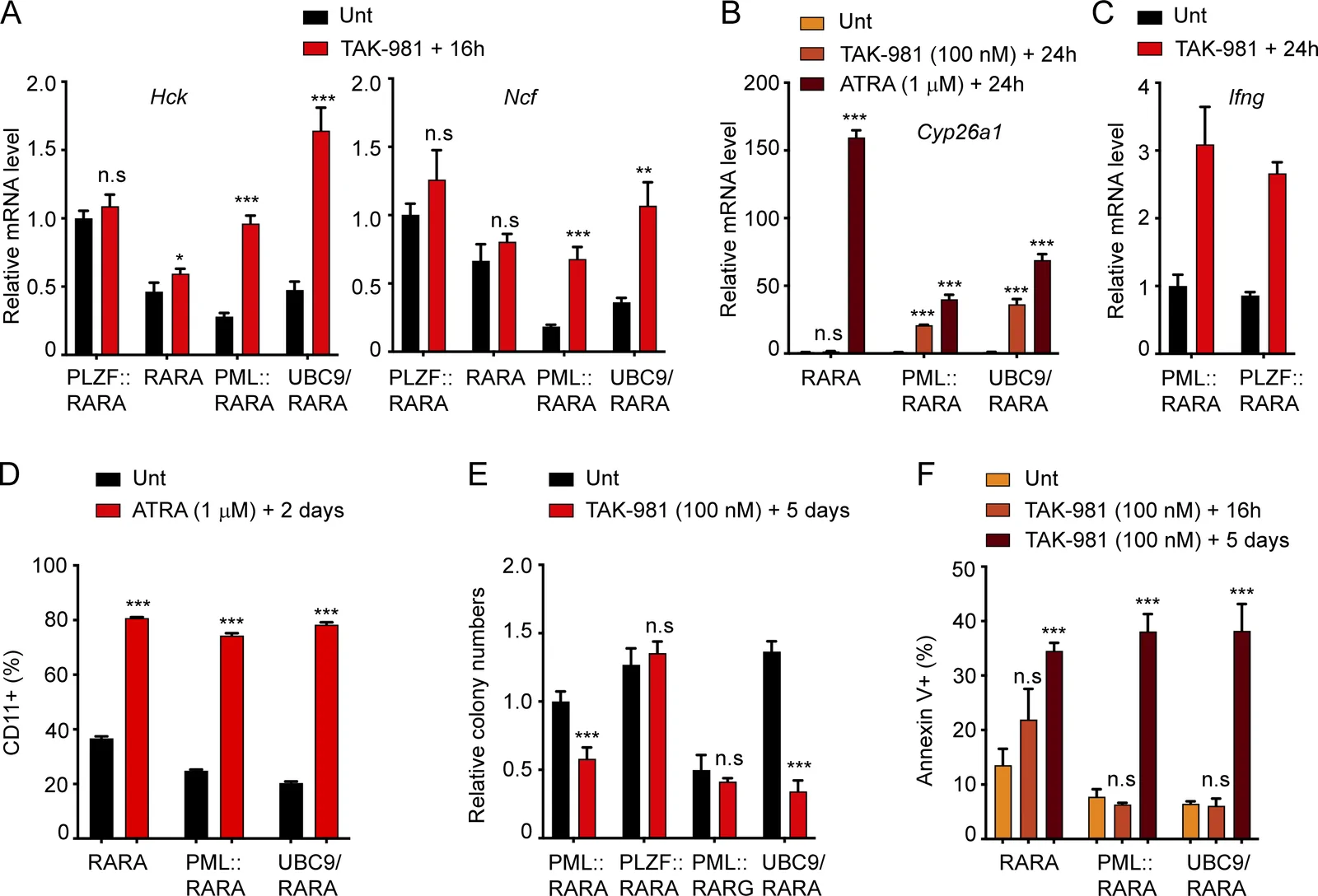
**TAK-981 restores RARA target gene expression, promotes differentiation and apoptosis, and impairs colony formation. (A)** Real-time PCR of RARA target genes *Hck* and *Ncf1* in primary progenitors transformed with indicated constructs after 16 h of TAK-981 treatment (100 nM) in methylcellulose. *N* = 3. **(B and C)** Real-time PCR analysis of *Cyp26a1* (B) and *Ifng* (C) expression in PML::RARA- or PLZF::RARA-transformed progenitors treated with TAK-981 (100 nM, 24 h) in methylcellulose. *N* = 2. In B, progenitors were treated with ATRA (1 μM) for 24 h in methylcellulose. **(D)** FACS analysis of differentiation marker CD11 expression in Lin^−^ progenitors transformed with the indicated fusions and treated with ATRA (1 μM) for 2 days. *N* = 2. **(E)** Colony counts of primary mouse Lin^−^ progenitors transformed with PML::RARA, PLZF::RARA, PML::RARG, or UBC9/RARA. *N* = 3. **(F)** FACS analysis of annexin V staining of RARA-, PML::RARA-, and UBC9/RARA-transformed progenitors treated with TAK-981 (100 nM) for 16 h or 5 days. Data represent the mean ± SD of three technical repeats from one representative experiment out of at least two biologically independent experiments. Statistical significance was assessed by an unpaired *t* test: *P < 0.05, **P < 0.005, and ***P < 0.001. n.s = not significant.

If NCoR sumoylation has any significant role in PML::RARA-dependent transcriptional repression, chemical inhibitors of sumoylation should activate PML::RARA or UBC9/RARA targets. TAK-981 treatment did not alter the cellular localization of RARA fusions (Fig. S2 E), but indeed yielded reactivation of canonical RAR targets, although less efficiently than RA (Fig. 5 A and Fig. S3, A and B). As expected, this was found in PML::RARA- or UBC9/RARA-transformed primary progenitors, but not in RARA- or PLZF::RARA-immortalized ones, while TAK-981 activated *Ifng* gene in both settings (Fig. S3 C). Thus, repression driven by PML::RARA or UBC9/RARA fusions directly involves sumoylation.

*Ex vivo* treatment of PML::RARA- or UBC9/RARA-transformed primary progenitors by TAK-981 induced their differentiation (Fig. 5, B–D; and Fig. S3 D). TAK-981-initiated differentiation was also obtained *in vivo*, using murine APL models (Fig. 5 E), and was accompanied by target gene reactivation (Fig. 5 F). In PML::RARA- and UBC9/RARA-transformed cells, this was associated with a significant decrease in the number of clones (Fig. S3 E) and an even sharper reduction in the total number of cells (Fig. 5 G). Subsequent replating of TAK-981–treated MC I progenitors led to a progressive exhaustion of their clonogenic activities, while RARA-transformed progenitors were essentially unaffected (Fig. 5 H). Functionally, a 5-day *ex vivo* treatment drove some apoptosis in all transformed progenitors (Fig. S3 F). Collectively, inhibition of sumoylation in PML- or UBC9/RARA fusions reactivates RAR signaling to drive APL growth arrest and differentiation.

Transcriptional repression of RA target genes was proposed to underlie APL pathogenesis (de Thé et al., 2017), as formally demonstrated here by the *PML::RARA*^*I396E*^ mutant, using progenitor immortalization as a surrogate for transformation. In PLZF::RARA variant APLs, the PLZF POZ domain yields a super-repressive phenotype that entails clinical RA resistance (Licht et al., 1995). Why PML is the recurrent fusion partner and whether this relates to transcriptional repression remained poorly understood (Sternsdorf et al., 2006). We identify a novel mechanism for PML::RARA-driven hyper-repression, through PML-mediated sumoylation of PML::RARA-bound proteins, particularly the NCoR complex, whose stability, nuclear receptor binding, and repressive ability are all tightly regulated by sumoylation (Fig. 5 I) (Hua et al., 2016; Tiefenbach et al., 2006). Other key RARA-associated regulatory proteins (e.g., NCoR1/2, GPS2, GATA2, IRF2BP2, NSD2) (Fig. 3 B) also undergo PML-driven sumoylation, which may impinge on PML::RARA function (Andrade et al., 2016; Barysch et al., 2021; Chun et al., 2003). IRF2BP2 may be a RARA fusion partner in very rare APLs (Shimomura et al., 2016; Yin et al., 2015), while GATA2 forms tight complexes with RARA on DNA (Katerndahl et al., 2024; Tsuzuki et al., 2004). Chromatin sumoylation around PML::RARA binding sites may also contribute to transcriptional repression (Stielow et al., 2008). Domains of PML involved in UBC9 recruitment and sumoylation control (RING, K160) were also required for enhanced partner sumoylation, target gene regulation, TAK-981 sensitivity, progenitor immortalization (Fig. 3 H), and, critically, leukemogenesis *in vivo* (Wang et al., 2018; Zhu et al., 2005). NCoR complex SUMO-interacting motifs may also boost its own binding onto sumoylated PML::RARA, as in other nuclear receptors (Hua et al., 2016; Paakinaho et al., 2021), further contributing to tightly anchor the fusion protein onto this master repressive complex. That UBC9/RARA exerts much stronger transforming effects than RARA and confers phenotypic sensitivity to UBC9 inhibitors strongly argues for a direct functional role of sumoylation of RARA partners and nearby chromatin in transcriptional repression, independently from specific PML interactions such as the DAXX repressor. Finally, we also identified other PML::RARA interactors with potential importance in APL pathogenesis, such as the *Ski* oncogene, which binds to RARA to inhibit RA signaling (Ritter et al., 2006) and confers RA sensitivity to immortalized progenitors (Dahl et al., 1998; Melling et al., 2013).

Previous studies suggested an obligatory role of PML::RARA as an active effector of RA-induced differentiation. Yet, one of the key PML::RARA-interacting proteins is NRIP1, an RA-dependent repressor. Accordingly, in RAR-less MEFs, PML::RARA remains a very poor activator of primary target genes (C. Esnault, unpublished data). TAK-981–induced target gene reactivation (without PML::RARA loss) demonstrates that derepression suffices to initiate the differentiation program *in vivo*, extending our observations that PML::RARA destruction by ATO is enough to initiate APL differentiation. PML fusion to RARA drives dimer-dependent gains of function, including relaxed DNA-binding specificity, enhanced corepressor association (Kamashev et al., 2004; Sternsdorf et al., 2006), or recruitment of PML-bound partners (Occhionorelli et al., 2011; Zhu et al., 2005). Previous studies suggested that these are insufficient to drive immortalization on their own (Sternsdorf et al., 2006; Voisset et al., 2018). The experiments reported here illustrate how critical PML/UBC9-driven posttranslational modifications of RARA partners can contribute to transformation, identifying a key gain of function explaining why PML and RARA are the preferred targets of translocations driving APL. Sumoylation was recently shown to modulate oncogenesis in other settings (Li et al., 2025; Zhang et al., 2025), but also to favor therapeutic response through nuclear receptor activation (Valima et al., 2025), possibly opening novel perspectives for sumoylation inhibitors in cancer therapy.

## Materials and methods

### Cell lines

A mouse HPC7 *Trp53*^*−/−*^ stem/progenitor cell line used in this study was generated from the parental HPC7 cell line (a kind gift from Camille Lobry) by using the Alt-R CRISPR/Cas9 technology (Integrated DNA Technologies [IDT]). Briefly, HPC7 cells were electroporated with guide RNA targeting *Trp53* (5′-GCG​CTG​ACC​CAC​AAC​TGC​AC-3′) and recombinant Cas9 reagent (cat# 1081061; IDT) following the recommendation of the manufacturer. Single-cell cloning was performed into 96 wells, and knockout of the *Trp53* gene was confirmed by sequencing, immunofluorescence, and western blotting analysis (data not shown). *Trp53*^*−/−*^ HPC7 cells were retrovirally transduced with DHFR-PML::RARA-HA-BioID or DHFR-NLS-HA-BioID and selected with puromycin (1 μg/ml) for 3 days and maintained in puromycin-containing culture medium. Puromycin-resistant cells were then retrovirally transduced with His_10_-SUMO2 (IRES-EGFP) and GFP-sorted by FACS. All HPC7 cell lines were maintained in Iscove’s modified Dulbecco’s medium plus GlutaMAX (cat# 31980030; Thermo Fisher Scientific) supplemented with 5% FBS in the presence of 100 U/ml of penicillin, 100 μg/ml of streptomycin, 0.1 mM 2-mercaptoethanol, and 100 ng/ml of soluble Kit ligand recovered from a CHO cell line stably expressing mouse c-Kit. HEK293T cells were maintained in Dulbecco’s modified Eagle’s medium plus GlutaMAX (cat# 41965062; Thermo Fisher Scientific) supplemented with 10% FBS, 100 U/ml of penicillin, and 100 μg/ml of streptomycin.

### Luciferase reporter assay

To assess transcriptional activities of RARA fusion proteins, HEK293T cells were transfected with 200 ng of indicated pSG5 RARA fusion plasmids together with luciferase reporter vectors (200 ng of pDR5-Tk-FLuc and 200 ng of pTk-RLuc for normalization) as previously described (de Thé et al., 1990b). After 24 h, transfected cells were treated with or without all-trans retinoic acid (ATRA) (1 μM) for 12 h. Measurement of firefly and Renilla luciferase activities was then monitored in a total of 36 h after transfection with the dual-luciferase assay system kit (Promega) following the recommendation of the manufacturer.

### Plasmid constructs

All plasmid constructs are listed in Table S2. To generate the pCMV-PML::RARA-BioID2-HA plasmid, an NheI/AgeI fragment containing PML::RARA (obtained by restriction enzyme digestion of pMSCV-PML::RARA) was cloned into the pCMV-BioID2-HA vector (purchased from Addgene). To generate pMSCV-PML::RARA-BioID2-HA, an NheI/MssI fragment from the pCMV-PML::RARA-BioID2-HA plasmid was subcloned into the pMSCV-puro vector. To insert the DHFR fragment and construct pMSCV-DHFR-PML::RARA-BioID2-HA, a synthetic cDNA (GeneArt, Thermo Fisher Scientific) encompassing in-frame *Escherichia coli* DHFR (encoding the R12Y/G67S/Y100I destabilizing domain mutant) in frame with human PML sequence was digested with XhoI/AvrII to obtain a partial DHFR-fused PML fragment containing restriction enzyme site for vector backbone (XhoI) and PML replacement (AvrII, at 285 bp) to be cloned into pMSCV-PML::RARA-BioID2-HA. To generate NLS-BioID2 retroviral vector, a NcoI/BstBI fragment from a synthetic cDNA encoding the SV40 nuclear localization sequence (NLS) was cloned in place of the PML::RARA fragment of the pMSCV-PML::RARA-BioID2-HA plasmid, resulting in a pMSCV-NLS-BioID2-HA construct.

To obtain the pMSCV-RARB plasmid, an EcoRI/BamHI fragment from pSG-RARB (a kind gift from Albane le Maire, CNRS UMR504, INSERM 1054, Université de Montpellier, France) was subcloned into the MSCV-puro vector. For the pMSCV-Flag-HA-POZ-RARB plasmid, nested PCR was used to amplify a Flag-HA tag in frame with POZ domain from MSCV-PLZF-RARA with the following primers: Flag-HA-POZ For: 5′-GCT​GAA​TTC​GCC​ACC​ATG​GAC​TAC​AAG​GAC​GAC​GAT​GAC​AAG​CTC​GAT​GGA​GGA​TAC​CCC​TAC​GAC​GTG​CCC​GAC​TAC​GCC​GAT​CTG​ACA​AAA​ATG​GGC​ATG​ATC​CAG​CTG​CAG​AAC​CCT​AGC​CAC-3′; POZ rev: 5′-GGC​TCT​AGA​CCC​GCC​TCC​ACC​GAT​GGT​CTC​CAG​CAT​CTT​C-3′. The Flag-HA-POZ PCR product, including a glycine–serine linker, was then cloned in-frame with RARB into pMSCV-RARB.

To construct UBC9/RARA expression plasmids, UBC9 was PCR-amplified from the pSG5-Ubc9 plasmid and cloned in-frame with RARA in both pMSCV-RARA and pCMV-RARA vectors using the following primers: UBC9-MSCV-For 5′-TCT​CTC​GAG​GCC​ACC​ATG​TCG​GGG​ATC​GC-3′; UBC9-MSCV-Rev 5′-TTC​GTT​AAC​TCA​CGG​GGA​GTG​GGT​GGC-3′; CMV-UBC9-For 5′-CTG​GCT​AGC​CAC​CAT​GTC​GGG​GAT​CG-3′; CMV-UBC9-Rev 5′-ACC​GGA​TCC​CGG​GGA​GTG​GGT​GGC​C-3′.

RARA fusion point mutants were generated by using QuikChange II XL Site-Directed Mutagenesis Kit (cat# 200522; Agilent Technologies) with the following primers: PML::RARA^L73E^-For 5′-GCC​CGA​AGC​TGC​TGC​CTT​GTG​AGC​ACA​CGC​TGT​GCT​CAG​GAT​GCC​TG-3′, PML::RARA^L73E^-Rev 5′-CAG​GCA​TCC​TGA​GCA​CAG​CGT​GTG​CTC​ACA​AGG​CAG​CAG​CTT​CGG​GC-3′; PML::RARA^K160R^-For 5′-AGG​CAC​ACC​AGT​GGT​TCC​TCC​GGC​ACG​AGG​CCC​GGC​CCC​TAG-3′, PML::RARA^K160R^-Rev 5′-CTA​GGG​GCC​GGG​CCT​CGT​GCC​GGA​GGA​ACC​ACT​GGT​GTG​CCT-3′; RARA^I396E^-For 5′-CCA​AGG​GGG​CTG​AGC​GGG​TGG​AAA​CGC​TGA​AGA​TGG​AGA​TCC​C-3′, RARA^I396E^-Rev 5′-GGG​ATC​TCC​ATC​TTC​AGC​GTT​TCC​ACC​CGC​TCA​GCC​CCC​TTG​G-3′.

### Colony formation assay

For methylcellulose colony formation assay, primary murine Lin^−^ progenitors were obtained from 5-fluorouracil–treated C57BL/6NJ mice (Janvier Labs) using a lineage-positive cell depletion kit (cat# 130-090-858; Miltenyi Biotec). Briefly, bone marrow flushed-out cells were magnetically labeled with a cocktail of biotinylated antibodies against a panel of lineage antigens (CD5, CD45R [B220], CD11B, anti-Gr-1 [Ly-6G/C], 7-4, and Ter-119 antibodies) and anti-biotin microbeads. Lin^−^ progenitors were retrovirally transduced with indicated fusions and maintained in methylcellulose (cat# 3231; StemCell Technology) supplemented with a cocktail of 10 ng/ml of mIL-3, mIL-6, mGM-CSF, and 50 ng/ml of mSCF as previously described (Zhu et al., 2005), and were selected and maintained with puromycin (1 μg/ml) or neomycin (500 μg/ml). To determine the impacts of fusion proteins and TAK-981 (HY-111789; MedChemExpress) on progenitors’ immortalization, 5,000 cells per well in a 6-cell plate were cultured and subsequently replated to assess their clonogenic capacity.

### Biochemistry

To purify PML::RARA interactants or His_10_-SUMO1/2–conjugated proteins, HPC7, Lin^−^ progenitors, and primary MLL-ENL blasts were retrovirally transduced with viruses produced by transient transfections of Plat-E packaging cells (RARA, NLS-BioID, PML::RARA-BioID, and UBC9/RARA) and selected with puromycin (1 μg/ml). GFP-positive cells (transduced with MSCV-IRES-EGFP-His10-SUMO2) were sorted by FACS. In all experiments involving the BioID constructs, cells were treated with trimethoprim (5 μM) to induce NLS and RARA fusion expression. After 24 h, NLS and RARA fusion–expressing cells were treated with biotin (50 μM) for 16 h and lysed by buffer A containing 8 M urea, 50 mM Tris, pH 8, 150 mM NaCl, 10 mM NEM (cat# E3876; Sigma-Aldrich), 15 mM imidazole, (5 U/ml) universal nuclease (cat# 88702; Thermo Fisher Scientific), and proteinase inhibitors (cat# 11 836 170 001; Roche). Protein concentration was determined using Bradford reagent (cat# B6916; Sigma-Aldrich). Equal amounts of protein lysates containing 40 μM of DUB inhibitor PR-619 (cat# 662147; Sigma-Aldrich) were incubated overnight at 4°C with Ni-NTA agarose beads (cat# L30210; Qiagen) for SUMO1/2 conjugates or streptavidin Dynabeads (cat# 65602; Thermo Fisher Scientific) for BioID proteins. The beads were washed six times with buffer A and then analyzed by western blot.

For dual streptavidin plus Ni-NTA purification experiments, His_10_-SUMO2–conjugated proteins were first purified as described above in urea buffer A, then eluted by radioimmunoprecipitation assay (RIPA) buffer containing 250 mM imidazole, 50 mM Tris (pH 8.0), 0.15 M NaCl, 1% NP-40, 1% sodium deoxycholate, 0.1% SDS, proteinase inhibitor, and 40 μM PR-619. Biotinylated and SUMO2ylated proteins were enriched by streptavidin Dynabeads overnight at 4°C. The beads were washed three times with RIPA buffer and then analyzed by western blot.

The following antibodies were used with 1:1,000 dilution for immunodetection in precipitation samples and cell lysates: ACTIN and GAPDH (A2066, G9545; Sigma-Aldrich); RARA and PML (homemade affinity-purified rabbit and mouse); GPS2 and NCoR (PA5-76547, A301-145A; Thermo Fisher Scientific); NRIP1 (NBP3-12264; Novus); RARA, NCOA, RXRA, TUBULIN, GATA2, SUMO1, and SUMO2 (ab27574, ab10491, ab125001, ab4074, ab109241, ab32058, ab81371; Abcam); KAP1 and His tag (4124, 2365; Cell Signaling); IRF2BP2 (18847-1-AP; Proteintech).

### RNA analyses

Total RNA extraction from immortalized progenitors, APL blasts, and HCP7 cells was performed with RNeasy Plus Mini Kit (cat# 74134; Qiagen) and quantified using a NanoDrop One/One (Thermo Fisher Scientific). When required, cDNA was prepared from 1 µg of total RNA with an iScript cDNA Synthesis kit (cat# 1708891; Bio-Rad). Quantitative real-time PCR on cDNA was performed on Bio-Rad CFX thermal cycles with TaqMan gene expression assays from Life Technologies (*Gapdh*, 4352339E; *Cyp26a1*, Mm00514486_m1; *Pram1*, Mm02744730_g1; *Ncf1*, Mm00447921_m1; *Hck*, Mm01241463_m1; *Ifnγ,* Mm01168134_m1). For RNA-sequencing analysis of WT and *Trp53*^*−/−*^ HPC7 cells, libraries were prepared using the Illumina Stranded mRNA Prep kit and paired-end sequenced at 151 bp on an Illumina NextSeq 2000 according to the manufacturer’s instructions. Sequencing reads were pseudoaligned to the mouse mm10 reference genome and used for gene set enrichment analysis (GSEA) using Molecular Signatures Database including H, C2, and C5 gene set collections.

### Flow cytometry analysis

Murine Lin^−^ cell types were analyzed using FACSCanto II (BD). Single-cell suspensions were blocked with Fc block (BD) for 15 min on ice. The following antibodies were used to stain immortalized Lin^−^ progenitor cells (1:1,000): APC-conjugated CD117 (Clone 2B8; eBioscience), APC-Cy7–conjugated anti-CD11b (Clone M1/70, BioLegend), APC-conjugated anti-FcεRI (Clone MAR-1; eBioscience), and phycoerythrin-conjugated anti-Gr1 (Clone RB6-8C5, eBioscience). Staining was performed overnight at 4°C. Cells were washed and resuspended in phosphate-buffered saline (PBS) with paraformaldehyde (PFA) 0.2% before FACS analysis.

### Immunofluorescence analysis

Cytospined Lin^−^ progenitors were fixed with 4% PFA (Sigma-Aldrich) and permeabilized with 0.1% Triton X-100, and then blocked with PBS supplemented with 1% BSA and 0.05% Triton X-100. Cells were incubated with rabbit anti-human RARA antibodies (a kind gift from Scott Kogan) overnight at 4°C and then with the corresponding fluorescence secondary antibodies (Jackson ImmunoResearch or Thermo Fisher Scientific) together with 1 µg/ml of Hoechst 33342 (Thermo Fisher Scientific) for 1 h. Cells were then washed with PBS containing 0.02% Triton X-100 mounted with fluorescence mounting medium (Dako Omnis; cat# S302380) and examined with a LSM 980 confocal microscope equipped with a 63× oil objective lens (AiryScan 2 GaAsP detector; Carl Zeiss Micro-Imaging Inc.).

### Mouse model

The His_10_-HA-SUMO_3_ knock-in strain was generated using the CRISPR/Cas9 system by the i-GONAD electroporation method with the BALB/cByJ strain. BALB/cByJ mice were purchased from Charles River. CRISPR RNA, trans-activating CRISPR RNA, single-stranded DNA, and Cas9 nuclease were purchased from IDT (CRISPR target sequence: 5′-TCA​GTT​TCC​ATT​TCT​TGA​T-3′; ssODN sequence: 5′-CAT​TTC​CCG​CCT​TCA​CAG​ACC​TAA​TAA​GAA​ATG​CAT​CAC​CAT​CAC​CAT​CAT​CAT​CAT​CAT​CAT​TAC​CCA​TAC​GAT​GTT​CCA​GAT​TAC​GCT​GAA​ACT​GAA​CCA​GTT​TCC​GTG​CAG​AAG​GTA​CCT​GCA​CCC-3′). To obtain His_10_-HA-Sumo_3_*PML::RARA* double mutant mice, this strain was crossed with Ctsg-PML::RARA mice on a BALB/cByJ background. For Fig. 3 E, APL blasts were collected from the bone marrow. Nonleukemic myeloid cells were enriched by depletion of lymphoid lineage using CD4/CD8, CD49b, and CD19 microbeads (cat# 130-116-480, 130-052-501, 130-121-301) and MACS column from Miltenyi Biotec.

Leukemic blasts in Fig. 5, E and F were derived from h-MRP8-*PML::RARA* transgenic mice and transplanted by intravenous injection into FVB mice. Serial intravenous transplantations were performed using 10^4^ GFP-sorted APL cells from bone marrow. After 2 wk, mice were intravenously treated with TAK-981 (15 mg/kg) for 3 days and bone marrow APL blasts were analyzed by May–Grünwald–Giemsa (MGG) staining and RT-qPCR. Animals were handled according to the guidelines of institutional animal care committees using protocols approved by the “Comité d’Ethique Experimentation Animal Paris-Nord N. 121” (project no. 58284). Mice were maintained in a 12-h light–dark cycle animal facility under specific pathogen-free conditions with free access to water and food (Institut de Recherche Saint Louis, Paris, France).

### Mass spectrometry analyses

Experiments were performed at the Pitié-Salpêtrière (P3S) Proteomics Core Facility of Sorbonne University. Ni-NTA– or streptavidin-purified proteins were gel-purified and trypsin-digested overnight and processed essentially as described previously (Hospital et al., 2018). Mass spectrometry data acquisition was performed on a NanoElute II UHPLC system coupled to a TIMS-TOF-HT mass spectrometer (Bruker Daltonics), with a nano-electrospray ion source (Bruker CaptiveSpray source). Peptides were injected into a reversed-phase C18 nanocolumn (Aurora 3, 25 cm long, 75 μm internal diameter, 1.7 μm, 120 Å pores, IonOpticks). The mobile phases used were A (0.1% (vol/vol) formic acid in Milli-Q grade water) and B (0.1% (vol/vol) formic acid in acetonitrile). The separation was carried out at 220 nl/min in a 30-min acetonitrile gradient (5–16%/22–25% B). All samples were acquired in a DIA-PASEF acquisition mode, with an *m/z* range 100–1,700 and an ion mobility (IM) range of 0.7–1.3 1/K_0_. Isolation windows were fixed at 35 Da, with a cycle time estimate of 1.17 s. Mass and IM calibration were done linearly using three ions from the Agilent ESI LC/MS tuning mix (*m/z* 622.0290, IM 0.9917 1/K_0_; *m/z* 922.0098, IM 1.1984 1/K_0_; *m/z* 1,221.9906, IM 1.3934 1/K_0_). Mass spectrometry data were analyzed by software Data-Independent Acquisition by Neural Networks, version 1.8.1. Data filtering, statistical, and differential analyses were all carried out with PROSTAR software, version 1.30.7. Data were refined by excluding missing lines and contaminants from the dataset, followed by log_2_ transformation of LFQ intensities. Normalization was carried out by the method of quantile centering, within each condition. Significant differences were evaluated by the Limma test, using an adaptive Benjamini–Hochberg correction. Proteins were deemed significant if P values were consistent with a false discovery rate (FDR) cutoff of 5%. GSEA was performed using Gene Ontology (C5.all) containing 16,107 gene sets.

### Bioinformatics and statistical analyses

Dot plots shown in Fig. 2 A and Fig. 3 B were generated by ProHits-viz. GraphPad Prism software was used to perform two-way ANOVA and an unpaired two-tailed *t* test and to determine the P value and look for significant changes in the data generated from the fusion-expressing cells and TAK-981 treatment *in vivo* or *ex vivo*. All data are expressed as the mean ± SD. For all graphs, *P = 0.01–0.05, **P = 0.001–0.01, and ***P < 0.001.

### Online supplemental material

Table S1 shows a list of 379 significant PML::RARA-associated proteins identified in HPC7 cell lines. Table S2 shows a list of plasmid constructs used in the study. Fig. S1 shows data on PML::RARA-interacting proteins and a detail comparison of PML::RARA interactants identified in HPC7 BioID and Lin^−^ progenitor TurboID. Fig. S2 shows data on exploration of UBC9/RARA and its I396E mutant and impact of TAK-981 in cellular localization of RARA fusion proteins. Fig. S3 shows data on TAK-981 response in primary progenitors.

## Supplementary Material

Table S1 shows a list of 379 significant PML/RARA-associated proteins identified in HPC7 cell lines (FDR < 0.05).

Table S2 lists plasmid constructs used in the study.

SourceData F1 is the source file for Fig. 1.

SourceData F2 is the source file for Fig. 2.

SourceData F3 is the source file for Fig. 3.

SourceData F4 is the source file for Fig. 4.

SourceData FS1 is the source file for Fig. S1.

SourceData FS2 is the source file for Fig. S2.

## Data Availability

The mass spectrometry proteomics data (Fig 2 and Fig 3) have been deposited to the ProteomeXchange consortium via the PRIDE partner repository (Perez-Riverol et al., 2025) with the dataset identifier PXD078941. All data are available upon reasonable request from the corresponding authors.
