# Correction: PML-driven sumoylation of PML::RARA-bound repressors drives hematopoietic progenitor immortalization

**DOI:** 10.1084/jem.2025233309092026c

**Published:** 2026-09-22

**Authors:** Hsin Chieh Wu, Emmanuel Laplantine, Cécile Esnault, Michiko Niwa-Kawakita, Yi Zhang, Viviane De Almeida Bastos, Marie-Claude Geoffroy, Hugues de Thé

Vol. 223, No. 9 | https://doi.org/10.1084/jem.20252333 | August 28, 2026

*JEM* regrets that the bottom six blots in Fig. 3 E were mistakenly deleted during the production process. In addition, the “Input” and “Ni-NTA purification” label bars were misaligned. The original and corrected figures are shown here. This correction does not change the original conclusions of the article, and the figure legend remains unchanged. The HTML and PDF versions of this article have been corrected. The errors remain only in print and in PDFs downloaded before September 10, 2026.

**Figure:**
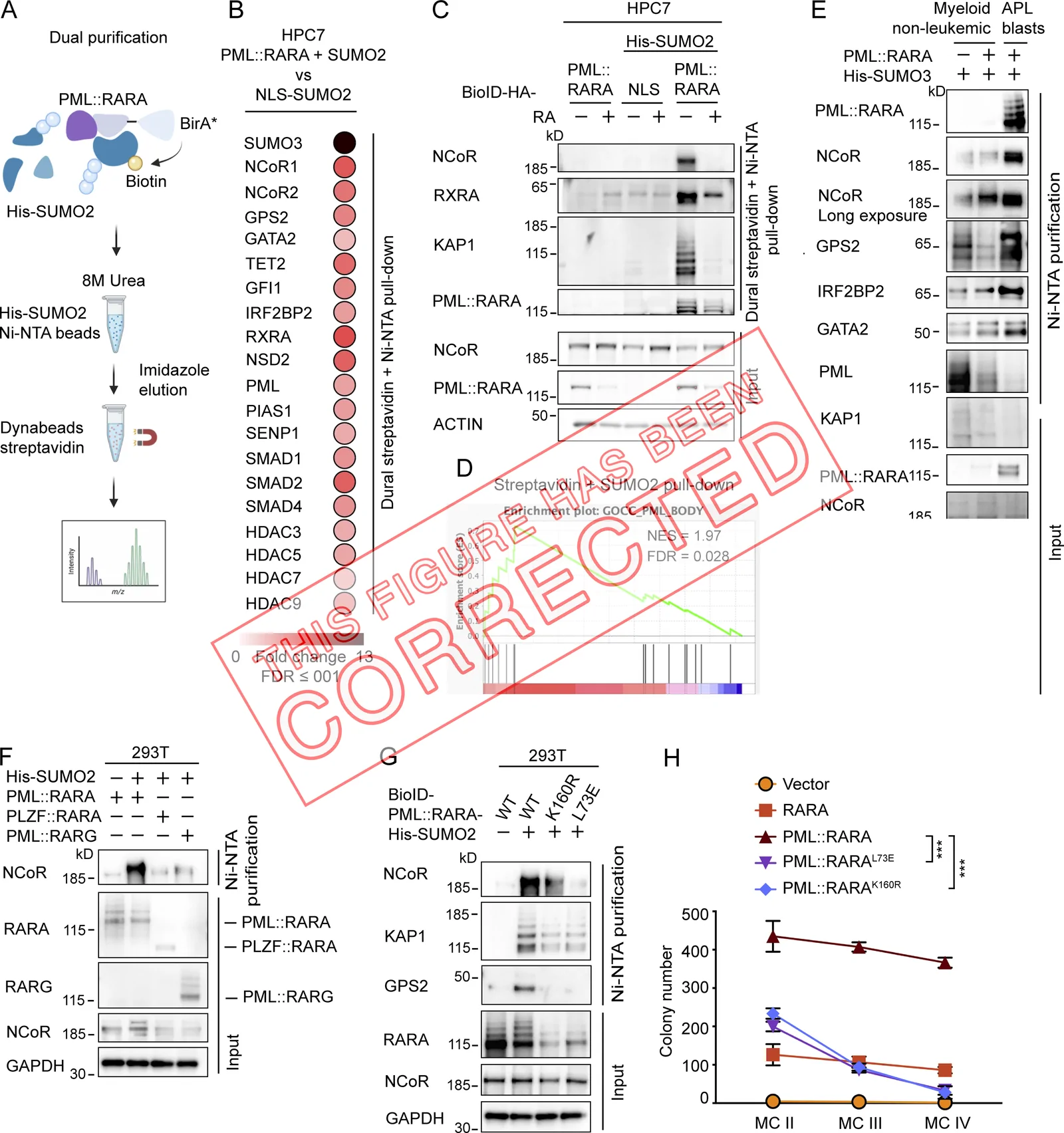


**Figure 3.**
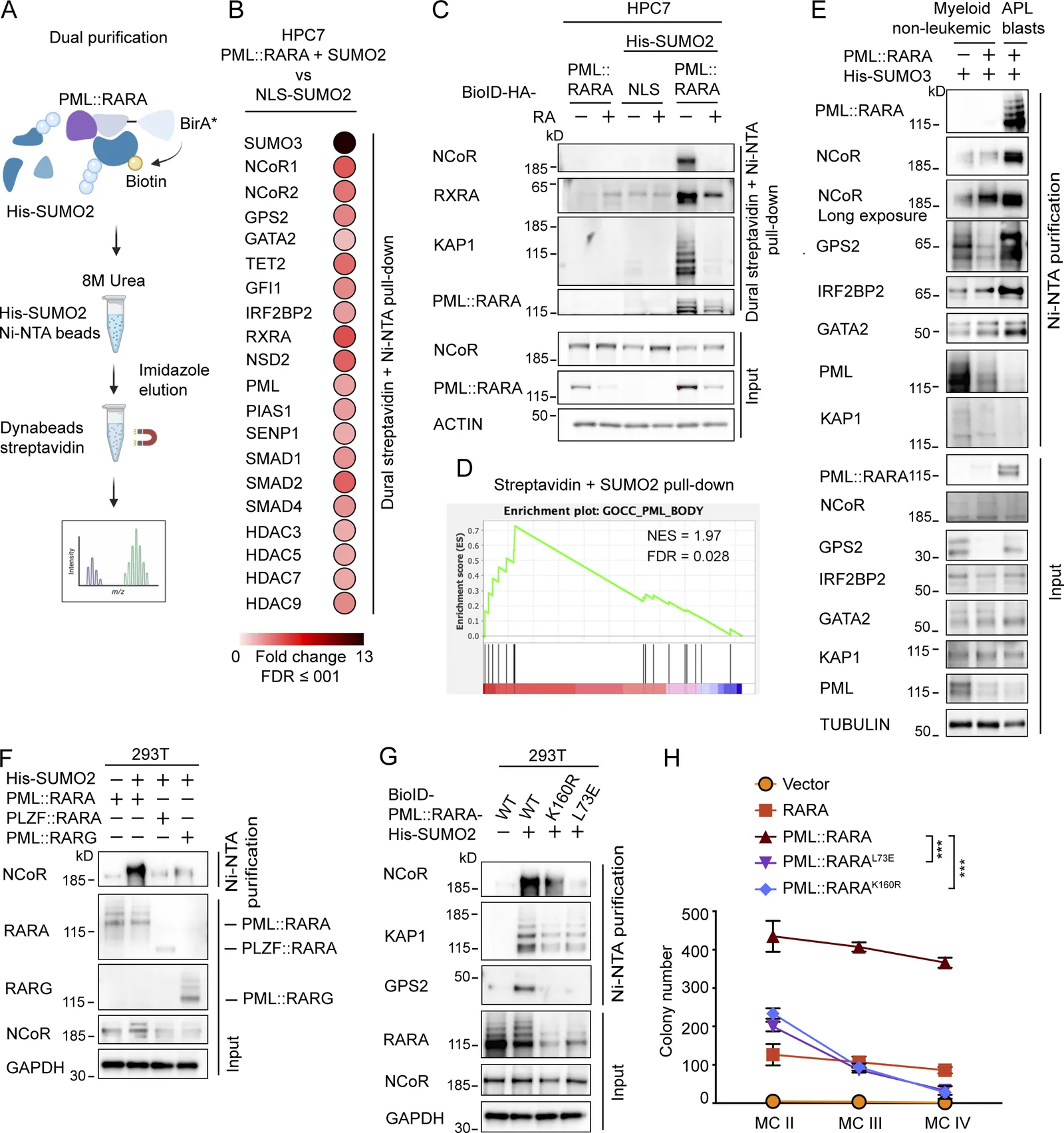
**Fusion of PML to RARA promotes SUMO2 conjugation of RARA-associated partners. (A)** Experimental scheme of Ni-NTA plus streptavidin double-purification strategy used for mass spectrometry analysis of HPC7 cells expressing His_10_-SUMO2 together with NLS-BioID or PML::RARA-BioID. **(B)** Dot plot of the top 20 PML::RARA-BioID (*N* = 4) interactors compared to NLS-BioID (*N* = 4), with significant SUMO2 conjugation in His_10_-SUMO_2_–expressing HPC7 cells. **(C)** Western blot validation of lysates and Ni-NTA– plus streptavidin-purified fractions from HPC7-expressing PML::RARA-BioID alone or co-expressing His_10_-SUMO2 with NLS-BioID or PML::RARA-BioID. Cells were pretreated with or without RA (1 μM) for 2 h, followed by biotin (50 μM) addition, with RA treatment continued for a total of 18 h. *N* = 3. **(D)** GSEA of proteomics data from His_10_-SUMO2 HPC7 cells expressing PML::RARA-BioID versus NLS-BioID reveals PML_BODY as the sole significantly enriched pathway. **(E)** Western blot analysis of His_10_-SUMO_3_ knock-in mice expressing PML::RARA at nonleukemic stage (*N* = 4) or full-blown APL blasts (*N* = 6) following Ni-NTA purification. **(F)** Western blot analysis (NCoR antibody) of lysates and Ni-NTA–purified fractions from 293T cells transfected with His_10_-SUMO2 and indicated RAR fusion constructs. *N* = 2. **(G)** Western blot analysis of lysates and Ni-NTA– plus streptavidin double-purified fractions from 293T cells transfected with His_10_-SUMO2 and the indicated PML::RARA mutants. *N* = 2. **(H)** Colony counts of Lin^−^ progenitors transformed with RARA and PML::RARA mutants at the indicated replating. Cells were replated every 5–7 days; MC II, III, and IV denote the second, third, and fourth methylcellulose replating. *N* = 2. Data are presented as the mean ± SD of three technical triplicates from one representative experiment out of at least two biologically independent experiments. Statistical significance was assessed by two-way ANOVA, ***P < 0.001. Source data are available for this figure: SourceData F3.

