# Supplementary material for: PML-driven sumoylation of PML::RARA-bound repressors drives hematopoietic progenitor immortalization
Source: J Exp Med. 2026 Aug 28;223(9):e20252333. doi: 10.1084/jem.20252333 (PMC13523716; doi:10.1084/jem.20252333)
Supplement: Supplementary file 1 [file jem_20252333_sourcedataf1.pdf]

Fig. 1B (left)

Lin- progenitors (MC II)

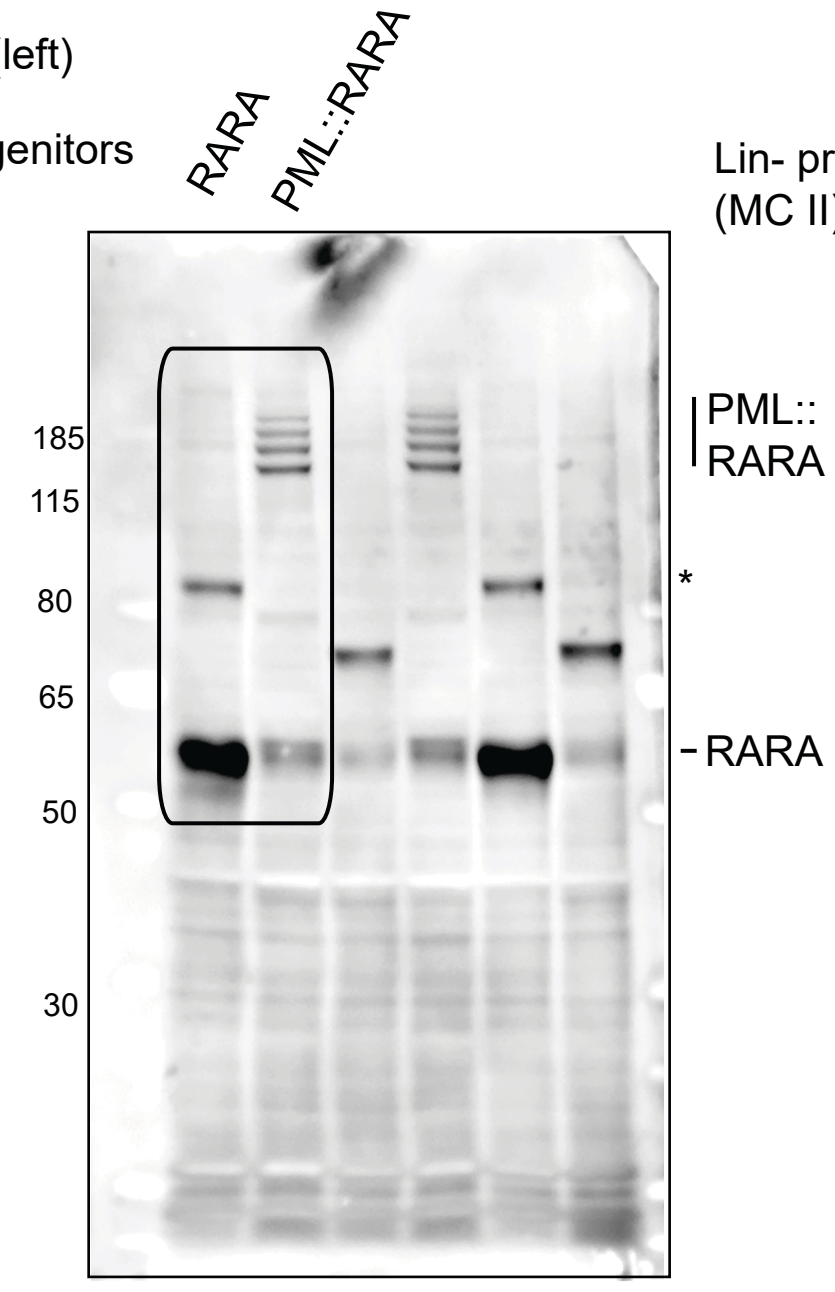

WB: RARA

Lin- progenitors (MC II)

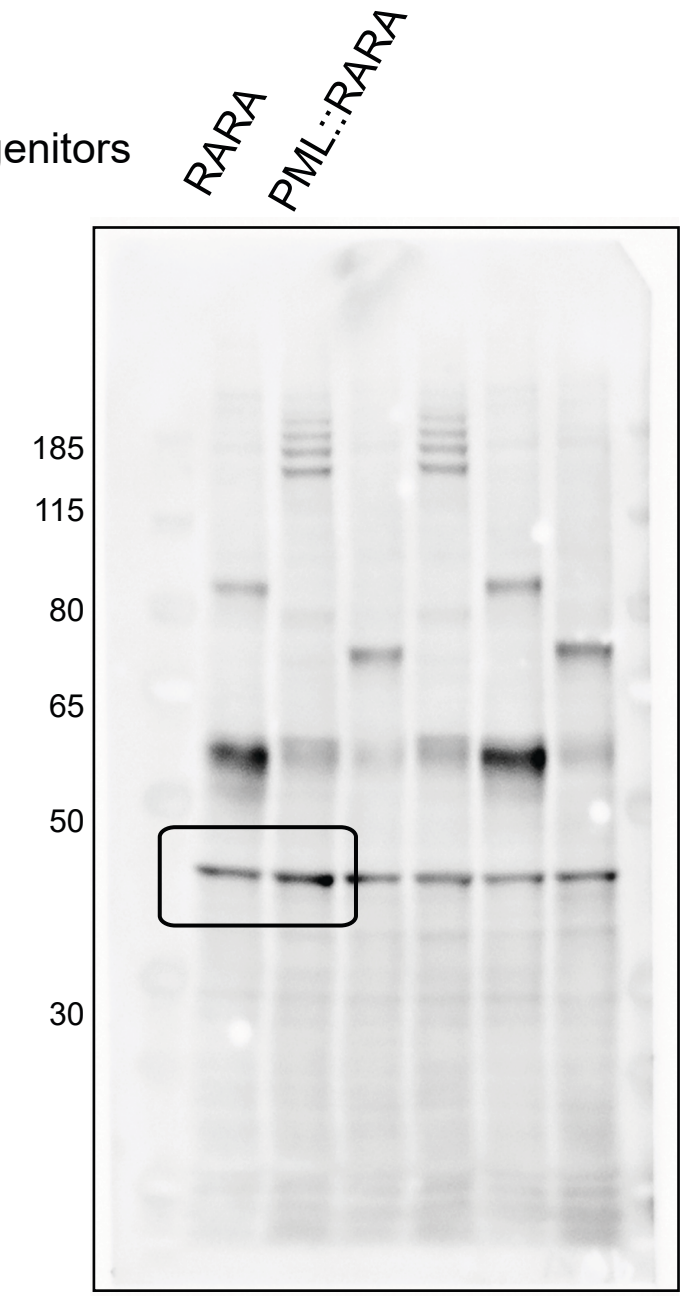

WB: ACTIN

Fig. 1B (left)

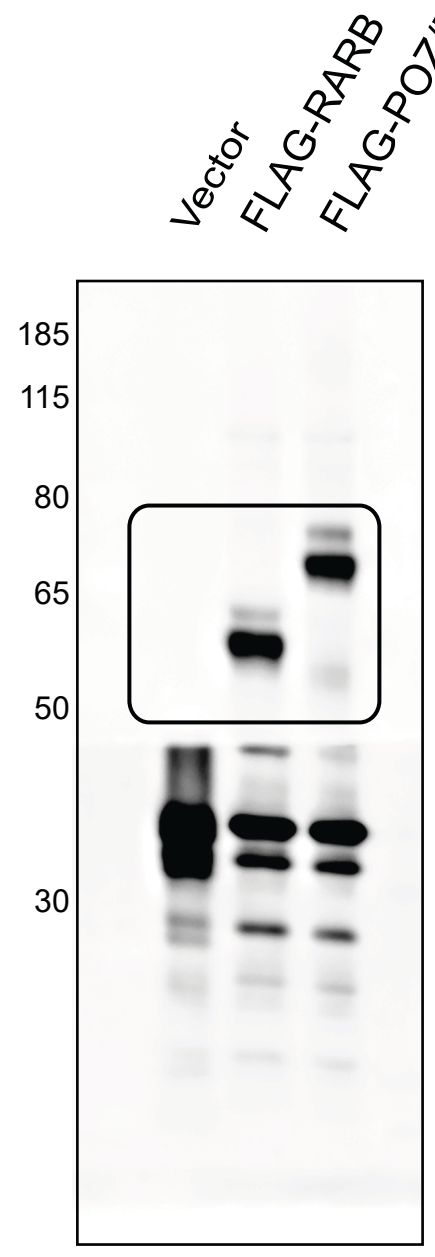

WB: FLAG

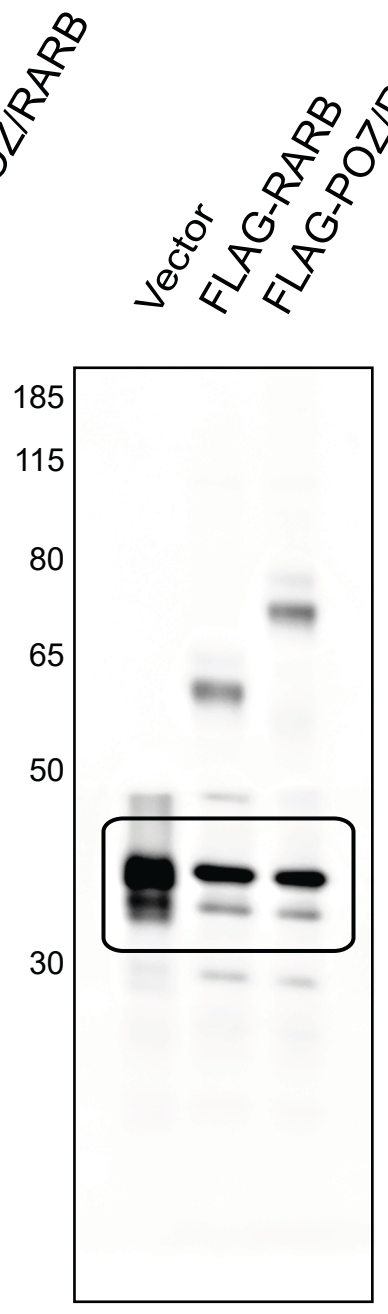

WB: GAPDH

Fig. 1E (right)

Lin⁻ progenitors (MC II)

WB: RARA

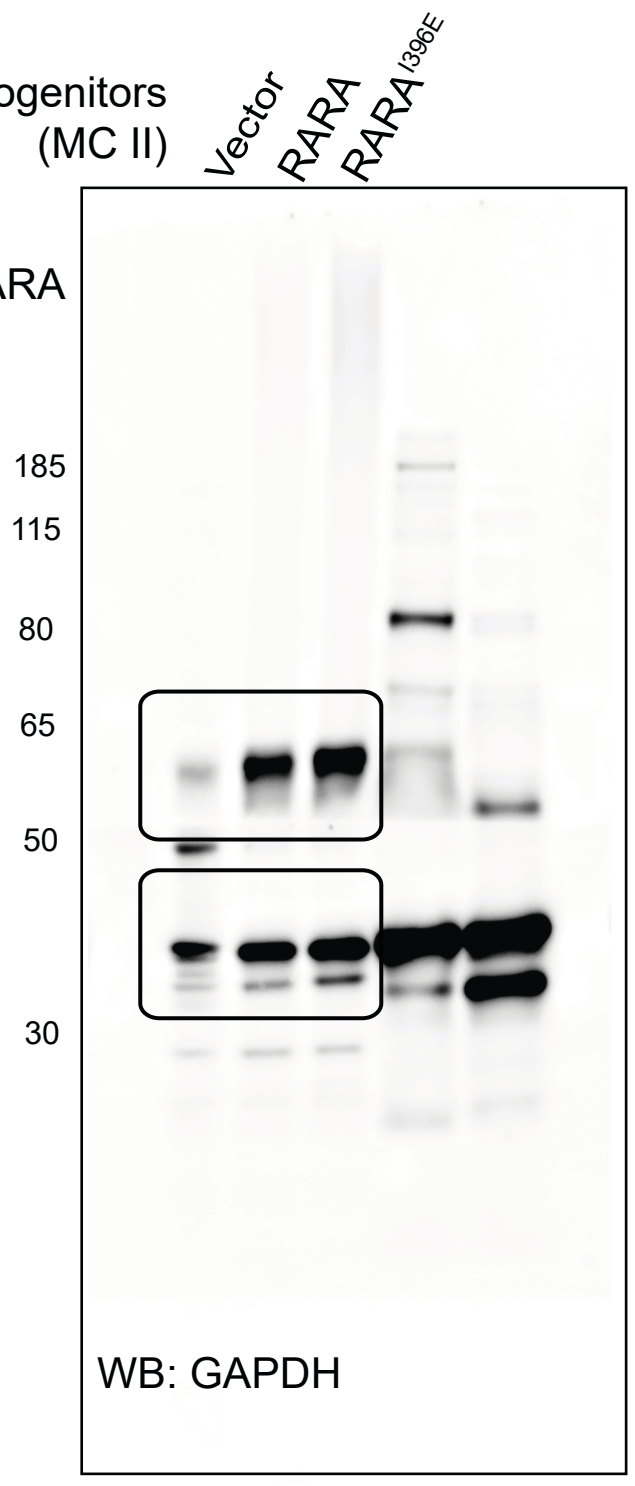

Fig. 1E (left)

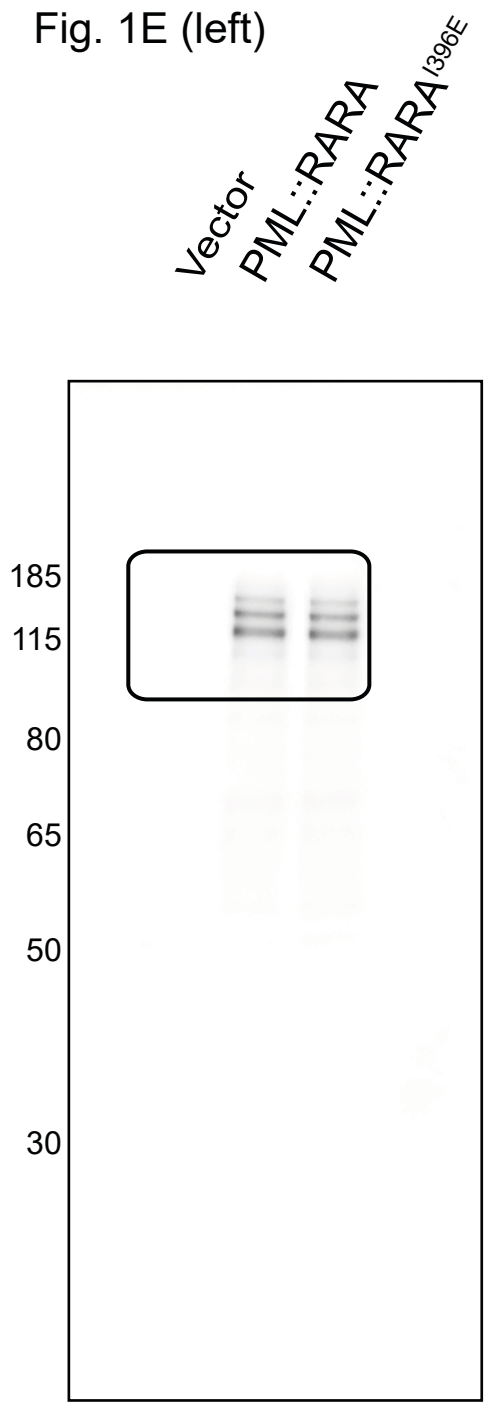

WB: RARA

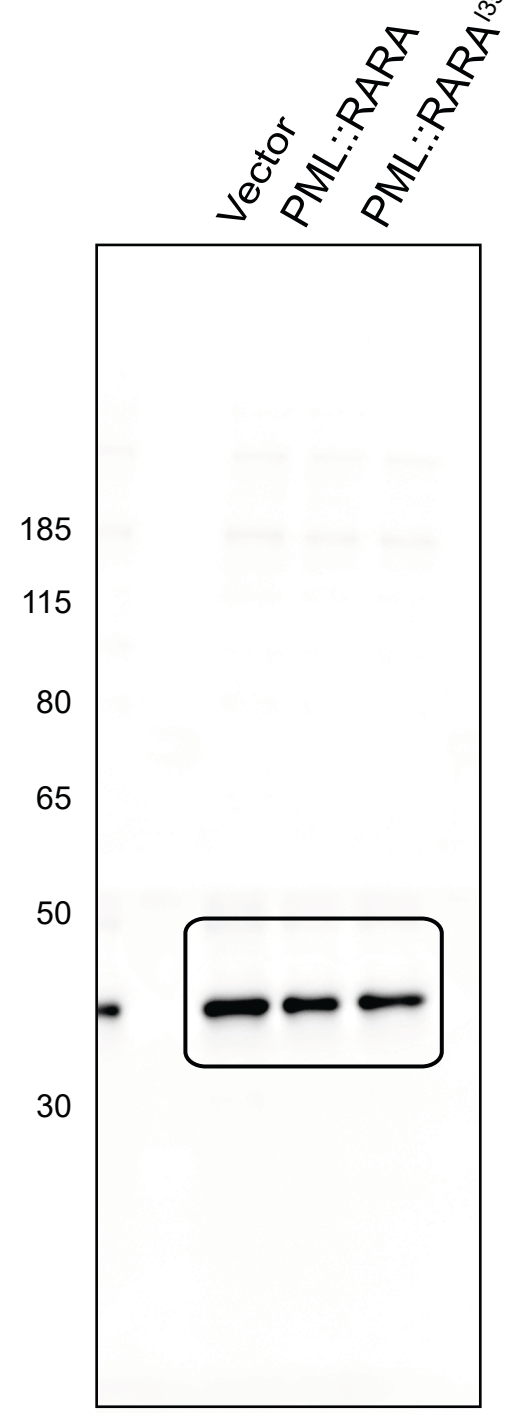

WB: GAPDH

Fig. 1E (left)

Long exposure

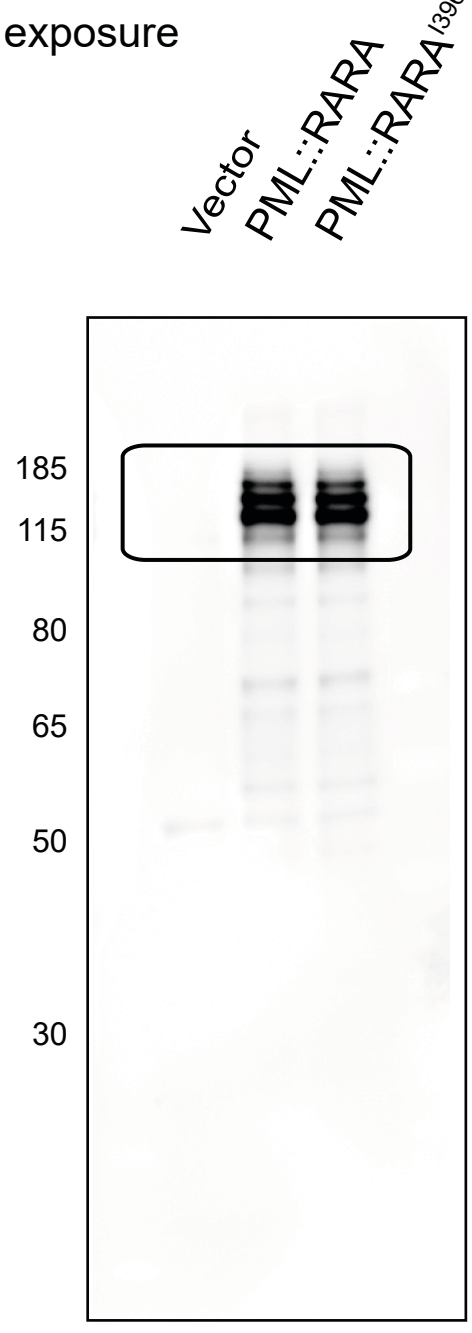

WB: RARA

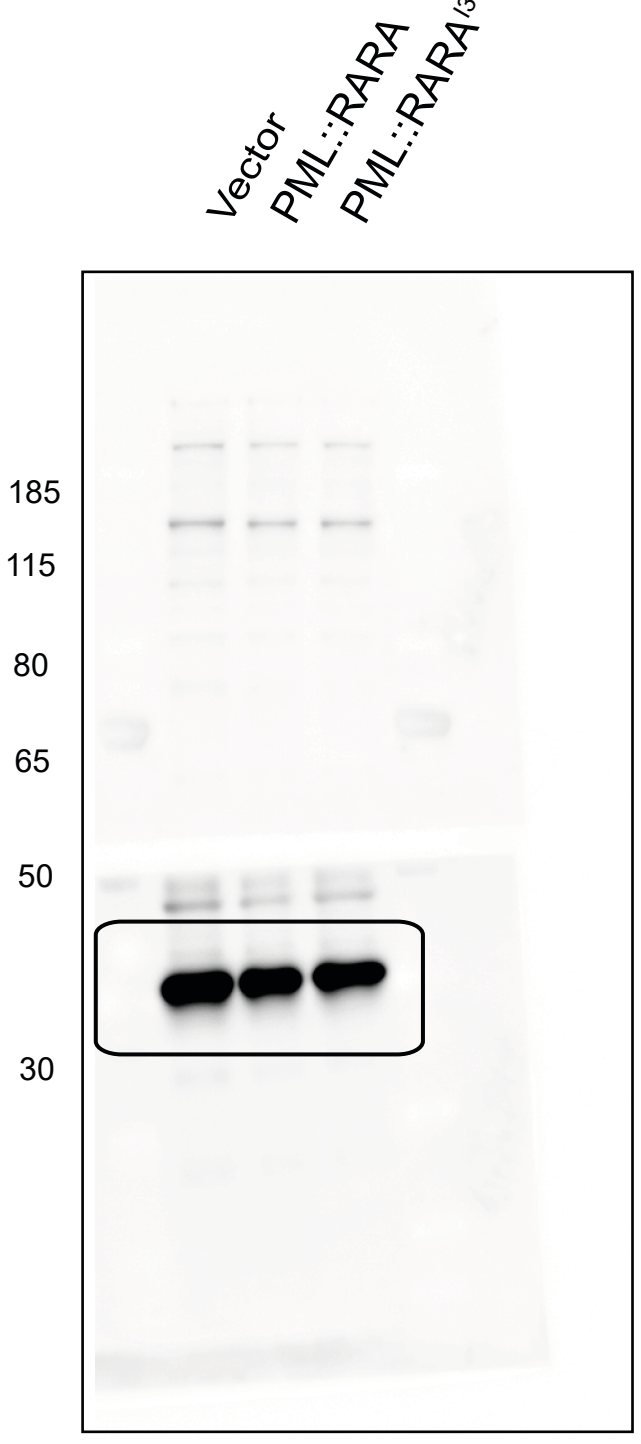

WB: GAPDH
