# Supplementary material for: PML-driven sumoylation of PML::RARA-bound repressors drives hematopoietic progenitor immortalization
Source: J Exp Med. 2026 Aug 28;223(9):e20252333. doi: 10.1084/jem.20252333 (PMC13523716; doi:10.1084/jem.20252333)

Fig. 2B

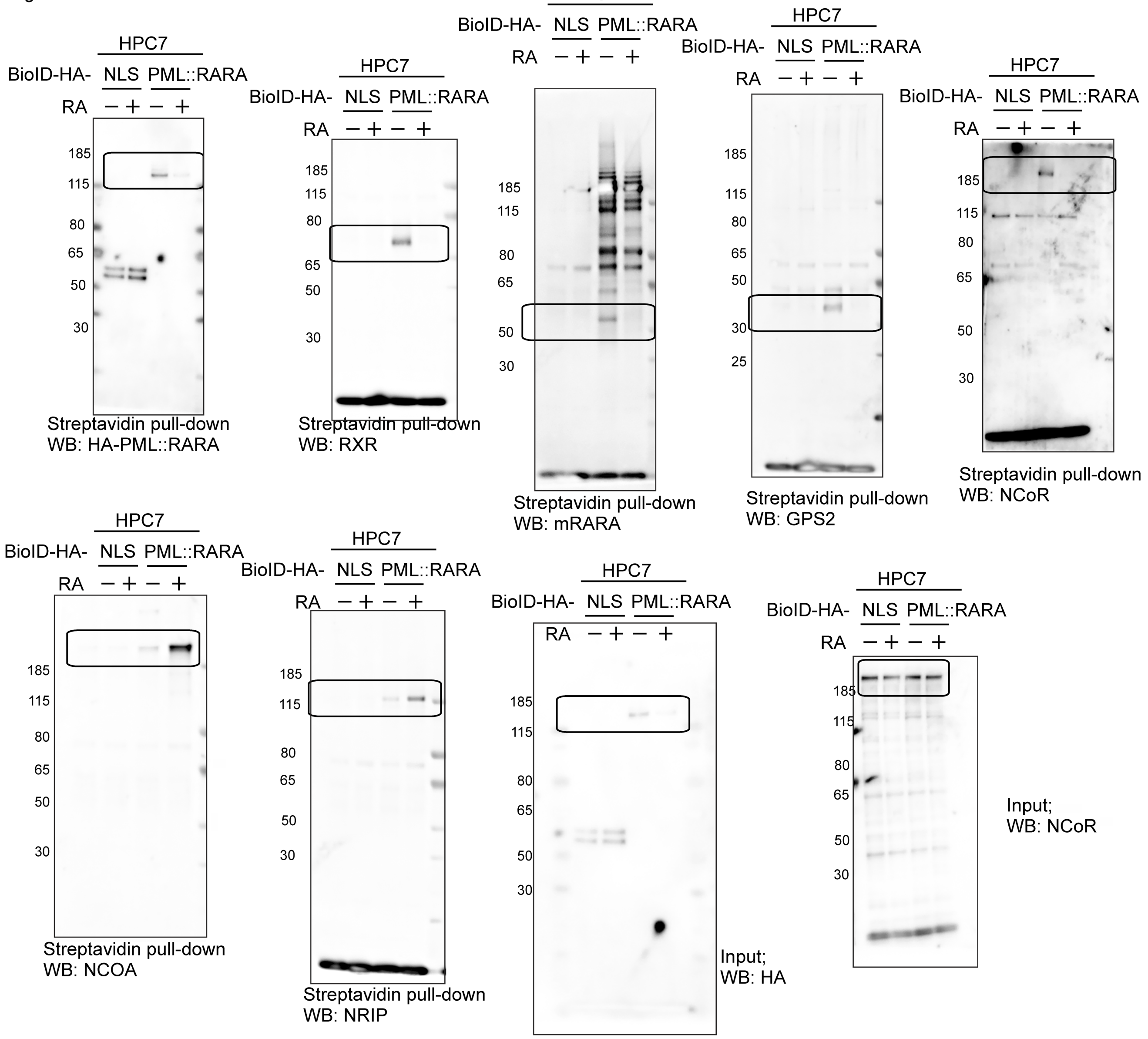
HPC7
BioID-HA- NLS PML::RARA
RA − + − +
Streptavidin pull-down
WB: HA-PML::RARA
Streptavidin pull-down
WB: RXR
Streptavidin pull-down
WB: mRARA
Streptavidin pull-down
WB: GPS2
Streptavidin pull-down
WB: NCoR
Streptavidin pull-down
WB: NCOA
Streptavidin pull-down
WB: NRIP
Input;
WB: HA
Input;
WB: NCoR

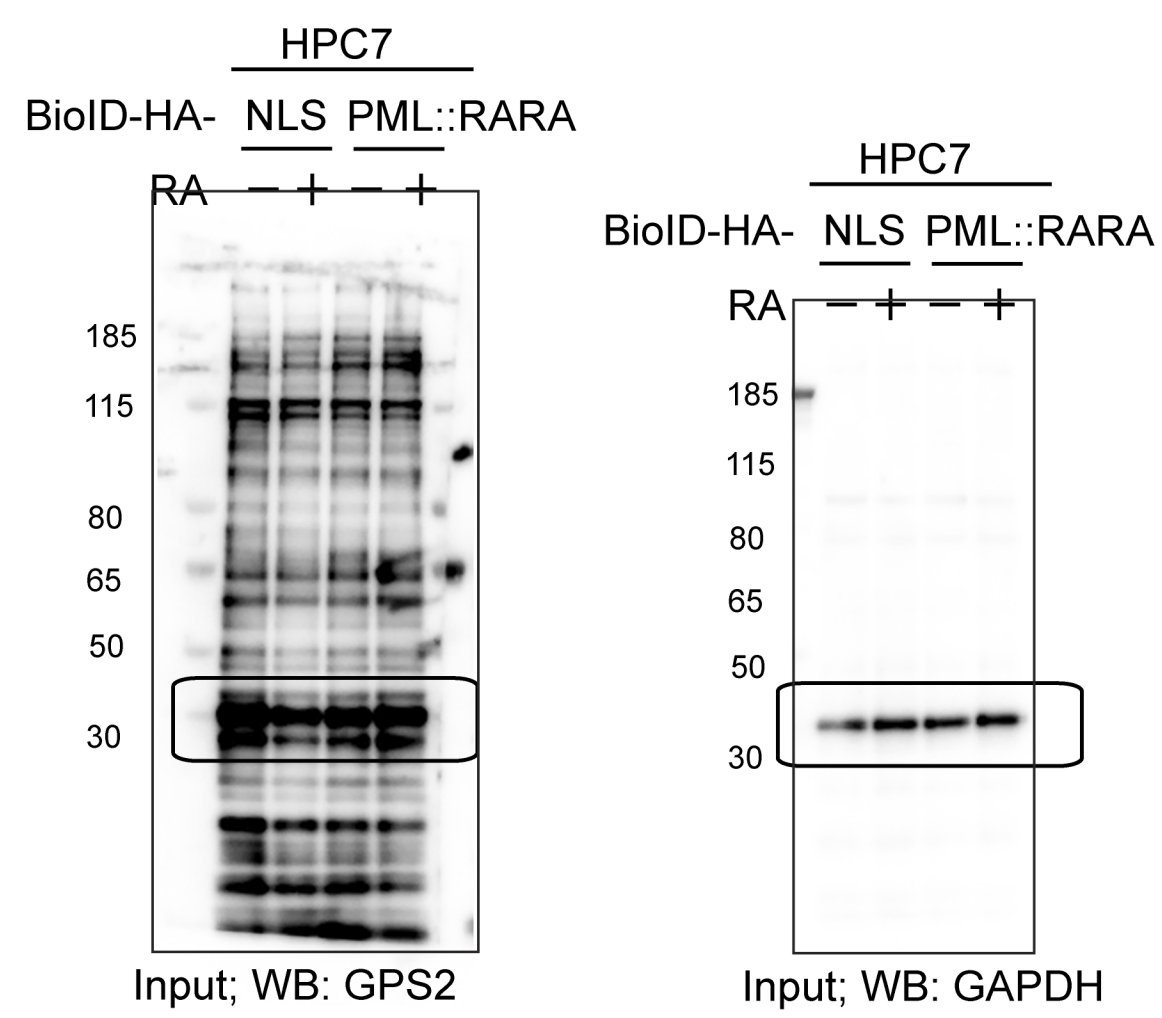
HPC7
BioID-HA- NLS PML::RARA
RA − + − +
Input; WB: GPS2
Input; WB: GAPDH

Fig. 2D

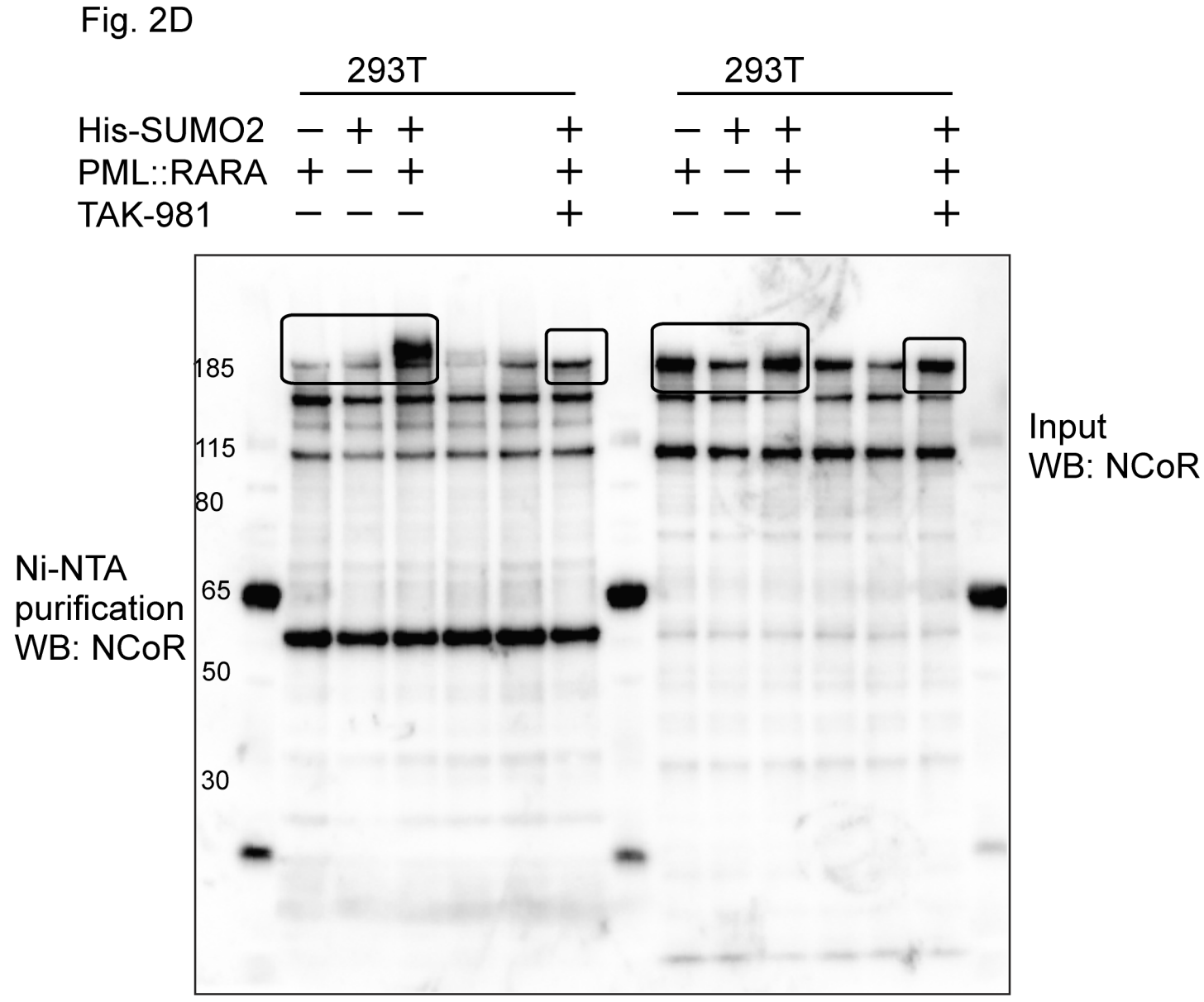
293T
293T
His-SUMO2 − + + + − + + +
PML::RARA + − + + + − + +
TAK-981 − − − + − − − +
Ni-NTA
purification
WB: NCoR
Input
WB: NCoR
185
115
80
65
50
30

Fig. 2E

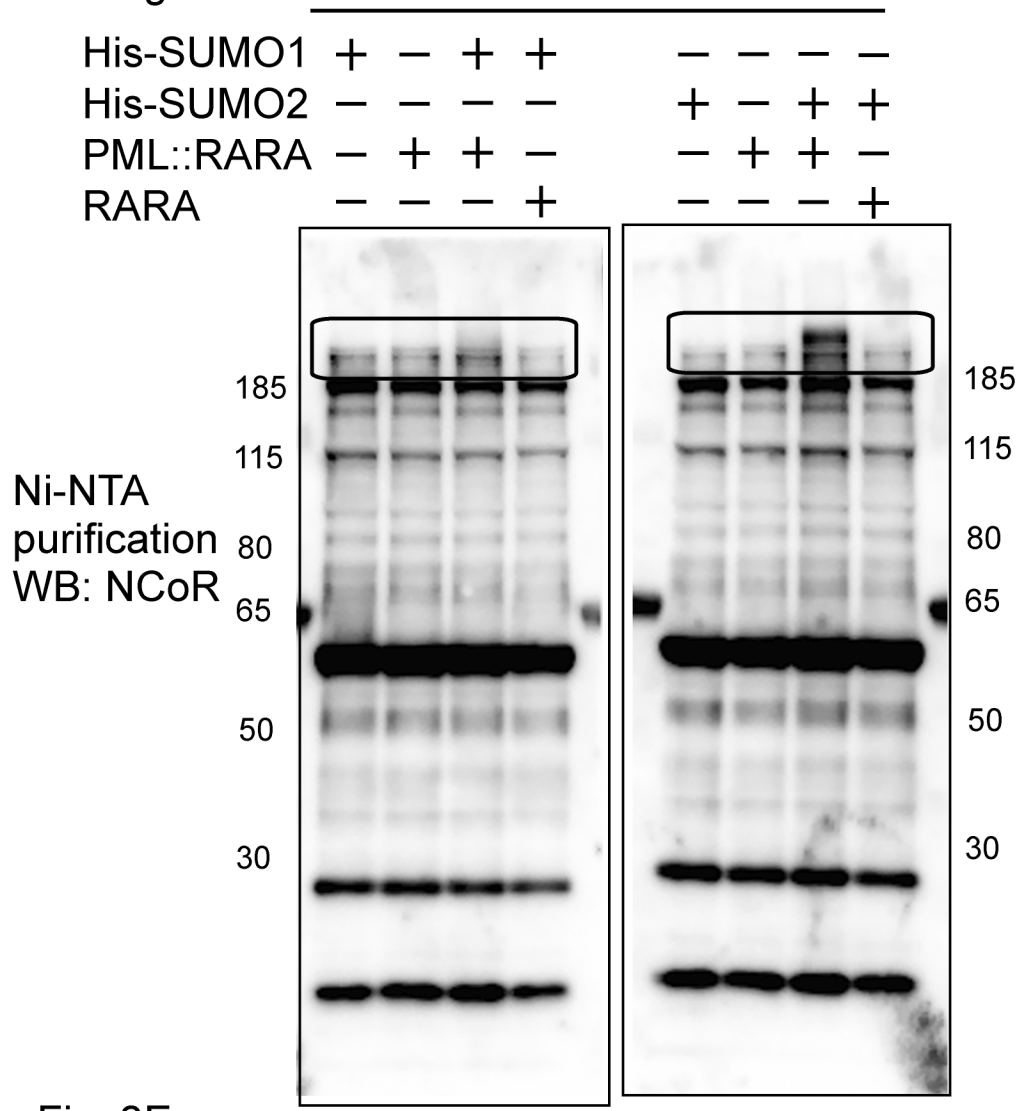

Fig. 2D

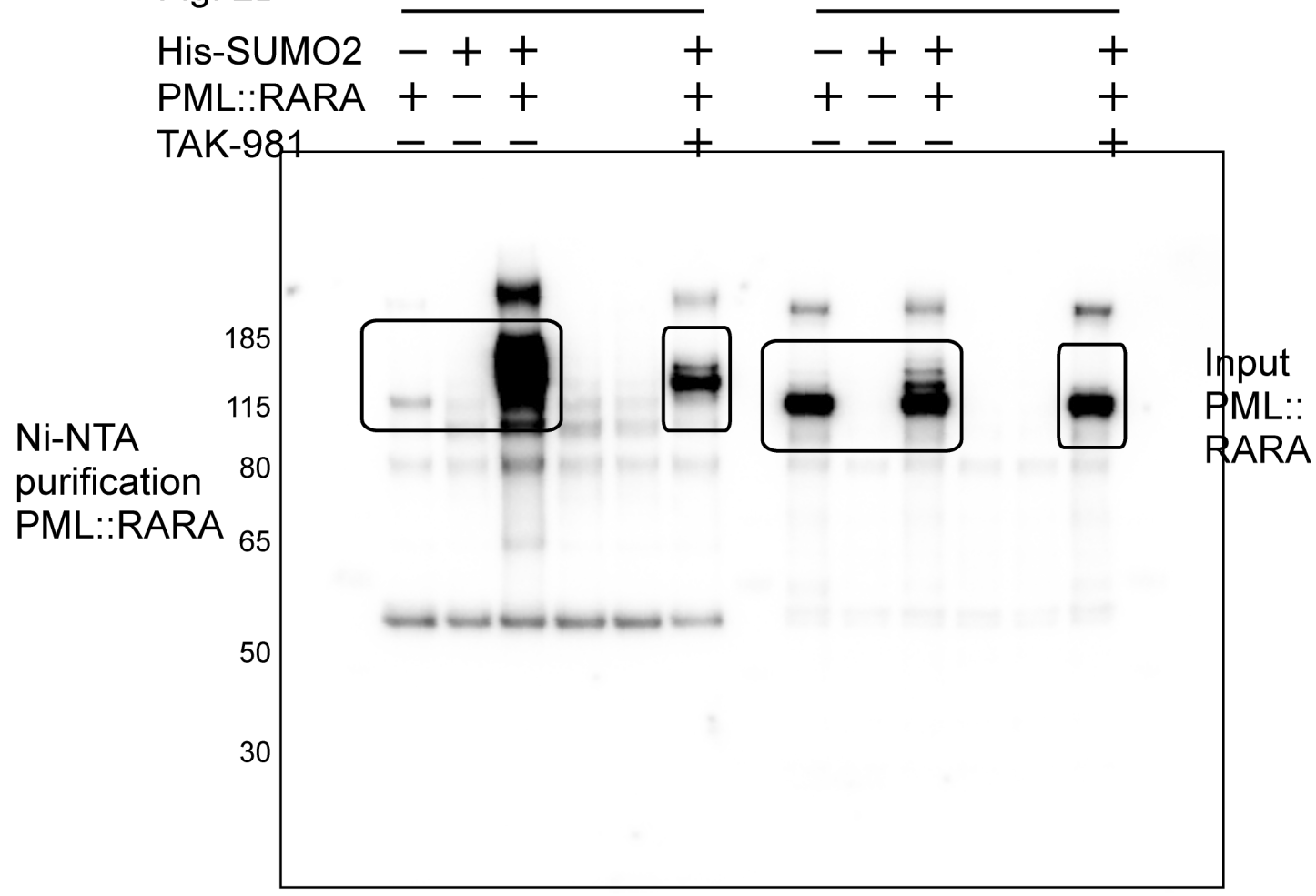

Fig. 2E

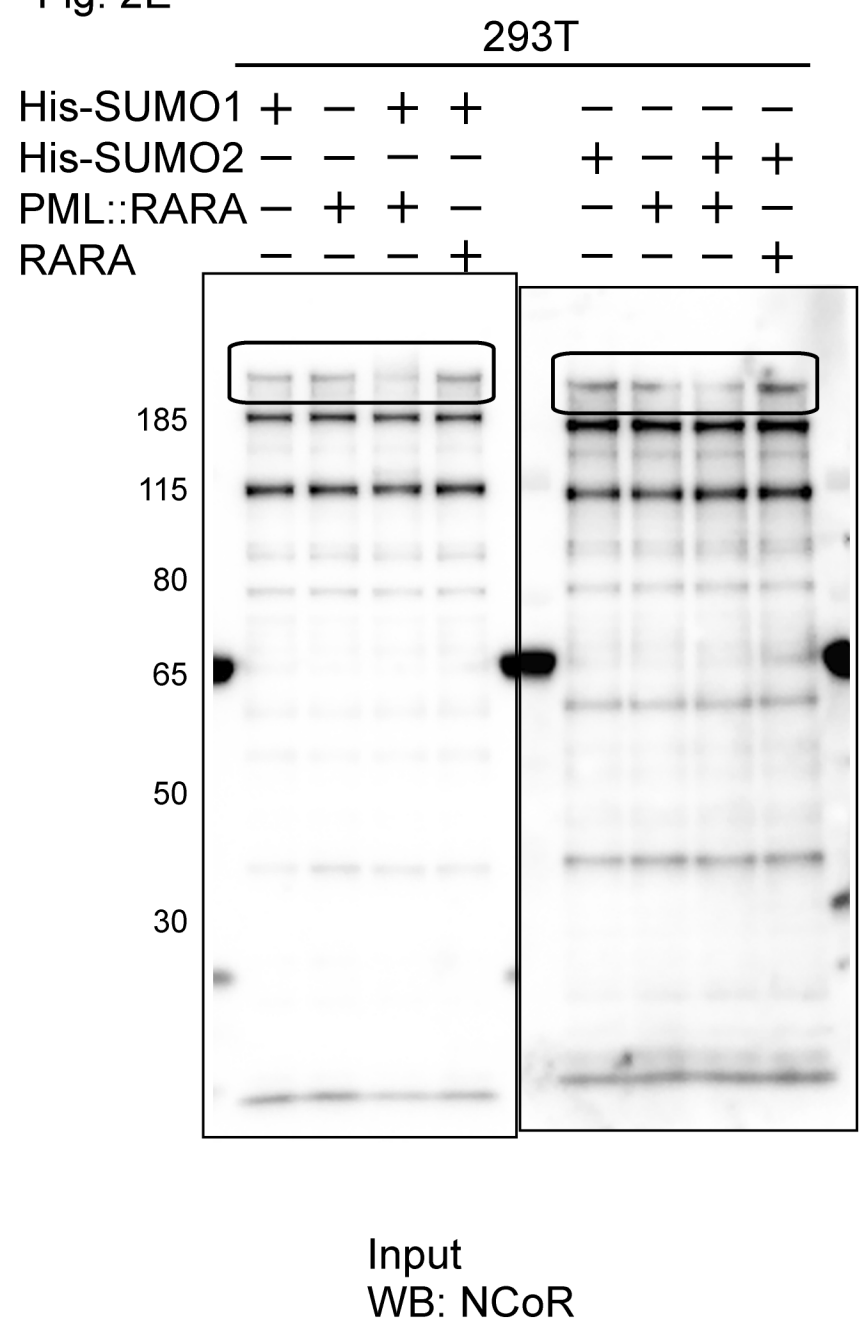

Fig. 2E

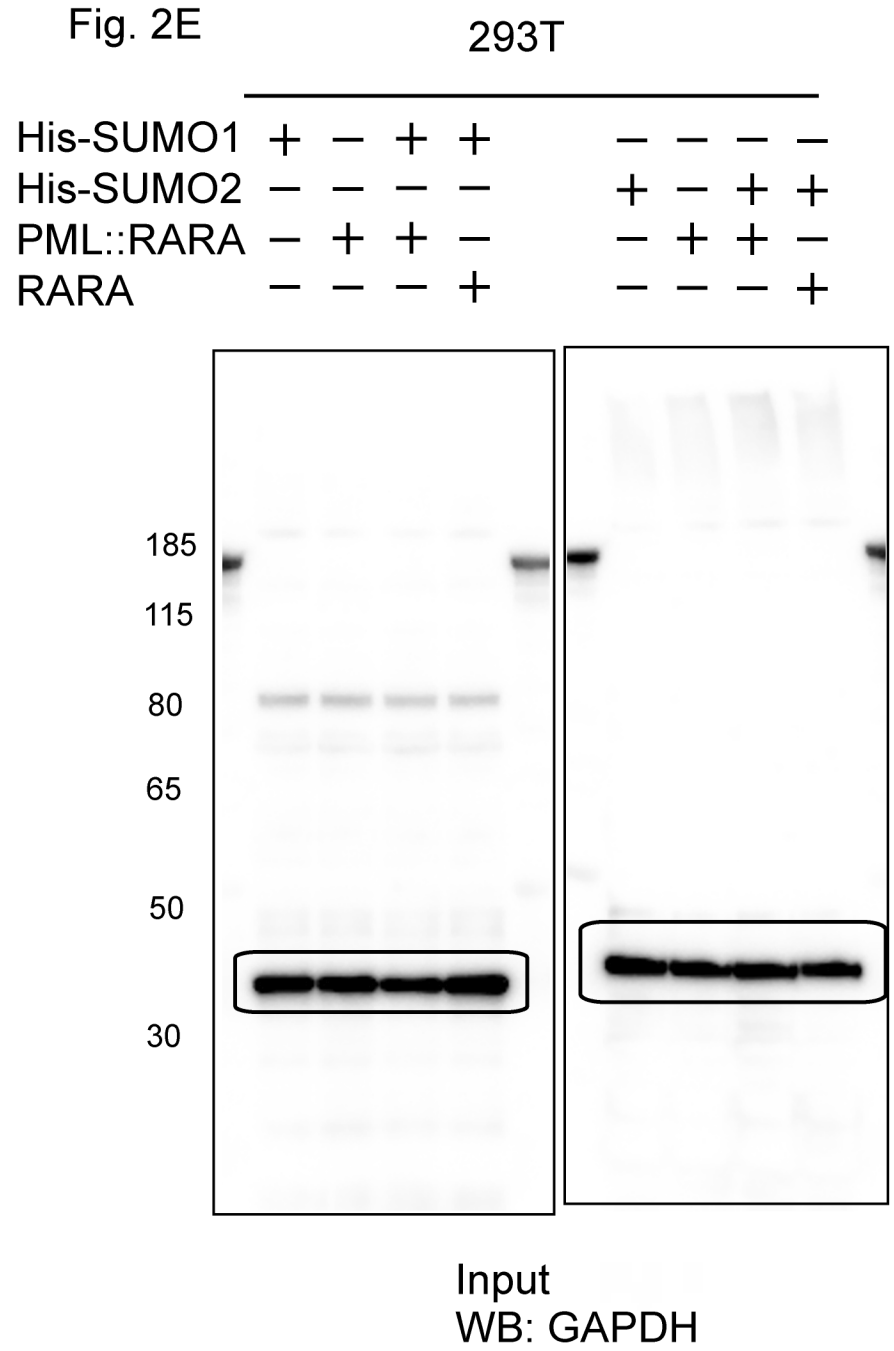

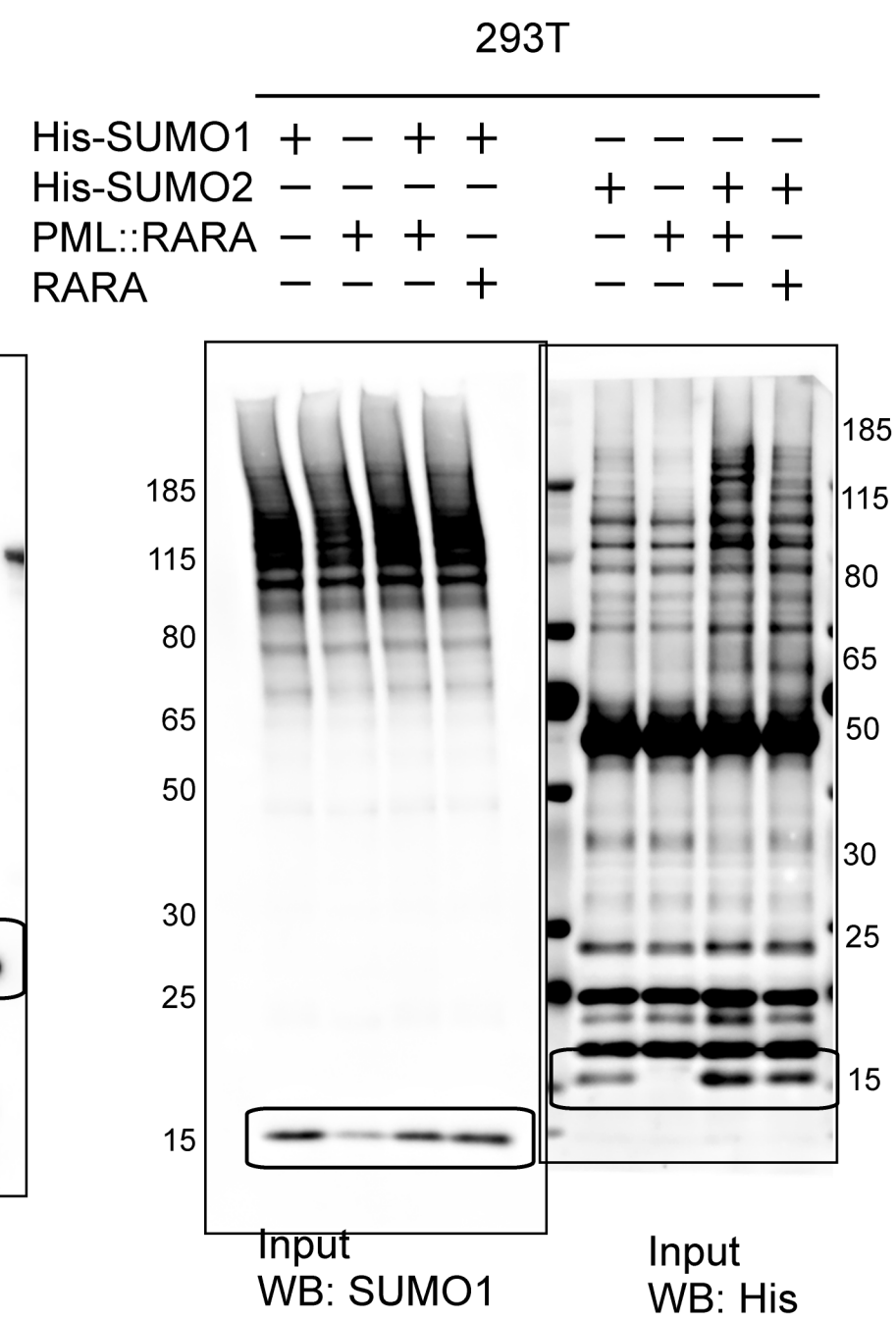

Fig. 2E

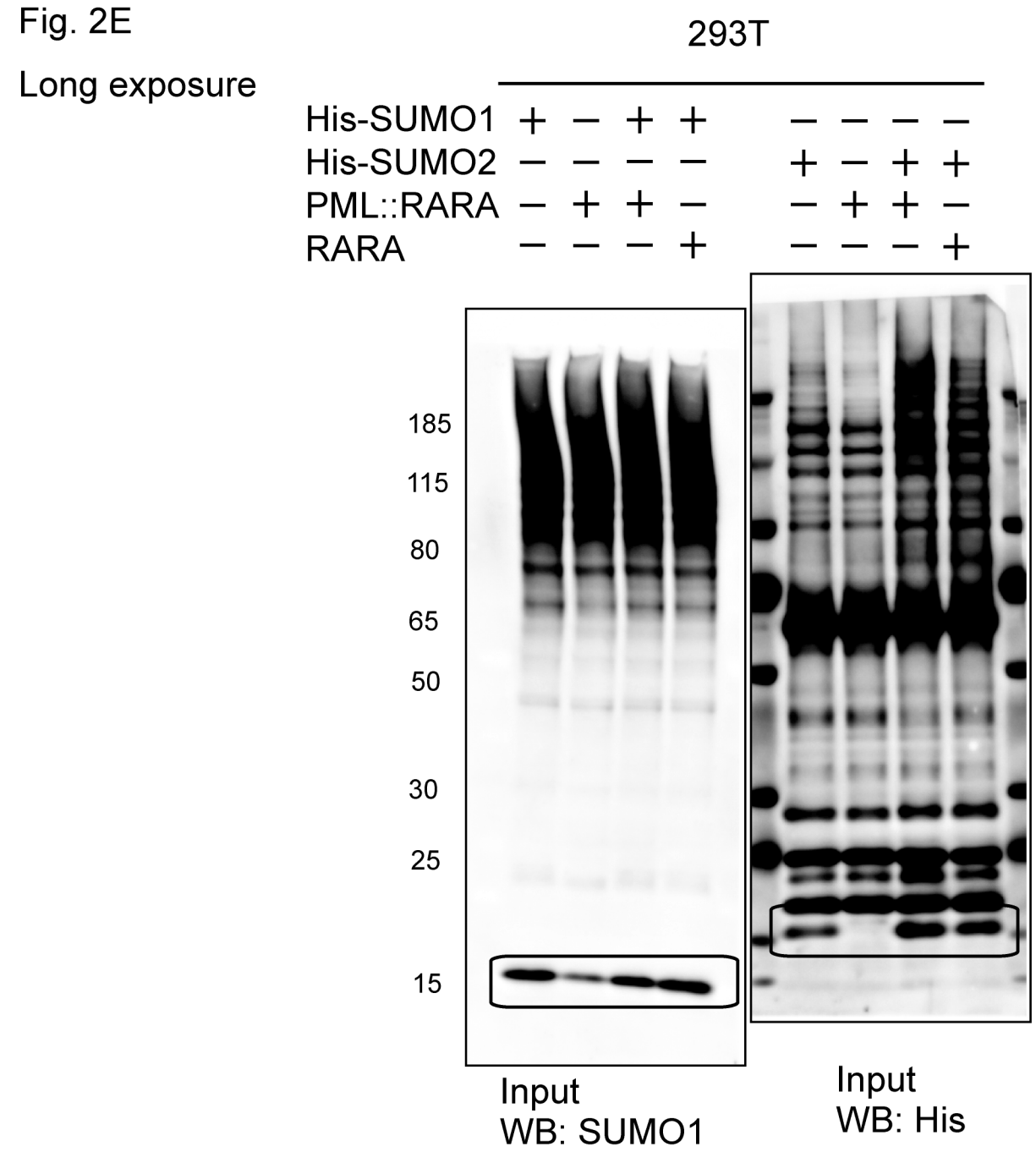

Supplement: Supplementary file 2 [file jem_20252333_sourcedataf2.pdf]
