# Supplementary material for: PML-driven sumoylation of PML::RARA-bound repressors drives hematopoietic progenitor immortalization
Source: J Exp Med. 2026 Aug 28;223(9):e20252333. doi: 10.1084/jem.20252333 (PMC13523716; doi:10.1084/jem.20252333)
Supplement: Supplementary file 3 [file jem_20252333_sourcedataf3.pdf]

Fig. 3C

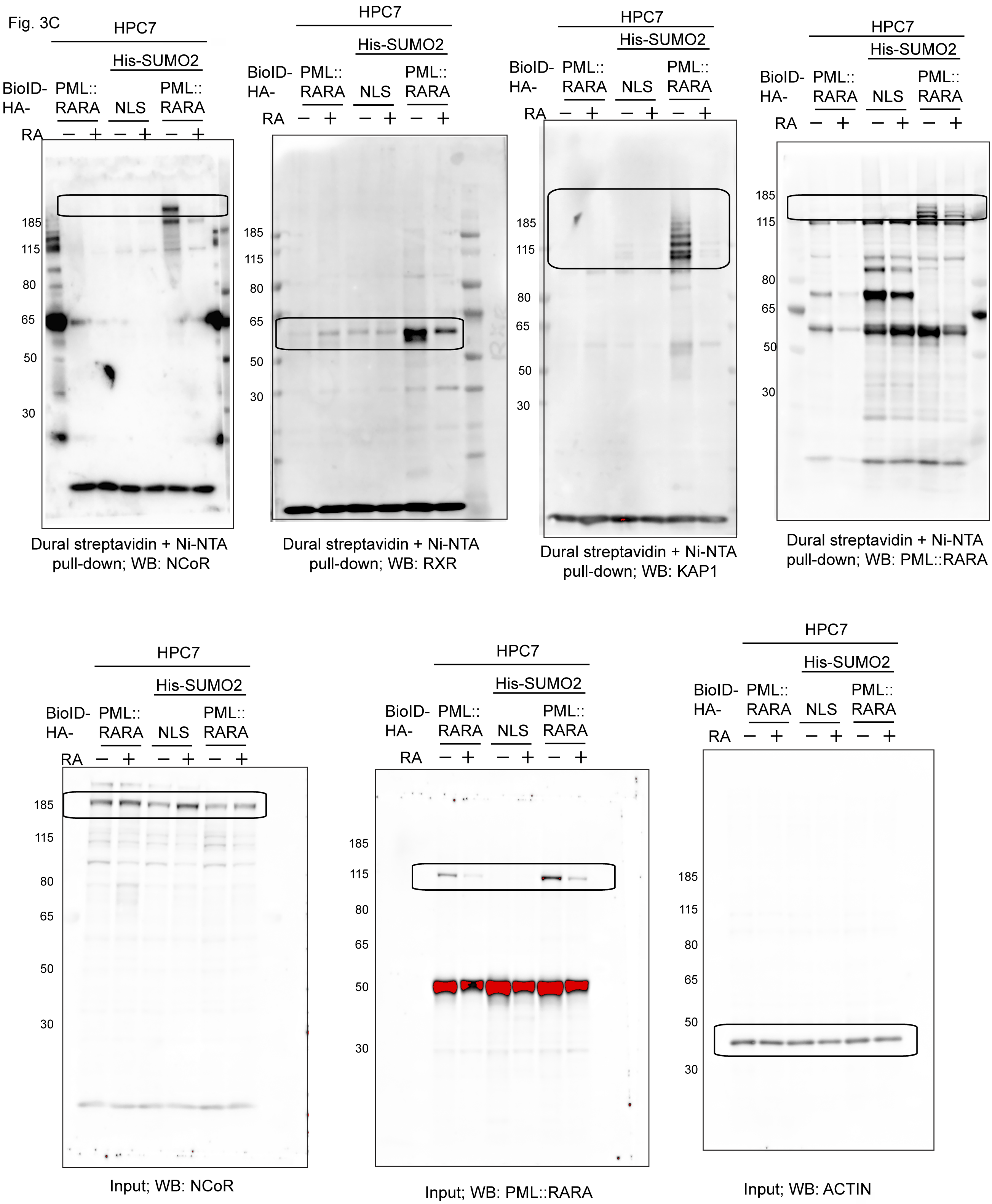

Dural streptavidin + Ni-NTA pull-down; WB: NCoR

Dural streptavidin + Ni-NTA pull-down; WB: RXR

Dural streptavidin + Ni-NTA pull-down; WB: KAP1

Dural streptavidin + Ni-NTA pull-down; WB: PML::RARA

Input; WB: NCoR

Input; WB: PML::RARA

Input; WB: ACTIN

Fig. 3E

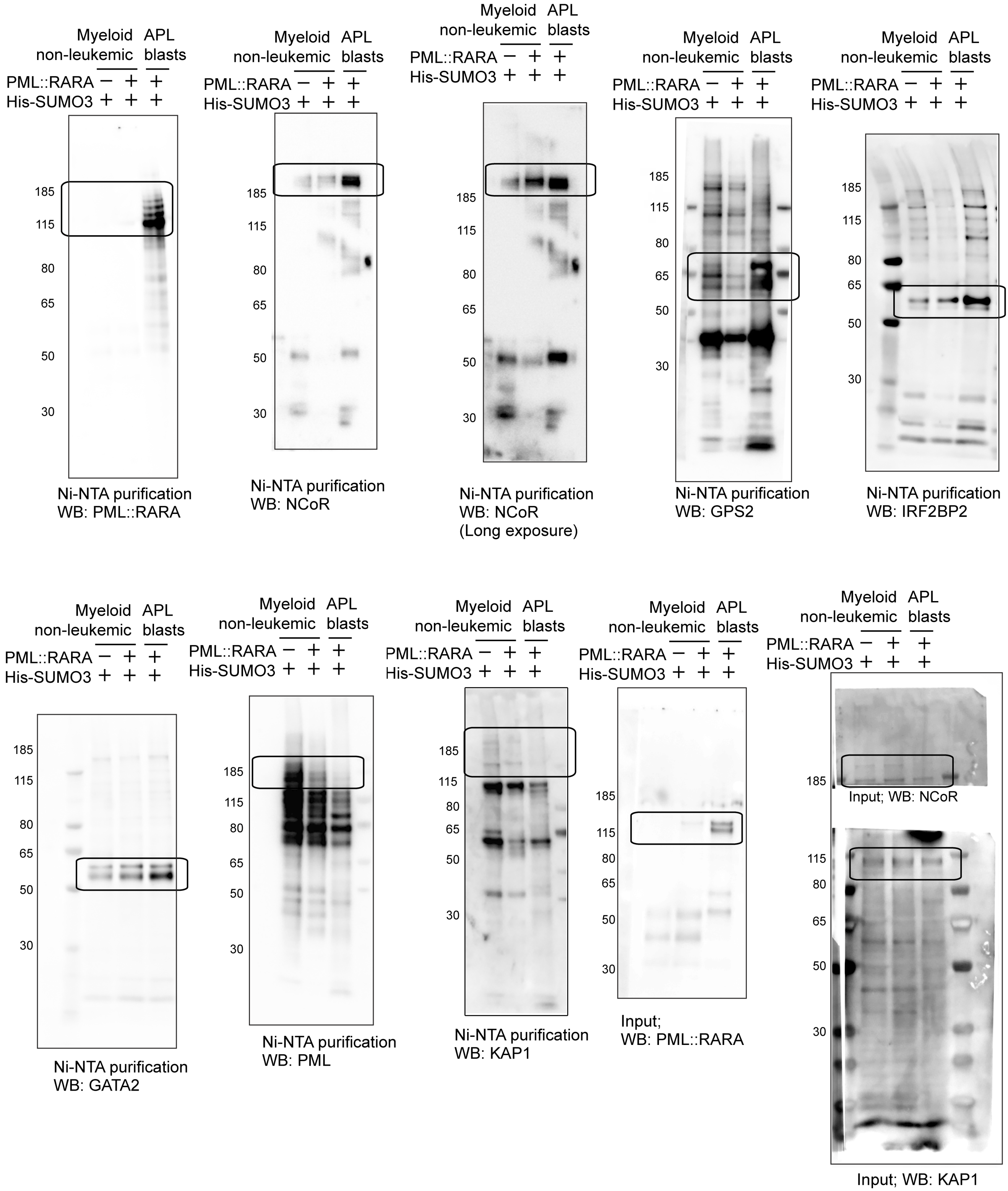
Myeloid non-leukemic
APL blasts
PML::RARA − + +
His-SUMO3 + + +
185
115
80
65
50
30
Ni-NTA purification
WB: PML::RARA
Ni-NTA purification
WB: NCoR
Ni-NTA purification
WB: NCoR
(Long exposure)
Ni-NTA purification
WB: GPS2
Ni-NTA purification
WB: IRF2BP2
Ni-NTA purification
WB: GATA2
Ni-NTA purification
WB: PML
Ni-NTA purification
WB: KAP1
Input;
WB: PML::RARA
Input; WB: NCoR
Input; WB: KAP1

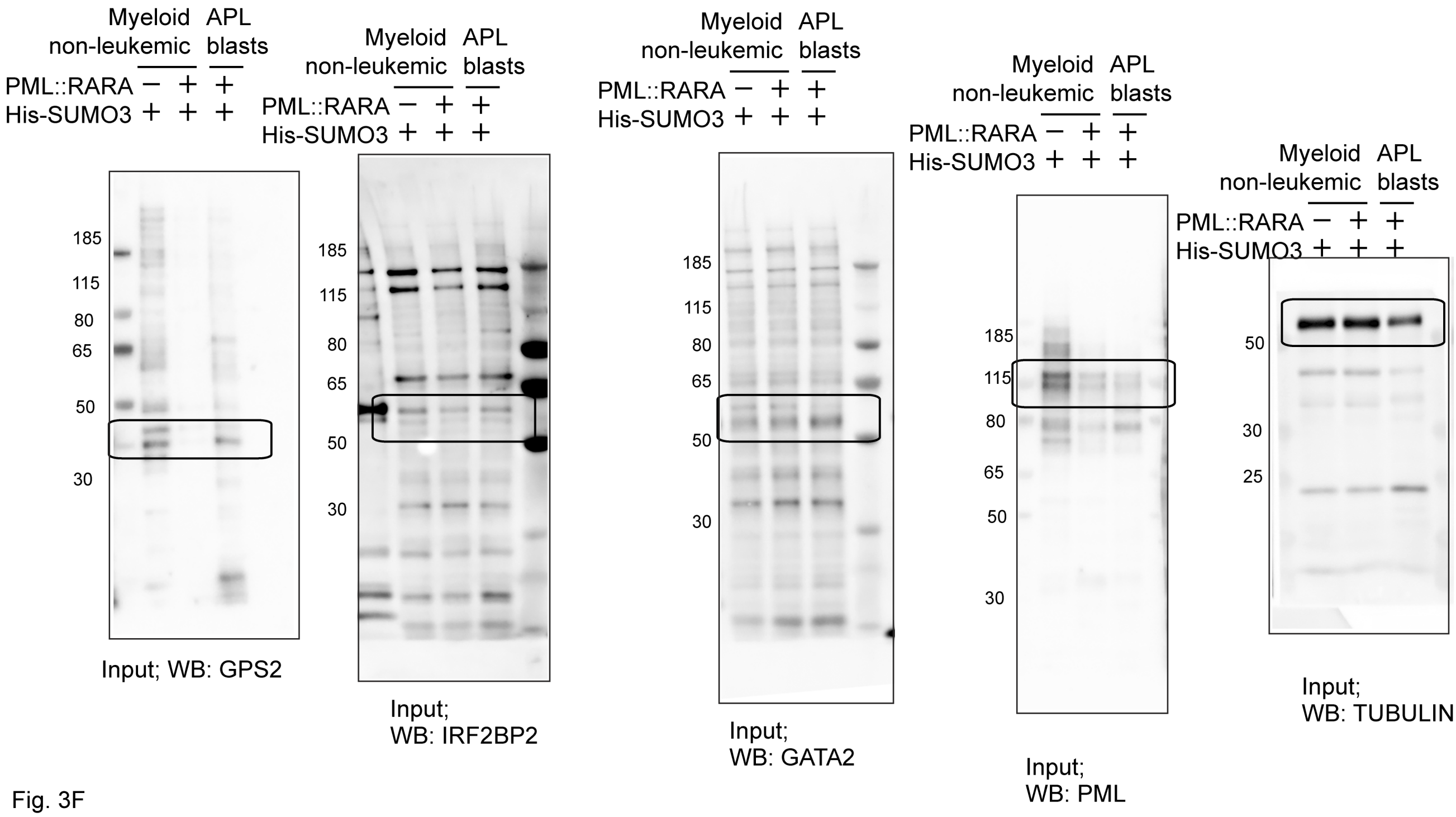
Myeloid non-leukemic
APL blasts
PML::RARA − + +
His-SUMO3 + + +
185
115
80
65
50
30
Input; WB: GPS2
Myeloid non-leukemic
APL blasts
PML::RARA − + +
His-SUMO3 + + +
185
115
80
65
50
30
Input; WB: IRF2BP2
Myeloid non-leukemic
APL blasts
PML::RARA − + +
His-SUMO3 + + +
185
115
80
65
50
30
Input; WB: GATA2
Myeloid non-leukemic
APL blasts
PML::RARA − + +
His-SUMO3 + + +
185
115
80
65
50
30
Input; WB: PML
Myeloid non-leukemic
APL blasts
PML::RARA − + +
His-SUMO3 + + +
50
30
25
Input; WB: TUBULIN

Fig. 3F

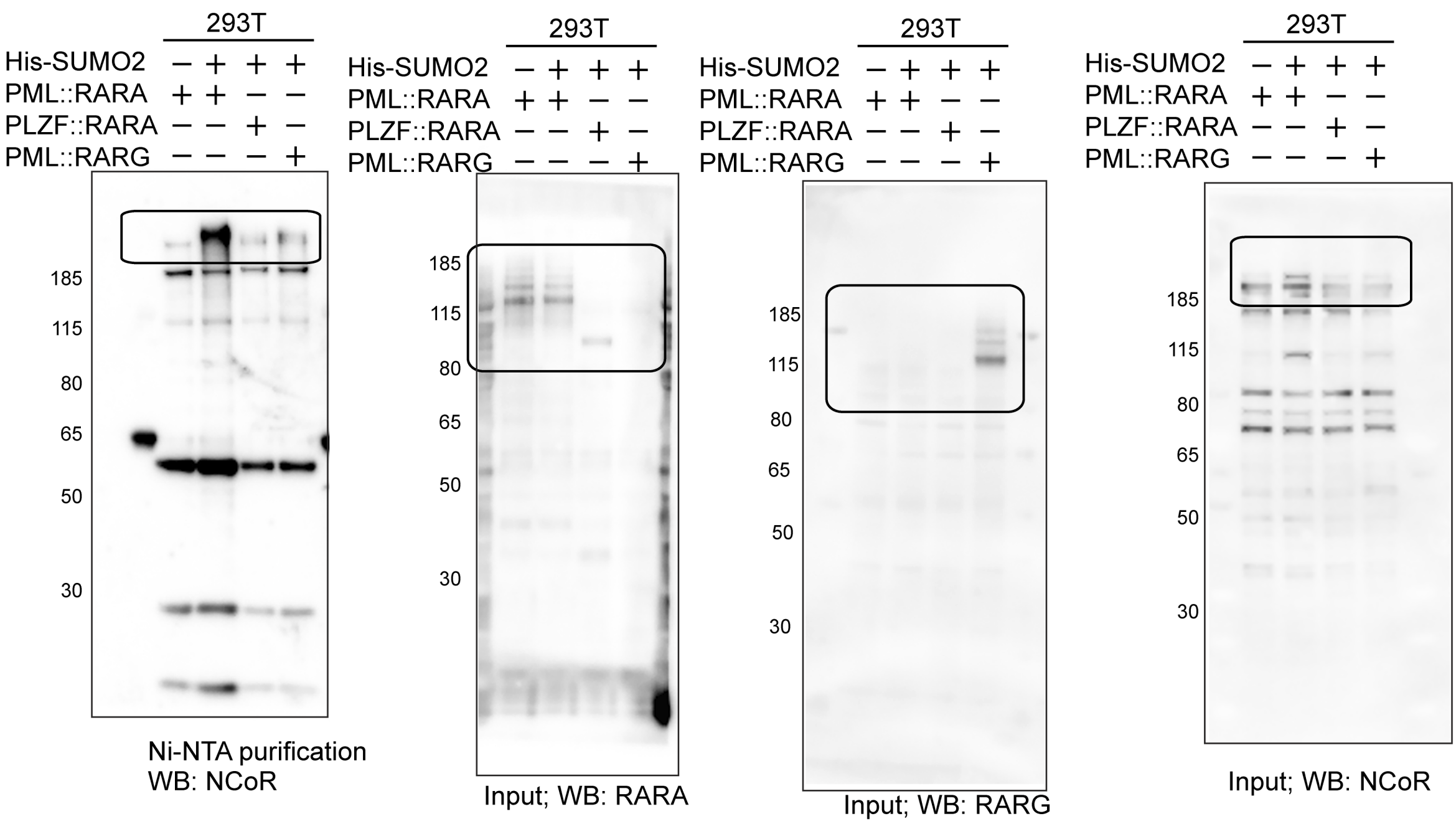
293T
His-SUMO2 − + + +
PML::RARA + + − −
PLZF::RARA − − + −
PML::RARG − − − +
185
115
80
65
50
30
Ni-NTA purification
WB: NCoR
293T
His-SUMO2 − + + +
PML::RARA + + − −
PLZF::RARA − − + −
PML::RARG − − − +
185
115
80
65
50
30
Input; WB: RARA
293T
His-SUMO2 − + + +
PML::RARA + + − −
PLZF::RARA − − + −
PML::RARG − − − +
185
115
80
65
50
30
Input; WB: RARG
293T
His-SUMO2 − + + +
PML::RARA + + − −
PLZF::RARA − − + −
PML::RARG − − − +
185
115
80
65
50
30
Input; WB: NCoR

Fig. 3G

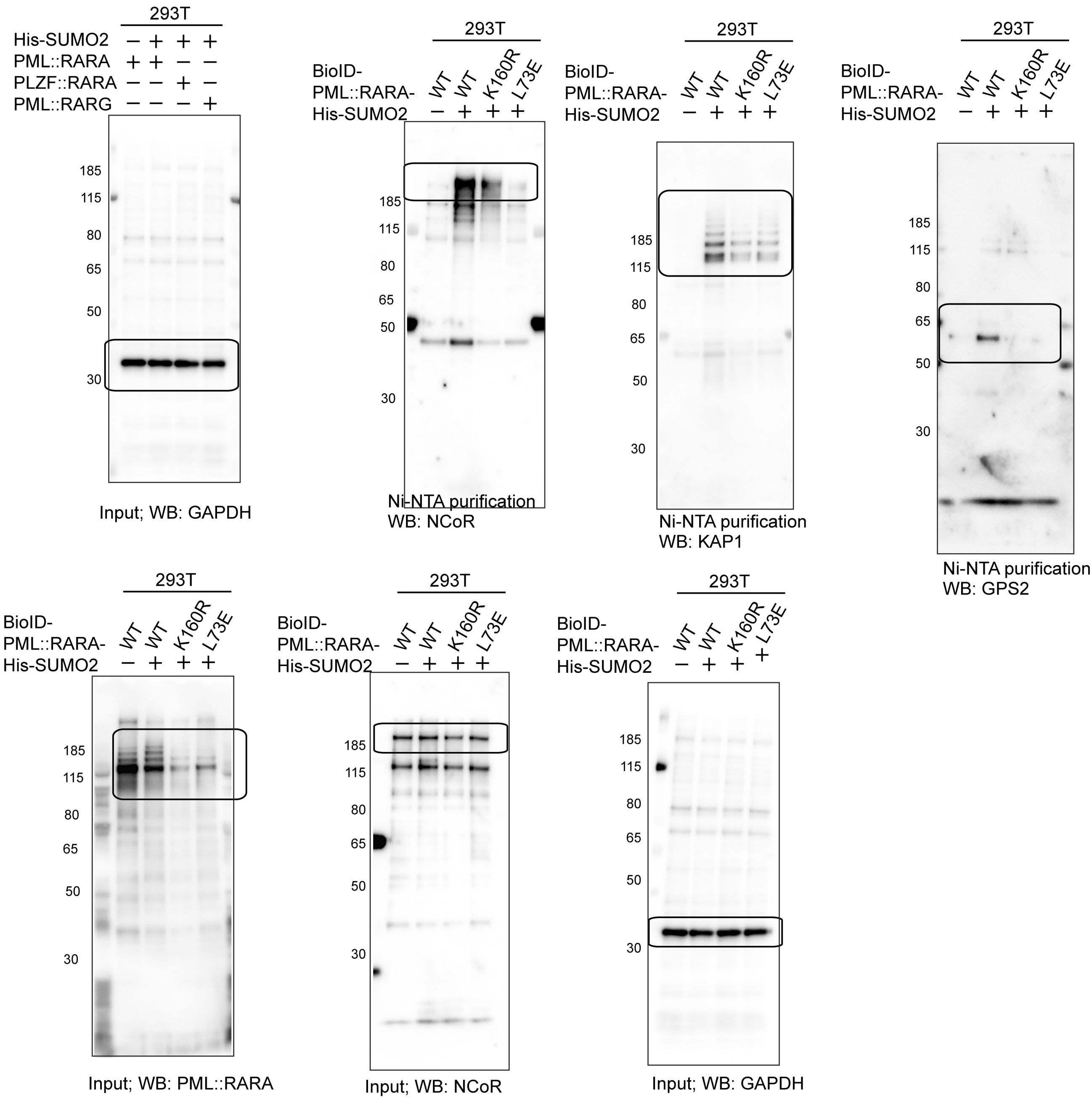
293T
His-SUMO2 − + + +
PML::RARA + + − −
PLZF::RARA − − + −
PML::RARG − − − +
185
115
80
65
50
30
Input; WB: GAPDH
293T
BioID-
PML::RARA- WT WT K160R L73E
His-SUMO2 − + + +
185
115
80
65
50
30
Ni-NTA purification
WB: NCoR
Ni-NTA purification
WB: KAP1
Ni-NTA purification
WB: GPS2
Input; WB: PML::RARA
Input; WB: NCoR
Input; WB: GAPDH
