# Supplementary material for: PML-driven sumoylation of PML::RARA-bound repressors drives hematopoietic progenitor immortalization
Source: J Exp Med. 2026 Aug 28;223(9):e20252333. doi: 10.1084/jem.20252333 (PMC13523716; doi:10.1084/jem.20252333)
Supplement: Supplementary file 5 [file jem_20252333_sourcedatafs1.pdf]

Fig S1B

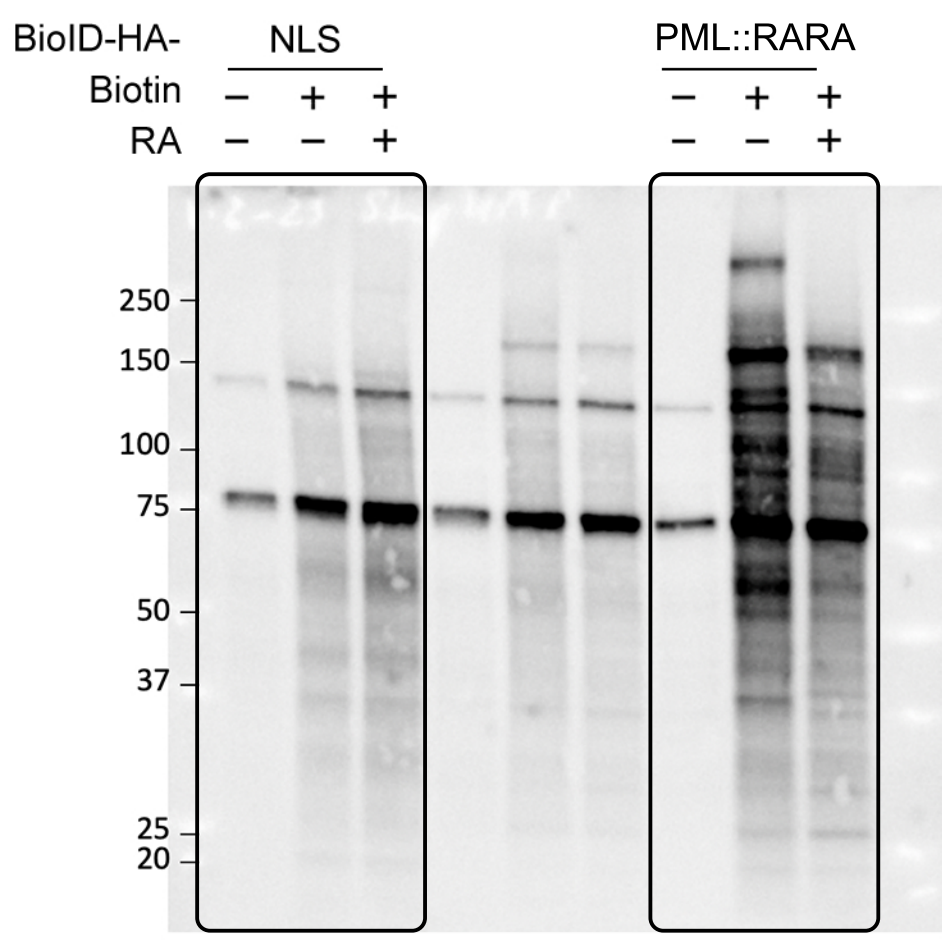

Streptavidin pull-down
WB: Strep-HRP

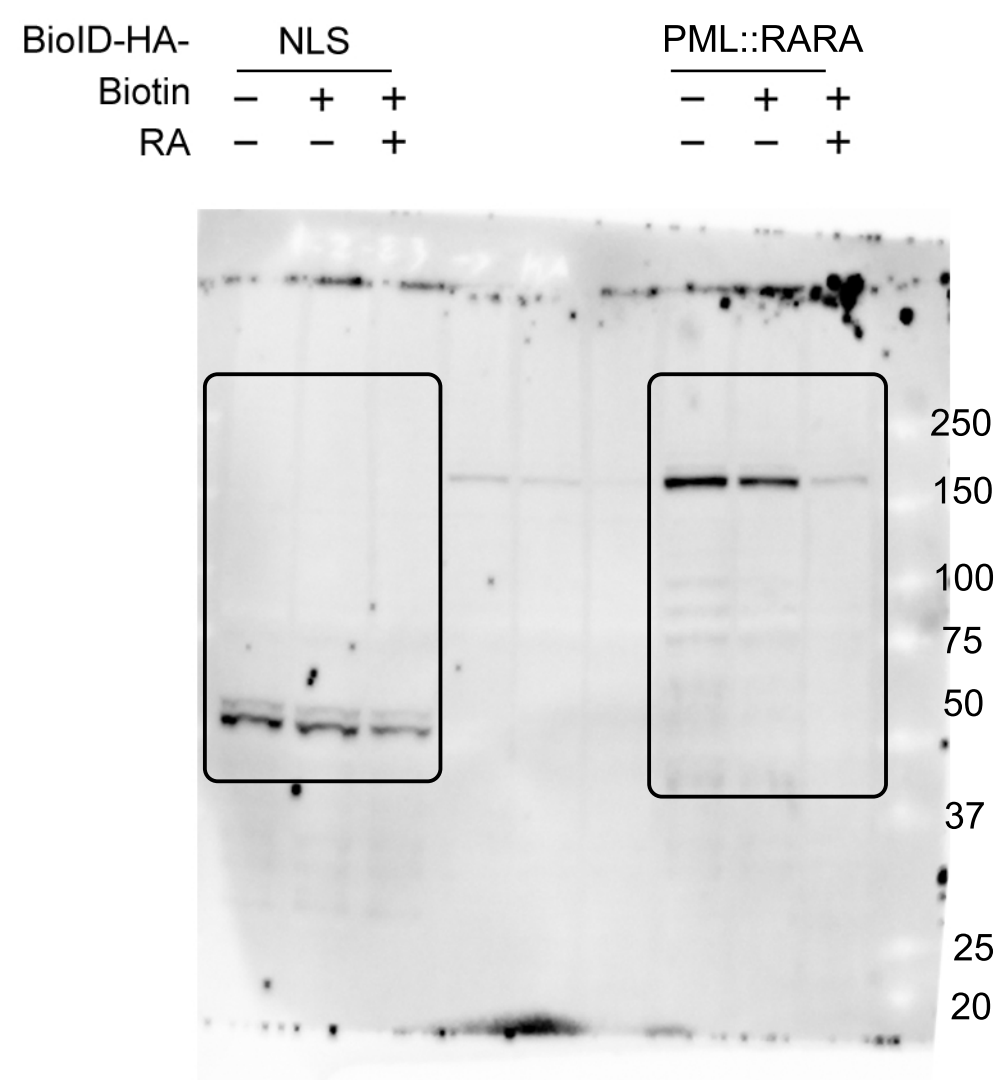

Input
WB: HA
