# Supplementary material for: PML-driven sumoylation of PML::RARA-bound repressors drives hematopoietic progenitor immortalization
Source: J Exp Med. 2026 Aug 28;223(9):e20252333. doi: 10.1084/jem.20252333 (PMC13523716; doi:10.1084/jem.20252333)

Figure S2A

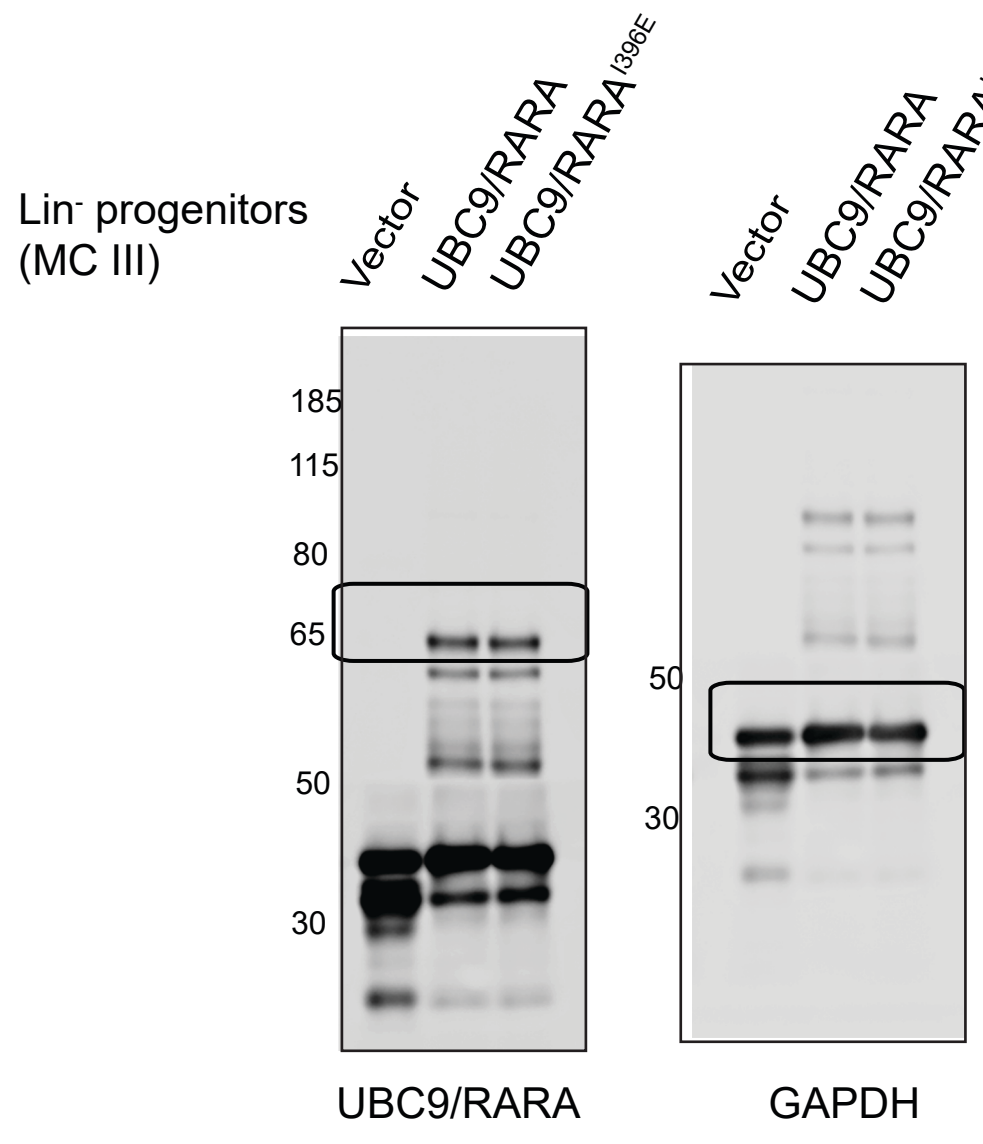

Figure S2D

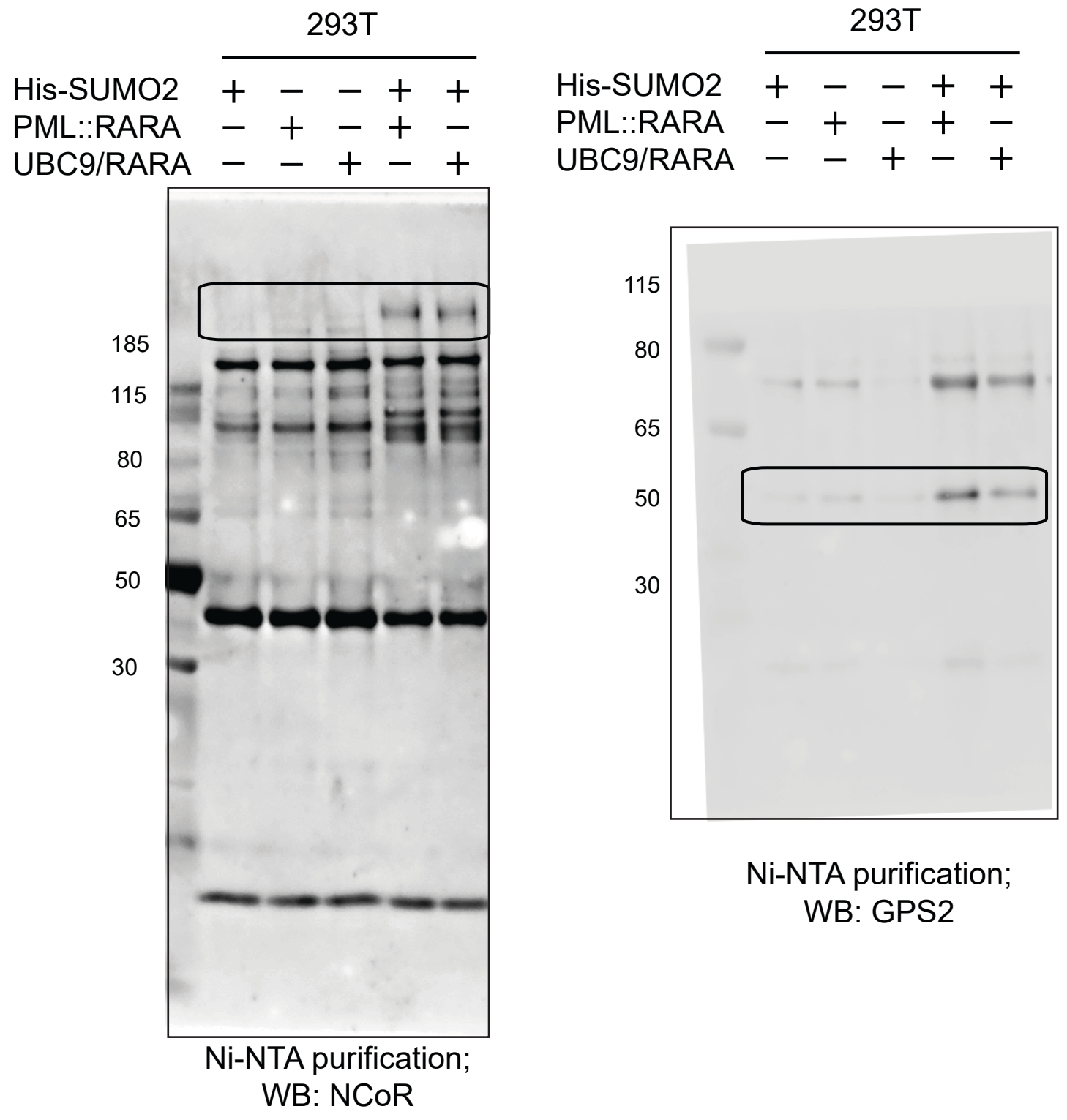

Figure S2D

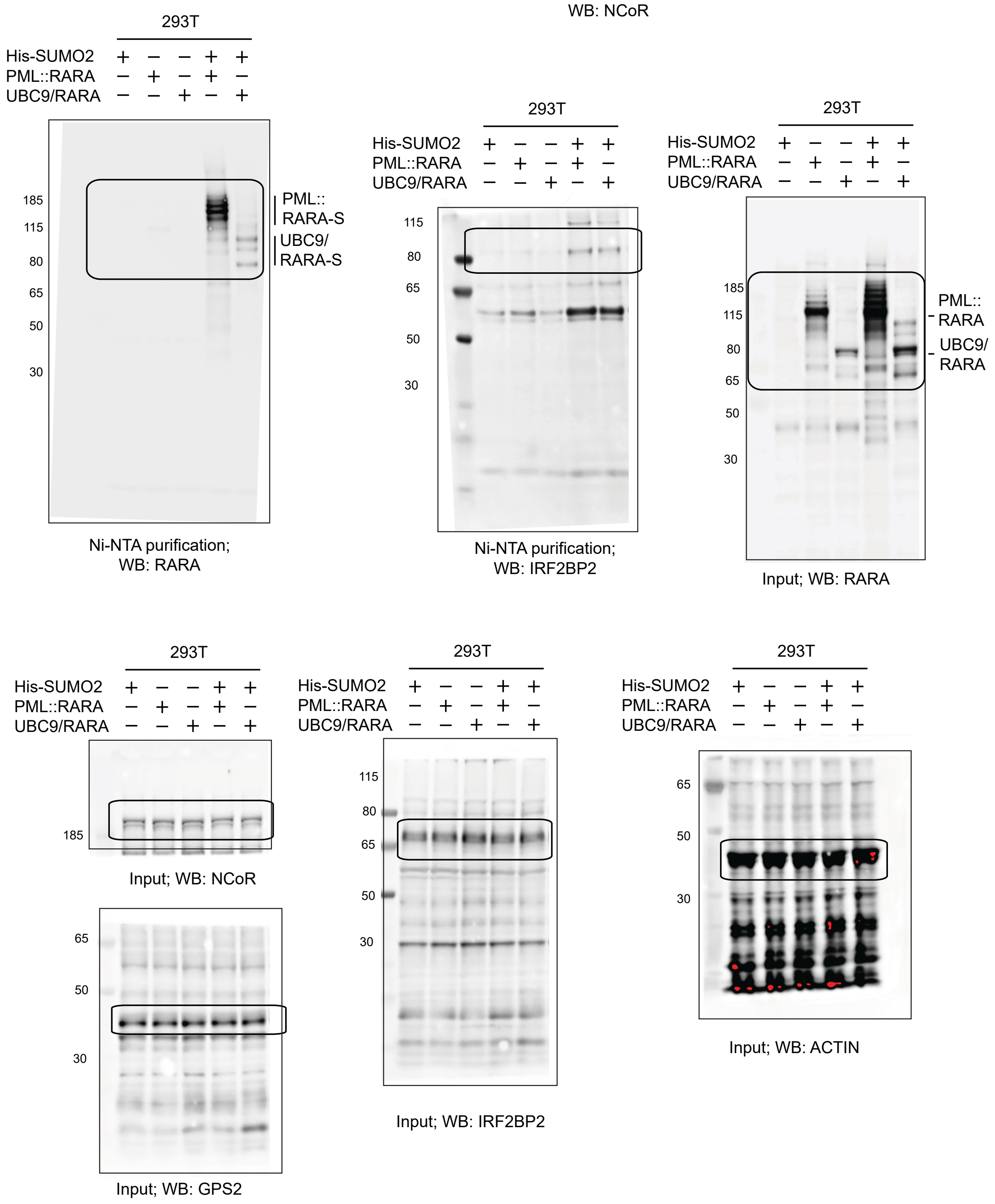

Supplement: Supplementary file 6 [file jem_20252333_sourcedatafs2.pdf]
