# Supplementary material for: PML-driven sumoylation of PML::RARA-bound repressors drives hematopoietic progenitor immortalization
Source: J Exp Med. 2026 Aug 28;223(9):e20252333. doi: 10.1084/jem.20252333 (PMC13523716; doi:10.1084/jem.20252333)
Supplement: Supplementary file 7 [file jem_20252333_tables1.docx]

**Table S1.** List of 379 significant PML/RARA associated protein identified in HPC7 cell lines (FDR < 0.05).

| **Rank** | **Proteins** | **Log2 fold change (Biotin_vs_NLS)** | **P_val (Biotin_vs_NLS)** |
| --- | --- | --- | --- |
| 1 | SKI | 6,879607174 | 4,88576E-06 |
| 2 | NCOR1 | 6,866833464 | 3,12364E-06 |
| 3 | RARA | 6,720842798 | 4,60254E-05 |
| 4 | GNA12 | 6,507993892 | 4,74176E-07 |
| 5 | NCOA3 | 6,279843205 | 1,45842E-05 |
| 6 | JMJD1C | 6,175046281 | 1,08434E-05 |
| 7 | NRIP1 | 5,977958386 | 2,98407E-05 |
| 8 | GPS2 | 5,968209504 | 1,39636E-05 |
| 9 | NCOR2 | 5,937384737 | 7,21646E-06 |
| 10 | RXRA | 5,575840665 | 7,04235E-06 |
| 11 | RARB | 5,5041913 | 0,000891768 |
| 12 | NCOA2 | 5,161287098 | 5,85188E-05 |
| 13 | HIVEP2 | 5,150780665 | 5,07109E-05 |
| 14 | SUMO1 | 4,730620321 | 1,66926E-05 |
| 15 | PML | 4,628888992 | 4,43261E-05 |
| 16 | SUMO2; SUMO3 | 4,538933599 | 5,44501E-05 |
| 17 | IRF2BP2 | 4,26906751 | 2,9995E-05 |
| 18 | FOXK2 | 4,171909791 | 0,000146093 |
| 19 | STING1 | 3,908078076 | 1,42788E-05 |
| 20 | PIAS2 | 3,888052783 | 0,00081648 |
| 21 | MAFF | 3,822870199 | 5,43237E-05 |
| 22 | NENF | 3,783787681 | 2,84015E-05 |
| 23 | SULT1B1 | 3,717487295 | 0,002826687 |
| 24 | SENP6 | 3,716840769 | 2,49627E-05 |
| 25 | PIAS1 | 3,685245256 | 0,000305671 |
| 26 | NR2C2 | 3,659737657 | 0,000154876 |
| 27 | LRPAP1 | 3,574827988 | 8,73383E-05 |
| 28 | ZBTB33 | 3,56727819 | 0,000126377 |
| 29 | ZMYM2 | 3,535079727 | 0,000950782 |
| 30 | HDAC5 | 3,460989451 | 0,000868026 |
| 31 | ASXL2 | 3,45630982 | 7,08497E-05 |
| 32 | CIC | 3,435248716 | 0,000100234 |
| 33 | SELENOF | 3,431546379 | 5,99634E-05 |
| 34 | CBFA2T2 | 3,406940923 | 0,009703465 |
| 35 | RXRB | 3,395876878 | 0,001128531 |
| 36 | IRF2BP1 | 3,395011436 | 0,000283718 |
| 37 | MED15 | 3,36208649 | 0,00037676 |
| 38 | TMED7 | 3,335769879 | 4,70906E-05 |
| 39 | KATNAL2 | 3,277151036 | 0,000256677 |
| 40 | ZNF148 | 3,260543688 | 0,001732032 |
| 41 | MEF2D | 3,250524687 | 0,000680216 |
| 42 | CSNK1A1 | 3,190076128 | 0,000161965 |
| 43 | S100A14 | 3,140730243 | 0,000146706 |
| 44 | NACC1 | 3,13223072 | 6,56711E-05 |
| 45 | MAML1 | 3,128944018 | 0,000506385 |
| 46 | SAMM50 | 3,123848478 | 7,82237E-05 |
| 47 | ATRX | 3,122743278 | 7,19921E-05 |
| 48 | ARAF | 3,117084699 | 0,000301889 |
| 49 | TCF12 | 3,087820886 | 0,000514632 |
| 50 | SLC25A35 | 3,086584126 | 0,006749667 |
| 51 | UPF1 | 3,079035918 | 8,69495E-05 |
| 52 | SVIL | 3,064927995 | 8,13565E-05 |
| 53 | OPA3 | 3,055797606 | 0,002414259 |
| 54 | RAD54L2 | 3,029073063 | 0,000903931 |
| 55 | NCOA1 | 3,012287397 | 0,000165163 |
| 56 | EMILIN1 | 2,958881021 | 0,000146259 |
| 57 | TMED4 | 2,945336828 | 0,000159633 |
| 58 | TIA1 | 2,918069985 | 0,000162401 |
| 59 | VPS4B | 2,911545262 | 0,000570667 |
| 60 | ETV6 | 2,902081442 | 0,000235614 |
| 61 | AFG3L1 | 2,896427834 | 0,000561655 |
| 62 | MTCH2 | 2,888129394 | 0,001720883 |
| 63 | AP2A1 | 2,878251007 | 0,010308545 |
| 64 | COQ4 | 2,84777724 | 0,000616838 |
| 65 | MRPL21 | 2,819798166 | 0,000982829 |
| 66 | VRK1 | 2,814917653 | 0,00071635 |
| 67 | MTPAP | 2,802157774 | 0,002052881 |
| 68 | MED24 | 2,791592322 | 0,009097346 |
| 69 | FOXRED1 | 2,784530506 | 0,00070936 |
| 70 | GNA11 | 2,776558014 | 0,000239484 |
| 71 | FOXK1 | 2,759895924 | 0,003406209 |
| 72 | TLE3 | 2,747228894 | 0,000508842 |
| 73 | COLGALT1 | 2,724946612 | 0,00062172 |
| 74 | PTP4A1 | 2,705484273 | 0,000387222 |
| 75 | UCHL5 | 2,701223671 | 0,000896535 |
| 76 | CTBP2 | 2,662865399 | 0,004002346 |
| 77 | NXT1 | 2,661376962 | 0,000280768 |
| 78 | RAVER1 | 2,65763464 | 0,000393577 |
| 79 | MAML3 | 2,63965646 | 0,000204884 |
| 80 | RPS6KA1 | 2,633509825 | 0,001889734 |
| 81 | B2M | 2,627196706 | 0,00746666 |
| 82 | WDR3 | 2,625363461 | 0,001233376 |
| 83 | ACOT7 | 2,620091924 | 0,001327941 |
| 84 | MED1 | 2,604755699 | 0,018741993 |
| 85 | UAP1L1 | 2,601958677 | 0,00025881 |
| 86 | AP1G1 | 2,5973832 | 0,000329759 |
| 87 | NFIA | 2,587992691 | 0,000330111 |
| 88 | ANXA11 | 2,585602368 | 0,001015807 |
| 89 | HACD3 | 2,584468656 | 0,000293364 |
| 90 | SRP68 | 2,584080926 | 0,000546324 |
| 91 | MLKL | 2,580873534 | 0,00483155 |
| 92 | ZC3HAV1 | 2,563681776 | 0,000981006 |
| 93 | GM6020 | 2,560921065 | 0,000519664 |
| 94 | DDX42 | 2,553653748 | 0,007728199 |
| 95 | SLC25A33 | 2,548149353 | 0,010331567 |
| 96 | PTPRE | 2,546823925 | 0,000498315 |
| 97 | XPNPEP1 | 2,525659651 | 0,00336251 |
| 98 | TET2 | 2,52202597 | 0,001045924 |
| 99 | CDK7 | 2,508285316 | 0,000352102 |
| 100 | CCDC12 | 2,505732477 | 0,001385176 |
| 101 | NDUFS3 | 2,502882663 | 0,00092863 |
| 102 | TIMM23 | 2,495614342 | 0,000413455 |
| 103 | AGK | 2,492905719 | 0,000391632 |
| 104 | PML/RARA | 2,490259795 | 0,013473314 |
| 105 | ATXN1L | 2,483837205 | 0,003258086 |
| 106 | MAL2 | 2,477184908 | 0,003105585 |
| 107 | MAPK10 | 2,476948323 | 0,004521266 |
| 108 | TRIM24 | 2,469643909 | 0,001251114 |
| 109 | OLA1 | 2,445412248 | 0,00068003 |
| 110 | P4HB | 2,412676308 | 0,012755535 |
| 111 | GLTP | 2,404578877 | 0,000577675 |
| 112 | RAB4B | 2,404497396 | 0,000430088 |
| 113 | TBL1X | 2,40329895 | 0,000591562 |
| 114 | PTBP2 | 2,402733205 | 0,012317497 |
| 115 | ACOT1 | 2,398192749 | 0,001285911 |
| 116 | ABCD1 | 2,397612382 | 0,000601698 |
| 117 | DHRS1 | 2,387869864 | 0,004234116 |
| 118 | WDR36 | 2,385773631 | 0,000509254 |
| 119 | DVL1 | 2,383180184 | 0,001625453 |
| 120 | ATF7IP | 2,380981016 | 0,006544731 |
| 121 | L2HGDH | 2,380513084 | 0,000963228 |
| 122 | TM9SF3 | 2,376611948 | 0,000491792 |
| 123 | API5 | 2,375834655 | 0,014541973 |
| 124 | MRPS22 | 2,373411167 | 0,001304315 |
| 125 | PTPN2 | 2,366430847 | 0,001796915 |
| 126 | MRPL43 | 2,364377254 | 0,021441532 |
| 127 | UGGT1 | 2,322192912 | 0,001324121 |
| 128 | MRPL37 | 2,306896634 | 0,015316384 |
| 129 | MTND1 | 2,289578444 | 0,01391429 |
| 130 | HDHD5 | 2,284393793 | 0,001967644 |
| 131 | PDS5B | 2,279549024 | 0,000575164 |
| 132 | ERO1A | 2,278694247 | 0,006643302 |
| 133 | MRPS30 | 2,277701058 | 0,007831946 |
| 134 | BPIFA3 | 2,26755446 | 0,00088465 |
| 135 | PSMC5 | 2,266896964 | 0,006343619 |
| 136 | NMT1 | 2,229588237 | 0,001695846 |
| 137 | LIMD2 | 2,228715118 | 0,002985588 |
| 138 | AKR7A2 | 2,227529915 | 0,011021313 |
| 139 | NT5DC2 | 2,211856886 | 0,020069636 |
| 140 | MRPL44 | 2,206923966 | 0,001255606 |
| 141 | PRDX4 | 2,202905504 | 0,002329069 |
| 142 | OGFOD3 | 2,201358972 | 0,001849936 |
| 143 | BUD31 | 2,197381203 | 0,001665953 |
| 144 | ANXA6 | 2,191603954 | 0,000979654 |
| 145 | FBL | 2,184152528 | 0,019036998 |
| 146 | ERG | 2,178933452 | 0,011303316 |
| 147 | TARS2 | 2,177454443 | 0,000829217 |
| 148 | FLI1 | 2,160442775 | 0,016151727 |
| 149 | TMEM126B | 2,159862944 | 0,017075081 |
| 150 | MCL1 | 2,15767632 | 0,011903692 |
| 151 | CYB5R3 | 2,155245886 | 0,005842167 |
| 152 | LPCAT3 | 2,152177944 | 0,000767744 |
| 153 | RBMX | 2,146743602 | 0,007000164 |
| 154 | GNAS | 2,144154613 | 0,00240919 |
| 155 | CTBP1 | 2,142950281 | 0,002903131 |
| 156 | TMEM70 | 2,133626231 | 0,002317884 |
| 157 | CSNK2B | 2,122533812 | 0,000815557 |
| 158 | NUDT16L1 | 2,118783766 | 0,012481279 |
| 159 | PDE12 | 2,118126902 | 0,003975527 |
| 160 | RAB27A | 2,115079201 | 0,001281883 |
| 161 | EIF6 | 2,108317234 | 0,0016808 |
| 162 | ALAD | 2,101910604 | 0,014624374 |
| 163 | APOOL | 2,087437486 | 0,002381855 |
| 164 | MRPL38 | 2,08067757 | 0,003000925 |
| 165 | CDK6 | 2,077936231 | 0,001741009 |
| 166 | TBCD | 2,077258109 | 0,001162339 |
| 167 | NXF1 | 2,076288489 | 0,005967336 |
| 168 | RNF220 | 2,070883602 | 0,007778557 |
| 169 | ICAM2 | 2,070190405 | 0,007261921 |
| 170 | LETM1 | 2,064924526 | 0,005517403 |
| 171 | GNPAT | 2,063518683 | 0,003249582 |
| 172 | CFAP20 | 2,062008826 | 0,002846994 |
| 173 | LAMP1 | 2,061683786 | 0,002392234 |
| 174 | NDUFS7 | 2,049735102 | 0,003799385 |
| 175 | NDUFS2 | 2,048002055 | 0,003550508 |
| 176 | NAA15 | 2,047972697 | 0,001867321 |
| 177 | ECI1 | 2,033862433 | 0,001729702 |
| 178 | ABCB10 | 2,030163388 | 0,01789966 |
| 179 | H2BC21 | 2,028658107 | 0,006974508 |
| 180 | MOV10 | 2,010081439 | 0,001910052 |
| 181 | NIPSNAP1 | 2,005181961 | 0,00299183 |
| 182 | CCT3 | 1,996328127 | 0,001846007 |
| 183 | PUS1 | 1,9933171 | 0,002529556 |
| 184 | PSMD2 | 1,99263914 | 0,001201344 |
| 185 | VPS29 | 1,981792439 | 0,002101686 |
| 186 | CDIPT | 1,979885309 | 0,002243325 |
| 187 | GSR | 1,97909473 | 0,006909959 |
| 188 | ILKAP | 1,976857576 | 0,004106833 |
| 189 | MRPL22 | 1,975297446 | 0,017659552 |
| 190 | LGALS9 | 1,96579586 | 0,014275567 |
| 191 | CDC16 | 1,962741096 | 0,009851924 |
| 192 | MATR3 | 1,962251892 | 0,006135625 |
| 193 | SFXN3 | 1,960682194 | 0,00785162 |
| 194 | POLR2B | 1,956218734 | 0,004844849 |
| 195 | RAB9A | 1,955331149 | 0,023252779 |
| 196 | NDUFB7 | 1,950568864 | 0,001378902 |
| 197 | PIAS4 | 1,949708408 | 0,003619063 |
| 198 | BAK1 | 1,937581656 | 0,021531787 |
| 199 | CSTF3 | 1,935843263 | 0,006790575 |
| 200 | WDR43 | 1,932675461 | 0,001755486 |
| 201 | VPS35 | 1,929774233 | 0,018906348 |
| 202 | ANP32C | 1,927579057 | 0,00587287 |
| 203 | SUPT5H | 1,920178248 | 0,002717585 |
| 204 | RAB5C | 1,920151222 | 0,003889586 |
| 205 | PNPO | 1,917643543 | 0,014641316 |
| 206 | MTERF4 | 1,914322423 | 0,002622398 |
| 207 | RRM2 | 1,913014024 | 0,005538234 |
| 208 | SSBP2 | 1,911766808 | 0,003023717 |
| 209 | RAC1 | 1,908659235 | 0,017484337 |
| 210 | GANAB | 1,900508336 | 0,003340969 |
| 211 | LIN54 | 1,895375684 | 0,022713111 |
| 212 | EP400 | 1,890991894 | 0,003548746 |
| 213 | CALR | 1,889936689 | 0,00659307 |
| 214 | NAE1 | 1,88666302 | 0,01766087 |
| 215 | PRPSAP2 | 1,883805459 | 0,006758861 |
| 216 | DOK2 | 1,863586701 | 0,004727903 |
| 217 | TRAPPC5 | 1,863391492 | 0,007257085 |
| 218 | LARS1 | 1,854268771 | 0,022049006 |
| 219 | GALNT2 | 1,833178224 | 0,009639877 |
| 220 | YIF1B | 1,829261134 | 0,007692327 |
| 221 | DHX9 | 1,82652868 | 0,010027738 |
| 222 | EPM2AIP1 | 1,812970355 | 0,009254941 |
| 223 | COQ7 | 1,81260415 | 0,020428667 |
| 224 | RAB6A | 1,810736284 | 0,012159337 |
| 225 | TMED10 | 1,810271974 | 0,006212517 |
| 226 | SLC29A1 | 1,810067029 | 0,007382577 |
| 227 | ADNP | 1,804204986 | 0,002209996 |
| 228 | MARS-02 | 1,783104671 | 0,003599682 |
| 229 | PDIA3 | 1,773008552 | 0,010562708 |
| 230 | RAB4A | 1,772393868 | 0,010067776 |
| 231 | LNPEP | 1,768015801 | 0,003276194 |
| 232 | ERAP1 | 1,762332372 | 0,007450136 |
| 233 | RAB8B | 1,760810274 | 0,006027171 |
| 234 | ACLY | 1,754404035 | 0,010628233 |
| 235 | PSMD12 | 1,753636503 | 0,008503731 |
| 236 | HLCS | 1,752698045 | 0,00872763 |
| 237 | TMEM33 | 1,74538307 | 0,009437696 |
| 238 | CLIC4 | 1,7411868 | 0,024058264 |
| 239 | GET3 | 1,739048405 | 0,024702947 |
| 240 | SAE1 | 1,722253427 | 0,005073818 |
| 241 | PRKACB | 1,715280431 | 0,007214441 |
| 242 | FEN1 | 1,710569804 | 0,00670164 |
| 243 | SAR1A | 1,706467327 | 0,009144458 |
| 244 | BROX | 1,70583872 | 0,021529179 |
| 245 | CPNE3 | 1,700449992 | 0,017748312 |
| 246 | DLST | 1,698210959 | 0,014565497 |
| 247 | SSR4 | 1,698085705 | 0,021980429 |
| 248 | MRPS27 | 1,686406915 | 0,011489717 |
| 249 | PHB2 | 1,68057178 | 0,016432504 |
| 250 | TIMM50 | 1,679044461 | 0,012296328 |
| 251 | DDX5 | 1,677498243 | 0,00911563 |
| 252 | TRMT10C | 1,66933176 | 0,00529177 |
| 253 | ARPC4 | 1,668568735 | 0,006185859 |
| 254 | EIF3D | 1,667786574 | 0,008843985 |
| 255 | LRRC59 | 1,667021095 | 0,011887259 |
| 256 | KCNAB2 | 1,65515598 | 0,005766413 |
| 257 | IMMT | 1,653324421 | 0,004748669 |
| 258 | MARS-01 | 1,647431411 | 0,024406812 |
| 259 | AP3M1 | 1,645132974 | 0,021082427 |
| 260 | UQCC2 | 1,643048769 | 0,006135938 |
| 261 | SYNCRIP | 1,630832056 | 0,011971227 |
| 262 | DDOST | 1,627700483 | 0,010335913 |
| 263 | NDUFA13 | 1,617107488 | 0,010993339 |
| 264 | RAB21 | 1,614748863 | 0,004080245 |
| 265 | OTUB1 | 1,60958686 | 0,00772064 |
| 266 | CS | 1,604927844 | 0,01124329 |
| 267 | RAB2A | 1,600821761 | 0,023585118 |
| 268 | TMX1 | 1,597812103 | 0,013637434 |
| 269 | RTN3 | 1,594467002 | 0,008610146 |
| 270 | DNAJC8 | 1,594011927 | 0,007496253 |
| 271 | CPNE1 | 1,592608237 | 0,005640588 |
| 272 | MAML2 | 1,588901322 | 0,004812623 |
| 273 | HNRNPH1 | 1,587281513 | 0,013940127 |
| 274 | ABCB7 | 1,583540588 | 0,015349456 |
| 275 | FARSA | 1,578707818 | 0,007842525 |
| 276 | RNMT | 1,575592758 | 0,018556687 |
| 277 | AP2B1 | 1,575134228 | 0,02199109 |
| 278 | PTPN11 | 1,571663392 | 0,004692811 |
| 279 | RCC2 | 1,560361844 | 0,018159294 |
| 280 | SF3B3 | 1,549862518 | 0,008082808 |
| 281 | WAS | 1,549685077 | 0,015505858 |
| 282 | NUP93 | 1,549597236 | 0,006010662 |
| 283 | NCKAP1 | 1,534596624 | 0,016465895 |
| 284 | ISCU | 1,534341423 | 0,010565995 |
| 285 | DDX19A | 1,532412489 | 0,010246514 |
| 286 | ZHX2 | 1,52876956 | 0,012435034 |
| 287 | NIPSNAP2 | 1,514154147 | 0,023283132 |
| 288 | PTBP1 | 1,512832362 | 0,017967328 |
| 289 | VDAC3 | 1,5104449 | 0,009614993 |
| 290 | DNAJC11 | 1,509249475 | 0,007039766 |
| 291 | PDK3 | 1,504992469 | 0,019868119 |
| 292 | POLD1 | 1,497712059 | 0,006412613 |
| 293 | SNRPA1 | 1,497163662 | 0,007439542 |
| 294 | CUL3 | 1,495896313 | 0,007030072 |
| 295 | EIF4A3 | 1,494528785 | 0,012730983 |
| 296 | MTCO2 | 1,493926271 | 0,02139376 |
| 297 | PHGDH | 1,490448207 | 0,018469871 |
| 298 | HELLS | 1,490187443 | 0,021641834 |
| 299 | ATXN10 | 1,488732631 | 0,017816849 |
| 300 | TIMM44 | 1,488198022 | 0,019837763 |
| 301 | ATAD3 | 1,486787656 | 0,022962081 |
| 302 | TMEM11 | 1,485202396 | 0,023826801 |
| 303 | SBNO1 | 1,483192307 | 0,006546929 |
| 304 | PDCD10 | 1,48304053 | 0,014205535 |
| 305 | DYNC1H1 | 1,469771976 | 0,024351919 |
| 306 | ARHGAP18 | 1,46900753 | 0,014394254 |
| 307 | NUP214 | 1,467298048 | 0,018950071 |
| 308 | EEF1G | 1,46715043 | 0,008615095 |
| 309 | PGRMC2 | 1,465914689 | 0,012620604 |
| 310 | NNT | 1,457974064 | 0,019556757 |
| 311 | ARF4 | 1,441414422 | 0,009448101 |
| 312 | YTHDF2 | 1,43789866 | 0,011247185 |
| 313 | HEATR1 | 1,432738192 | 0,012814802 |
| 314 | ARHGAP1 | 1,431822424 | 0,015129917 |
| 315 | DRG1 | 1,428116676 | 0,012587434 |
| 316 | RPN2 | 1,4239749 | 0,021940129 |
| 317 | SIRT3 | 1,423526025 | 0,010882787 |
| 318 | DDB1 | 1,418124078 | 0,009916488 |
| 319 | MORC3 | 1,414998714 | 0,023550608 |
| 320 | ARL1 | 1,414302172 | 0,017440803 |
| 321 | LYN | 1,413812122 | 0,016109514 |
| 322 | SNRNP200 | 1,404564853 | 0,020119072 |
| 323 | DPP3 | 1,402352077 | 0,013821042 |
| 324 | RPL27 | 1,40127343 | 0,023223934 |
| 325 | GNB1 | 1,398164774 | 0,018172625 |
| 326 | HSDL1 | 1,393133003 | 0,018442613 |
| 327 | HARS2 | 1,391681399 | 0,012297775 |
| 328 | RPS13 | 1,390204378 | 0,024044211 |
| 329 | SPDL1 | 1,386725541 | 0,0186354 |
| 330 | MOB2 | 1,382612359 | 0,019324679 |
| 331 | EIF5A | 1,378697316 | 0,015568445 |
| 332 | EIF4A1 | 1,37574862 | 0,011964434 |
| 333 | EMSY | 1,367689389 | 0,023056522 |
| 334 | DDX39A | 1,365320962 | 0,009989872 |
| 335 | EP300 | 1,363699672 | 0,018909051 |
| 336 | KPNB1 | 1,363262402 | 0,024578656 |
| 337 | ATP1A1 | 1,36197438 | 0,01768424 |
| 338 | STAT5A | 1,36064792 | 0,016989379 |
| 339 | TMED9 | 1,356352685 | 0,014937629 |
| 340 | PRR12 | 1,350894998 | 0,013342475 |
| 341 | UBA2 | 1,350852586 | 0,021282401 |
| 342 | CLTC | 1,345945597 | 0,022588253 |
| 343 | SLC25A20 | 1,343668507 | 0,020466278 |
| 344 | ATP5PB | 1,339125527 | 0,012158037 |
| 345 | H2AC11 | 1,336436088 | 0,020111071 |
| 346 | DARS1 | 1,335592391 | 0,019413133 |
| 347 | PLEKHF1 | 1,330278669 | 0,012532003 |
| 348 | ZMIZ1 | 1,328024672 | 0,017154684 |
| 349 | PDPR | 1,325424362 | 0,013136703 |
| 350 | NSF | 1,322154021 | 0,013722377 |
| 351 | GSTZ1 | 1,317528268 | 0,024633208 |
| 352 | ACTR3 | 1,316658047 | 0,024867627 |
| 353 | FKBP5 | 1,309867721 | 0,021292379 |
| 354 | RAB35 | 1,30147829 | 0,013107751 |
| 355 | PCBP2 | 1,301272493 | 0,013583409 |
| 356 | MTND4 | 1,294915195 | 0,017370492 |
| 357 | PAICS | 1,290804981 | 0,0172909 |
| 358 | RAC2 | 1,290188615 | 0,017798315 |
| 359 | HSP90B1 | 1,286944893 | 0,024410703 |
| 360 | SNRPB2 | 1,27522189 | 0,020454001 |
| 361 | GDI2 | 1,274806163 | 0,016536185 |
| 362 | SND1 | 1,272741013 | 0,015892534 |
| 363 | GPD2 | 1,271757952 | 0,023118778 |
| 364 | POR | 1,271129565 | 0,017647554 |
| 365 | TARS1 | 1,270890249 | 0,021706013 |
| 366 | CARS2 | 1,26099291 | 0,017422474 |
| 367 | PPIB | 1,255414817 | 0,017627055 |
| 368 | RARS2 | 1,254565589 | 0,017699371 |
| 369 | YME1L1 | 1,244897054 | 0,016872796 |
| 370 | RAB18 | 1,235374348 | 0,021367661 |
| 371 | RAB33B | 1,23067901 | 0,018668022 |
| 372 | CAP1 | 1,218032757 | 0,02167801 |
| 373 | MCM7 | 1,215724652 | 0,020163985 |
| 374 | HSD17B4 | 1,167962475 | 0,025011121 |
| 375 | SSR1 | 1,164125592 | 0,01993242 |
| 376 | RPS27A | 1,163163044 | 0,02423714 |
| 377 | EPRS1 | 1,15599508 | 0,023364667 |
| 378 | LYPLA2 | 1,131501017 | 0,024187746 |
| 379 | PRDX2 | 1,121842154 | 0,023488711 |
