# Supplementary material for: PML-driven sumoylation of PML::RARA-bound repressors drives hematopoietic progenitor immortalization
Source: J Exp Med. 2026 Aug 28;223(9):e20252333. doi: 10.1084/jem.20252333 (PMC13523716; doi:10.1084/jem.20252333)
Supplement: Supplementary file 8 [file jem_20252333_tables2.docx]

**Table S2.** List and reference of plasmid constructs used in the study.

|  | **Vector backbone** | **Gene** | **Species** | **Reference** |
| --- | --- | --- | --- | --- |
| 1 | MSCV-puro | Empty vector |  | Addgene |
| 2 |  | RARA | Human | Zhu et al., 2005 |
| 3 |  | PML/RARA |  |  |
| 4 |  | RARA-I396E |  | Described in the plasmid constructs section of the Materials and methods |
| 5 |  | NLS-DHFR-BioID-HA |  |  |
| 6 |  | DHFR-PML/RARA-BioID2-HA |  |  |
| 7 |  | DHFR-PML/RARA-I396E-BioID2-HA |  |  |
| 8 |  | DHFR-PML/RARA-K160R-BioID2-HA |  |  |
| 9 |  | DHFR-PML/RARA-L73E-BioID2-HA |  |  |
| 10 |  | UBC9-RARA |  |  |
| 11 |  | UBC9-RARA-I396E |  |  |
| 12 | MSCV-neo | Empty vector |  | Addgene |
| 13 |  | PLZF/RARA | Human | Du et al., 1999 |
| 14 |  | Flag-RARB |  | Described in the plasmid constructs section of the Materials and methods |
| 15 |  | Flag-POZ/RARB |  |  |
| 16 | PINCO | PML/RARG |  | A kind gift of Pier Giuseppe Pelicci (European Institute of Oncology IRCCS, Milan, Italy) |
| 17 | MSCV-IRES-EGFP | His10-SUMO1 |  | Sahin et al., 2014 |
| 18 |  | His10-SUMO2 |  |  |
| 19 | pCMV-BioID2-HA | PML/RARA |  | Described in the plasmid constructs section of the Materials and methods |
| 20 | pDR5-3x-TK-FL | Firefly luciferase |  | de Thé et al. 1990 |
| 21 | pRL | Renilla luciferase |  | Zhu et al. 2005 |
